# Forensic Analysis of Degradation Pathways and Physicochemical Changes in Polyamide and Acrylic Fibers Exposed to Various Degrading Agents

**DOI:** 10.3390/polym18192375

**Published:** 2026-09-29

**Authors:** Aleksandra Zahorska, Anna Topolewska, Daria Śmigiel-Kamińska, Jolanta Kumirska

**Affiliations:** 1 Division of Didactics and Popular Science, Faculty of Chemistry, University of Gdansk, Wita Stwosza 63, 80-308 Gdańsk, Poland; 2 Department of Security, Faculty of Law and Administration, University of Business and Administration of E. Kwiatkowski in Gdynia, Kielecka 7, 81-303 Gdynia, Poland

**Keywords:** polyamide fibers, acrylic fibers, degradation mechanisms, physicochemical changes, fiber characterization, forensic examination

## Abstract

Synthetic fibers constitute a cornerstone of the modern textile industry. Among these materials, polyamide and acrylic fibers have achieved widespread application due to their favorable strength-to-weight ratio, chemical resistance, and economic efficiency. Despite their advantageous characteristics, these fibers remain susceptible to various degradation processes. The analysis of fiber damage represents a critical yet complex aspect of textile identification and comparative studies. Accurate determination of degradation mechanisms is essential for assessing material performance, predicting service life, quality control, and supporting forensic investigations. However, distinguishing between different types of damage and identifying their underlying causes remains a significant analytical challenge. In this review paper, the forensic analysis of degradation pathways and physicochemical changes in polyamide and acrylic fibers exposed to various degrading agents is presented. The available literature is critically examined to elucidate the mechanisms governing fiber deterioration and to correlate structural transformations with changes in macroscopic and physicochemical properties. Particular attention is given to the influence of environmental, thermal, chemical, and mechanical stressors on fiber stability and performance. Furthermore, current analytical approaches used for the identification and characterization of degradation phenomena are discussed, highlighting their applicability and limitations in forensic science. To the best of our knowledge, such a comprehensive review has not yet been published.

## 1. Introduction

Synthetic fibers play a vital role across numerous sectors, including apparel and clothing, home furnishings, sports, transport, automotive, architecture, industrial applications, and medicine. Their rapid development throughout the 20th century (the first man-made synthetic polymer fiber was nylon in 1939) was driven by the demand for materials that are durable, lightweight, and cost-effective, offering viable alternatives or enhancements to natural fibers. Advances in polymer chemistry and fiber-forming technologies have enabled the production of synthetic fibers with superior mechanical performance and tailored functional properties. The development of new fibers continues today, as ongoing technical innovations advance fiber and textile manufacturing processes. Without synthetic textile products, global production of natural fibers would be unable to satisfy global consumer demand for fibers and their products [1,2,3,4,5,6,7]. Total global fiber production has more than doubled since 2000, reaching 132 million tonnes in 2024, with projections of around 169 million tonnes by 2030 under current trends. Synthetic fibers collectively represent 69% of global fiber production [8].

Among synthetic fibers, polyamide and acrylic fibers are widely used owing to their high strength-to-weight ratio, chemical resistance, durability, and cost-effectiveness [1,2,3,4,5,6,7].

Polyamide (PA) fibers, commonly known as nylon fibers, are synthetic fibers characterized by high strength, abrasion resistance, elasticity, durability, and resistance to chemicals and wear. These properties make them suitable for a wide range of textile and industrial applications (Table 1).

Currently available statistics do not provide a global production breakdown by individual polyamide fiber types (e.g., PA6, PA66, PA11, PA12) [8,9]. Instead, authoritative industry sources report total polyamide (nylon) fiber production, which reached approximately 7.0 million tonnes in 2024 (Table 2).

Acrylic fibers contain, by definition, at least 85 wt% acrylonitrile repeating units. Modacrylic fibers contain at least 50 wt% but less than 85 wt% acrylonitrile according to the ISO/BISFA definition, whereas the broader U.S. definition includes fibers containing at least 35 wt% but less than 85 wt% acrylonitrile [10]. In the present review, the term “acrylic fibers” refers exclusively to fibers containing at least 85 wt% acrylonitrile repeating units; modacrylic fibers are considered a separate category. The first modacrylic fiber, Dynel, was produced by Union Carbide in the United States in 1948 [10]. Acrylic fibers are valued for their wool-like appearance, softness, lightweight nature, warmth, good weather resistance, and excellent color retention. These characteristics make acrylic fibers suitable for a wide variety of textile and industrial applications (Table 3).

Unlike polyamide fibers, authoritative public sources do not report annual global production volumes for acrylic fibers as a separate textile fiber category. Instead, Textile Exchange groups acrylic fibers together with polypropylene, elastane, and other synthetic fibers under the category “Other synthetic fibers” [8,9]. The combined production of these fibers reached 6.1 million tonnes in 2023 and 6.3 million tonnes in 2024, while global acrylic fiber production was estimated at around 1.5–1.7 million tonnes in 2024 [8,9].

Textile fibers recovered from a crime scene constitute valuable trace evidence based on Locard’s “every contact leaves a trace” principle. Textile fibers can provide crucial information about physical contact between a victim and a suspect, and they may also establish associations between individuals and a crime scene. In forensic science, fiber examination is primarily based on comparative analysis, in which fibers of unknown origin are compared with reference samples to determine whether they may have originated from the same source. This process requires a high level of expertise because the findings can significantly influence the interpretation of evidence and contribute to determining whether a suspect is linked to a criminal act. Therefore, forensic scientists must conduct thorough and systematic examinations to distinguish between fibers that appear similar but differ in origin or composition. During fiber characterization, all observable physical, chemical, morphological, and optical properties are carefully evaluated to determine the distinctiveness of the sample and its evidential significance. A comprehensive description of these characteristics enhances the reliability and value of the fiber as forensic evidence.

Polyamide and acrylic fibers are frequently encountered as trace evidence in forensic investigations because they transfer readily and retain distinctive microscopic and chemical characteristics [11,12,13,14,15,16,17,18,19]. They are of particular forensic significance because they can (1) link suspects, victims, and crime scenes; (2) associate a fiber with a particular garment, carpet, or upholstery; (3) support the reconstruction of events in homicide, assault, burglary, sexual assault, and hit-and-run investigations; and (4) corroborate other forensic evidence such as DNA, fingerprints, or shoe impressions. Since polyamide and acrylic fibers are widely used, they generally provide class characteristics rather than unique individual identification. However, combining fiber evidence with other trace evidence increases its evidential value [11,12,13,14,15,16,17,18,19]. The most common types of polyamide fibers encountered in forensic investigations are Nylon 6 and Nylon 66 [11].

To make full use of fiber and textile evidence, forensic fiber experts must possess not only specialized forensic expertise but also a thorough understanding of textile materials, including fibers, yarns, and fabrics. Knowledge of their manufacturing processes, chemical and physical structures, and performance characteristics is essential for accurately evaluating the significance of fiber evidence. In addition, experts should be familiar with factors such as production volumes, changes in fashion trends, the introduction of new textile materials, variations in dyes, and other industry developments that may influence the interpretation and evidential value of fiber findings [11,12,13,14,15].

Accurately assessing the similarity between fiber samples requires a comprehensive and systematic analytical approach. The examination typically begins with microscopic analysis of fiber morphology, which serves as an initial screening step [11,12,13,14,15,16,17,18,19,20]. This enables the forensic examiner to evaluate numerous recovered fibers and identify those that warrant more detailed instrumental analysis using such techniques as Fourier transform infrared spectroscopy (FTIR), Raman spectroscopy, differential scanning calorimetry (DSC), thermogravimetric analysis (TGA), or polarized light microscopy (PLM) [11,12,13,14,15,16,17,18,19]. These techniques are employed to determine the polymer composition of the fibers. When the fibers are dyed, microspectrophotometry (MSP), high-performance liquid chromatography (HPLC), thin-layer chromatography (TLC), and UV–visible spectroscopy are commonly used to compare their color characteristics [11,12,13,14,15,16,17,18,19]. However, colorless fibers are sometimes encountered in forensic casework, making color comparison impossible. In such cases, additional analytical techniques are required to compensate for the absence of color-related data, as the lack of complementary color evidence may reduce the overall evidential value of the fiber comparison [21,22].

One of the most authoritative references in the field of forensic fiber analysis is *Forensic Examination of Fibres*, edited by J. Robertson and M. Grieve (2nd edition published in 1999 [13], and the 3rd edition, edited by J. Robertson, C. Roux, and K. G Wiggins, published in 2018 [11]). In addition to this landmark publication, forensic fiber specialists have established several professional organizations to promote standardized methods and best practices. Among these is the European Fibres Group (EFG), operating under the European Network of Forensic Science Institutes (ENFSI), which was renamed the European Textile and Hair Group (ETHG) in 2010. In 2001, the EFG published the *Manual of Best Practice for the Forensic Examination of Fibres*, and a new edition was published in 2022 [23]. The present version, *Best Practice Manual for the Forensic Examination of Fibres*, is based on the official ENFSI template, and can be regarded as a subtle integration of past and present, combining established knowledge with recent needs for standardization and uniformity within the forensic community [23]. Similarly, in the United States, the Scientific Working Group for Materials Analysis (SWGMAT) developed and published the *Forensic Fiber Examination Guidelines* in 1999 [24] to provide standardized procedures for the examination and interpretation of fiber evidence. ASTM International, formerly known as the American Society for Testing and Materials, subsequently published the *Standard Guide for Forensic Analysis of Fibers by Infrared Spectroscopy* [16].

As mentioned, polyamide and acrylic fibers are among the most common synthetic fibers encountered as trace evidence in forensic investigations. Their evidential value depends on maintaining identifiable physical, chemical, and spectroscopic characteristics after exposure to environmental or chemical degradation factors [25,26,27,28,29,30,31,32,33,34,35]. Consequently, understanding degradation mechanisms is essential for interpreting forensic evidence and distinguishing environmental damage from manufacturing characteristics. Despite considerable advances in fiber characterization, differentiating among various forms of damage and determining their causes remains a major challenge in forensic investigations [25,26,27,28,29,30,31,32,33,34,35].

This review examines the degradation mechanisms and physicochemical changes that occur in polyamide and acrylic fibers following exposure to different degrading agents. The existing literature is critically evaluated to explain the processes responsible for fiber deterioration and to relate molecular and structural alterations to changes in their physicochemical and macroscopic properties. Particular emphasis is placed on the effects of environmental, chemical, thermal, and mechanical factors that influence the stability and performance of these fibers. In addition, the review discusses the analytical techniques currently employed to detect and characterize degradation, with consideration of their strengths, limitations, and relevance to forensic examinations. The literature for this review was retrieved from Scopus, an interdisciplinary abstract and citation database. The following search query was applied: (“polyamide fibers” OR “nylon fibers” OR “acrylic fibers” OR “polyacrylonitrile fibers”) AND (“global production” OR “dyeing” OR “degradation mechanisms” OR “physicochemical changes” OR “forensic examination” OR “forensic investigations” OR “forensic analytical techniques” OR “analysis of degraded fibres” OR “environmental degradation” OR “chemical degradation” OR “biological degradation” OR “mechanical degradation” OR “thermal degradation” OR “hydrolytic degradation” OR “photo oxidation” OR “UV-induced changes” OR “color changes”). The following exclusion criteria were applied: “microplastic,” “wastewater,” “water treatment,” “water purification,” and “membrane.” No time range was specified for the search. The search yielded more than 300 publications, which were subsequently screened and analyzed by the authors. In addition, relevant standardized procedures and practical approaches used in the examination and interpretation of fiber evidence were taken into consideration. Based on the available literature, no previous publication has provided such a comprehensive overview of the forensic analysis of degradation processes in both polyamide and acrylic fibers.

## 2. Overview of Fiber Chemistry and Structure

The susceptibility of synthetic textile fibers to environmental, chemical, mechanical, and thermal degradation is fundamentally governed by their chemical composition and structural organization. Understanding how molecular architecture, intermolecular interactions, processing conditions, and supramolecular structure determine the physicochemical properties of polyamide and acrylic fibers is therefore essential for interpreting degradation pathways and the resulting physicochemical changes observed in forensic examinations. For this reason, the following sections first summarize the chemistry, structure, and physicochemical properties of both fiber types before discussing the mechanisms responsible for their degradation. This approach provides the structural framework necessary for understanding how different degrading agents alter fiber morphology, physicochemical characteristics, dye stability, and ultimately the evidential value of textile fibers in forensic investigations.

### 2.1. Polyamide Fibers (Nylon 6 and Nylon 6,6)

#### 2.1.1. Molecular Structure and Polymerization of Polyamide Fibers

**Figure 1:**
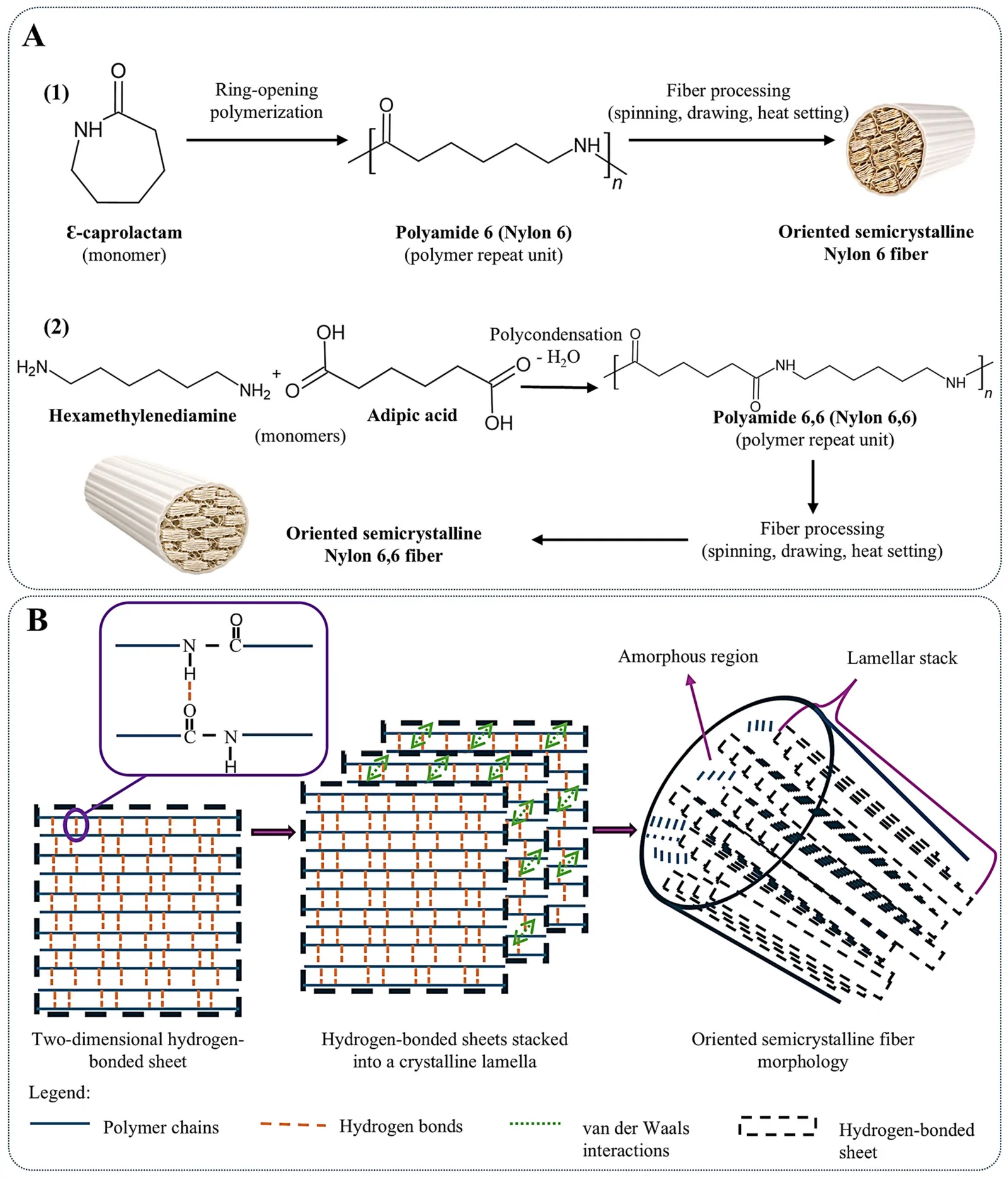
Integrated schematic representation of the synthesis, fiber formation, and hierarchical structural organization of polyamide fibers: (**A**) simplified synthesis and fiber formation of (1) polyamide 6 (nylon 6) through ring-opening polymerization of ε-caprolactam and (2) polyamide 6,6 (nylon 6,6) through polycondensation of hexamethylenediamine and adipic acid, followed by spinning, drawing, and heat setting; and (**B**) hierarchical organization of semicrystalline polyamide fibers, in which polymer chains are interconnected through intermolecular hydrogen bonds to form two-dimensional hydrogen-bonded sheets that are stacked via van der Waals interactions into crystalline lamellae. The coexistence of crystalline lamellar stacks and amorphous regions gives rise to the characteristic semicrystalline fiber morphology.

Polyamides constitute one of the most important classes of synthetic polymers, widely used both as textile fibers and as engineering materials, with Nylon 6 being the most extensively studied member of this family [36,37]. Among commercially available polyamides, Nylon 6 (polyamide 6, PA6, polycaprolactam) and Nylon 6,6 (also referred to as Nylon 66 or polyamide 66, PA66) (Figure 1A) are the most widely used materials owing to their favorable combination of properties and broad applicability in textile production [38,39]. Polyamides are polymers characterized by repeating amide linkages connecting aliphatic chain segments. These amide groups constitute the fundamental structural feature governing the molecular organization and physicochemical behavior of nylon fibers [36].

The commercially dominant route for Nylon 6 production is the ring-opening polymerization of ε-caprolactam, whereas Nylon 6,6 is mainly manufactured by polycondensation of hexamethylenediamine and adipic acid, as schematically illustrated in Figure 1A [39,40,41]. Besides conventional homopolymers, copolymerization has also been employed to tailor the properties of polyamide fibers. For example, copolymerization of Nylon 6, Nylon 4,6, and poly(ethylene oxide)-poly(propylene oxide) (PEO-PPO) diamine has been reported to improve moisture regain while slightly reducing crystalline perfection without altering the fundamental crystalline structure of nylon [42]. Following polymerization, polyamide fibers are manufactured using spinning techniques, during which molecular orientation develops and contributes to the formation of well-defined supramolecular fiber structures [37,38].

**Figure 2:**
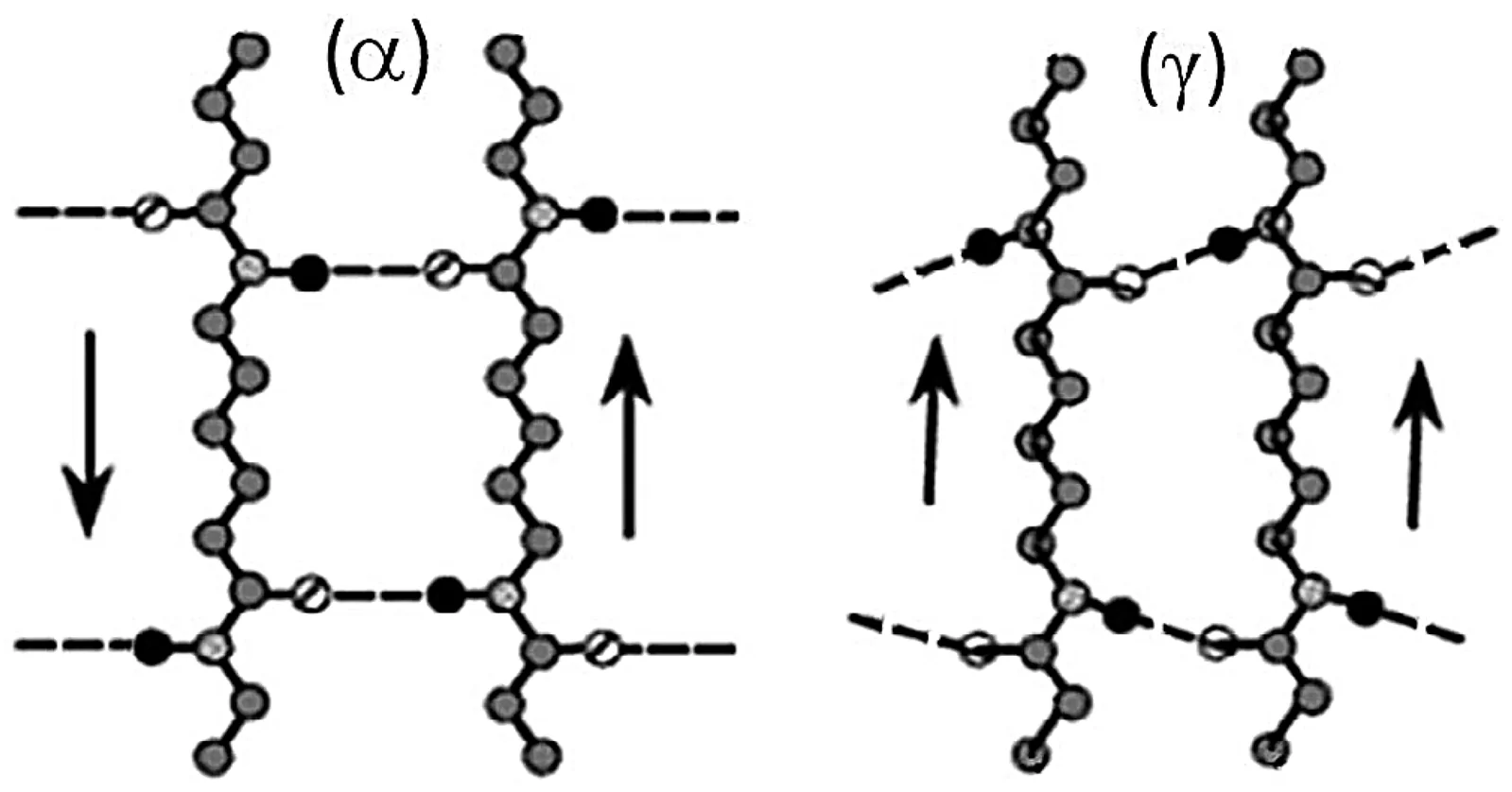
Schematic representation of hydrogen-bonding arrangements in the α- and γ-crystalline polymorphs of PA6. Reproduced from Seguela, R. “Overview and Critical Survey of Polyamide6 Structural Habits: Misconceptions and Controversies,” *Journal of Polymer Science* 2020, 58, 2971–3003 [37], with permission from John Wiley and Sons. © 2020 Wiley Periodicals LLC.

The characteristic properties of polyamide fibers arise primarily from the presence of amide groups regularly distributed along the polymer backbone [36,43]. These functional groups establish extensive intermolecular hydrogen bonds between neighboring polymer chains, giving rise to well-defined hydrogen-bonded sheets that constitute the structural basis of nylon crystals and largely determine their physicochemical properties [36,37]. In PA6, the hydrogen-bonded sheets can adopt different packing arrangements, resulting in the α and γ crystalline polymorphs of polyamides (Figure 2), which are discussed in more detail in the following section. Compared with common thermoplastics such as polyolefins or polyesters, which rely predominantly on weaker intermolecular interactions, aliphatic polyamides exhibit substantially higher molecular cohesion due to their extensive hydrogen-bonding network [37].

Nearly all amide groups separating the methylene sequences in aliphatic nylons participate in hydrogen bonding, making these interactions a fundamental structural feature of polyamide fibers [36]. Besides increasing intermolecular cohesion, hydrogen bonds restrict chain mobility in the molten state by limiting long-range molecular rearrangements and preserving chain entanglements during processing [37]. Consequently, hydrogen bonding plays a crucial role in the molecular mobility, hydration, chain folding, crystallization, and deformation behavior of nylon fibers [36]. Variations in the arrangement of interchain hydrogen bonds are also responsible for the polymorphic behavior of PA6, determining the packing of polymer chains into different crystalline structures [43].

The density of amide groups directly influences the extent of hydrogen bonding and, thus, the structural organization of polyamides. A lower concentration of amide groups results in weaker intermolecular interactions, leading to reduced crystallinity, lower melting temperatures, and deterioration of mechanical properties [40]. Therefore, the hydrogen-bonding network represents one of the principal factors governing the molecular organization and physicochemical performance of polyamide fibers [36,37].

#### 2.1.2. Crystallinity and Morphology of Polyamide Fibers

Polyamide fibers are semicrystalline polymers whose structural organization is governed by molecular regularity, intermolecular hydrogen bonding, and processing conditions [37]. Interchain hydrogen bonds impose topological constraints that restrict chain rearrangement during crystallization, making the final degree of crystallinity strongly dependent on factors that promote chain mobility, molecular orientation, and crystal nucleation [37].

The crystalline structure of nylon is based on the organization of polymer chains into well-defined two-dimensional hydrogen-bonded sheets that are interconnected through van der Waals interactions to form a three-dimensional crystal lattice (Figure 1B) [36]. This hierarchical organization is retained in the semicrystalline state, in which ordered crystalline domains coexist with amorphous regions interconnected by entangled polymer chains [37]. Molecular orientation induced during spinning enhances crystallization by restricting chain relaxation and promoting chain unfolding into more extended conformations, thereby increasing the crystallization potential of polyamide fibers [37]. During spinning, this orientation results in a more uniform fiber structure than that observed in corresponding polymer films [42]. Subsequent drawing further increases molecular orientation and crystallinity, thereby improving the structural organization of semicrystalline polyamide fibers [44]. Post-drawing and heat-setting processes additionally stabilize the fiber microstructure by relaxing frozen-in stresses and promoting structural reorganization at elevated temperatures [41]. The extent of molecular orientation ultimately depends on processing conditions, particularly spin-line stress, molecular weight, quenching conditions, and draw ratio [41].

**Figure 3:**
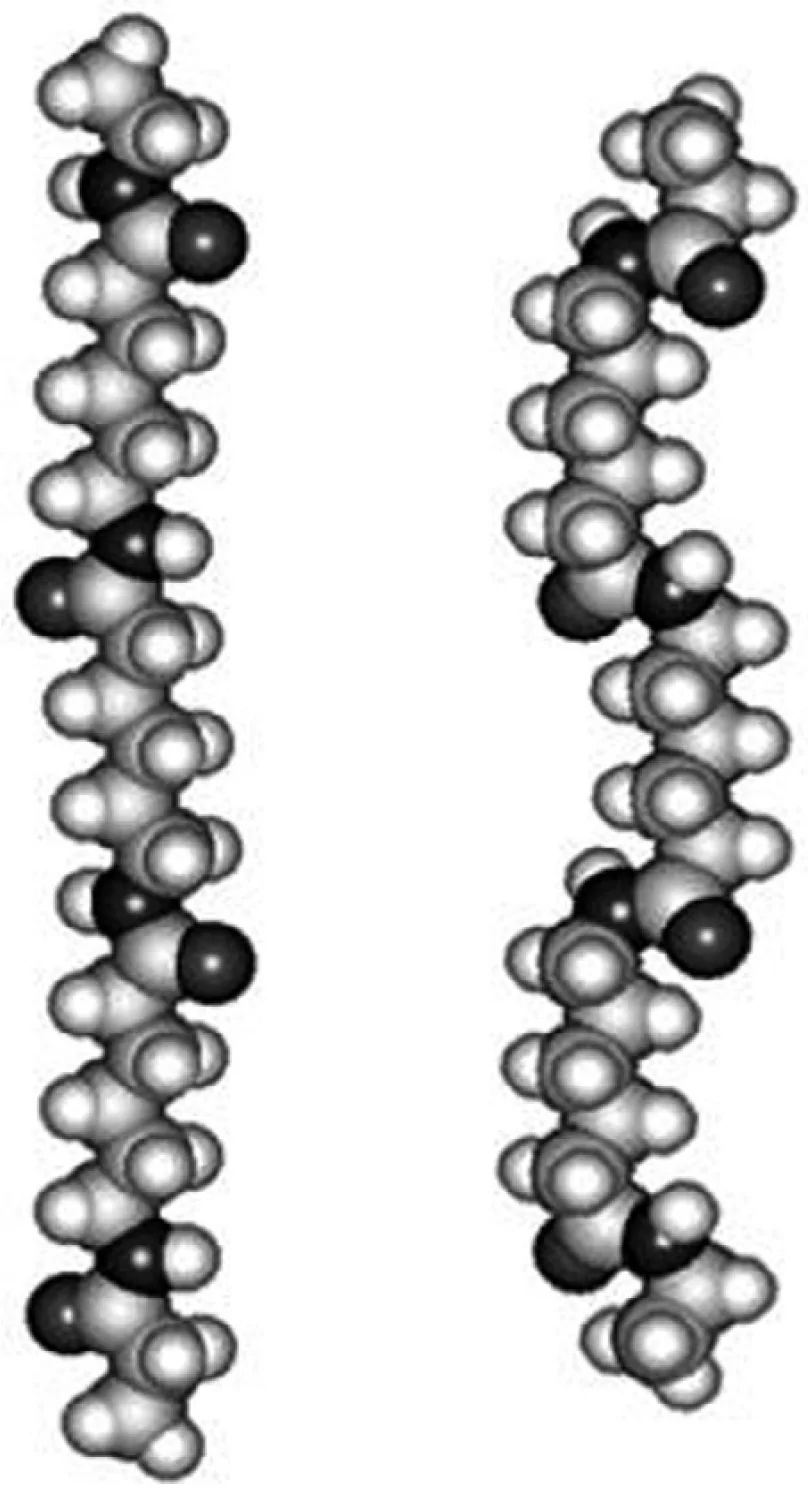
Molecular chain conformations of PA6 in the α- and γ-crystalline forms: fully extended all-trans conformation of the α-form and skewed, chain-twisted conformation of the γ-form. Reproduced from Seguela, R. “Overview and Critical Survey of Polyamide6 Structural Habits: Misconceptions and Controversies,” *Journal of Polymer Science* 2020, 58, 2971–3003 [37], with permission from John Wiley and Sons. © 2020 Wiley Periodicals LLC.

Polyamide 6 exhibits polymorphism and may crystallize in three distinct crystalline modifications, namely the α-, β-, and γ-phases [37,45]. Melt-spun PA6 fibers are predominantly characterized by the metastable γ phase, whereas subsequent drawing promotes the γ-to-α transformation, resulting in a thermodynamically more stable crystalline structure and higher crystallinity [44]. Compared with PA6, PA66 exhibits a higher density of intermolecular hydrogen bonding, which promotes faster crystallization, higher crystal perfection, and a higher melting temperature [46]. As schematically illustrated in Figure 2, the arrangement of hydrogen-bonded polymer chains differs between the α- and γ-crystalline polymorphs. In the α-phase, hydrogen bonds are formed between antiparallel polymer chains adopting a fully extended planar zigzag conformation, whereas the γ-phase is characterized by hydrogen bonding between parallel chains associated with a pleated-sheet (gauche) conformation [36,46,47]. Representative molecular chain conformations corresponding to these two polymorphs are shown in Figure 3. The transformation from the γ- to the α-phase involves local conformational changes accompanied by a cooperative switching of hydrogen bonds [37].

The crystalline morphology of PA6 is strongly influenced by crystallization and processing conditions, which determine the relative abundance of different crystalline modifications [37]. Slow cooling or isothermal crystallization generally favors the formation of the α-phase, whereas rapid cooling, drawing, or spinning promotes the γ-phase [43]. Recent calorimetric and spectroscopic studies demonstrated that the β-phase predominates below 105 °C, whereas the γ-phase coexists with either the β- or α-phase between 105 and 150 °C. Above 150 °C, crystallization proceeds predominantly through the formation of the thermodynamically stable α-phase [45]. Unlike the well-defined α- and γ-crystalline forms, the β-phase is generally considered a mesomorphic, partially ordered structure that develops under rapid crystallization conditions and may subsequently transform into more stable crystalline modifications [45]. Similarly, molecular orientation that develops during spinning influences the crystalline structure. The γ-form predominates at lower take-up speeds, whereas increasing take-up speed promotes the formation of the thermodynamically more stable α-form [39].

Besides the crystalline domains, Nylon 6 fibers also contain amorphous regions composed of both isotropic and anisotropic components [39]. Several post-processing and crystallization treatments, including pressure-assisted crystallization, electrospinning, iodine treatment, superheated water treatment, and crystallization from dilute solutions, have been shown to enhance crystallinity by promoting molecular orientation, increasing chain mobility through the temporary disruption of hydrogen bonds, or facilitating crystal nucleation [37]. Morphological observations revealed a transition from crystal aggregates formed at low crystallization temperatures to well-developed lamellar and spherulitic structures at higher crystallization temperatures, reflecting the progressive evolution of crystalline organization during crystallization [45].

#### 2.1.3. Physicochemical Properties of Polyamide Fibers

The unique physicochemical properties of polyamide fibers arise from the interplay between molecular architecture, intermolecular hydrogen bonding, crystalline organization, and molecular orientation [36,40]. Hydrogen bonding remains the principal structural factor governing the physicochemical behavior of polyamide fibers, largely through its influence on intermolecular cohesion and crystalline organization [36]. Within crystalline regions, extensive amide–amide hydrogen bonding contributes substantially to the excellent mechanical properties of polyamides by reinforcing intermolecular cohesion and stabilizing the crystalline structure [36].

The density of amide groups represents another important structural parameter controlling fiber performance. As discussed above, variations in amide-group density influence intermolecular hydrogen bonding and consequently affect the properties of polyamides. A lower amide-group density is generally associated with reduced moisture absorption and improved chemical resistance owing to the decreased polarity of the polymer chains. These observations demonstrate the close relationship between chemical composition, supramolecular organization, and the resulting physicochemical behavior of polyamide fibers [40].

Among commercially important polyamides, Nylon 6 is characterized by high tensile strength, excellent elasticity, elongation, and resilience, as well as relatively good hygroscopicity, dyeability, and chemical resistance [42]. These properties are closely associated with the coexistence of crystalline and amorphous phases and the hydrogen-bonding network within the fiber structure [39,42]. Processing conditions further influence fiber performance by modifying macromolecular orientation, crystallinity, and the relative abundance of the α- and γ-crystalline forms [37,39]. These structural changes translate directly into improvements in the mechanical properties of PA6 fibers [44].

Overall, the physicochemical properties of polyamide fibers are governed by the interplay between molecular structure, hydrogen bonding, crystallinity, crystal polymorphism, molecular orientation, and higher-order structural features, including lamellar organization, crystal size, the rigid amorphous fraction, and the distribution of crystalline and amorphous regions [36,40,46]. Consequently, higher molecular orientation and crystallinity, promoted by spinning, drawing, and heat-setting, generally result in improved tensile strength and elastic modulus of polyamide fibers [44,46]. Experimental studies have further demonstrated a direct correlation between crystalline organization and the elastic modulus of PA6 fibers, confirming the strong influence of crystalline structure on their mechanical performance [44]. Furthermore, extensive intermolecular hydrogen bonding promotes dense chain packing and high crystallinity, thereby limiting the accessibility of amide bonds to water, enzymes, and other external agents, and increasing the resistance of polyamides to chemical and enzymatic degradation [48].

### 2.2. Acrylic Fibers (Polyacrylonitrile-Based Fibers)

#### 2.2.1. Molecular Structure and Polymerization of Acrylic Fibers

**Figure 4:**
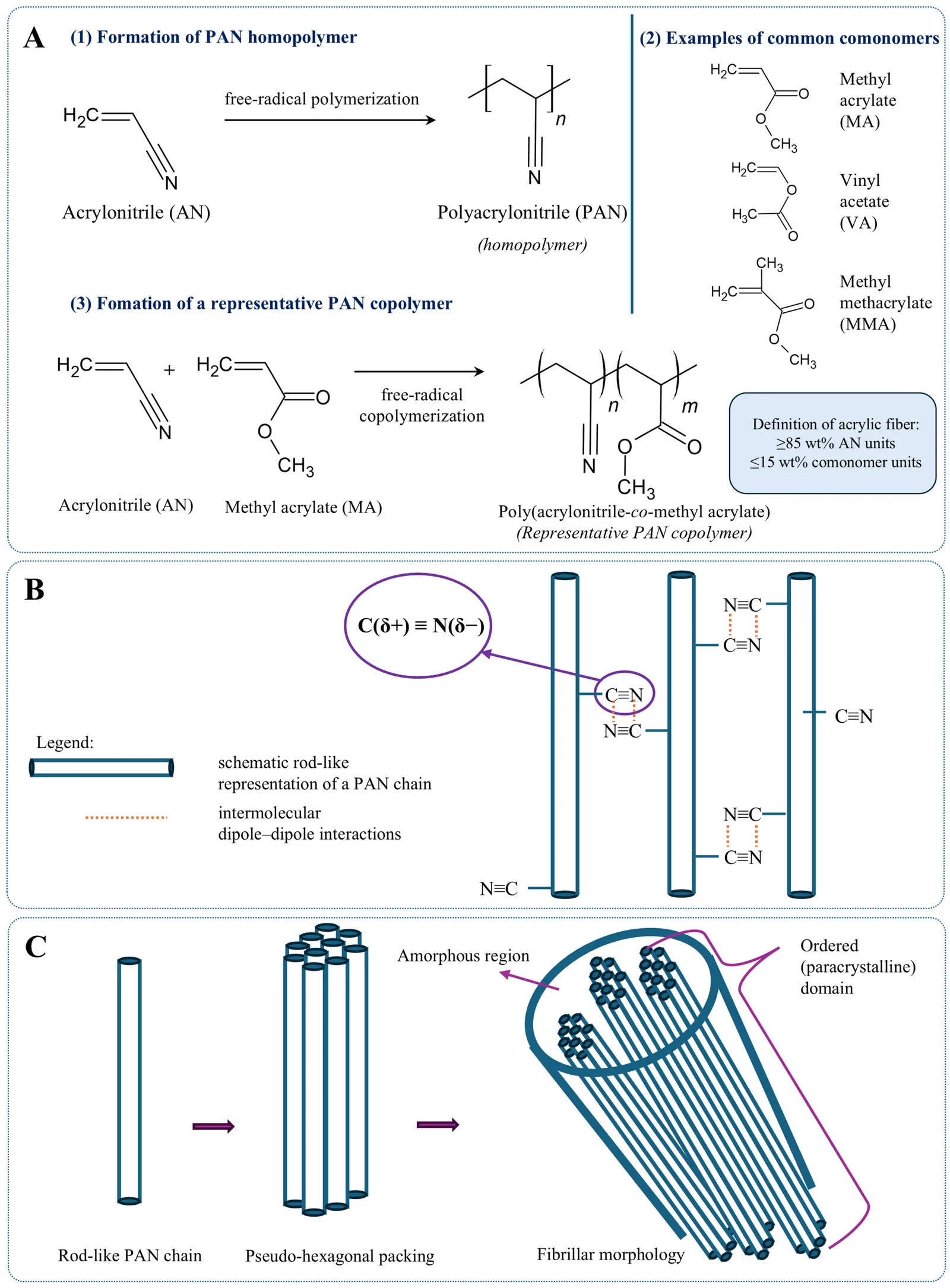
Integrated schematic representation of the molecular and hierarchical structure of acrylic fibers: (**A**) (**1**) free-radical polymerization of acrylonitrile to form PAN homopolymer, (**2**) representative comonomers used in PAN-based copolymers, and (**3**) free-radical copolymerization of acrylonitrile with methyl acrylate to form a representative PAN-based copolymer; commercial acrylic fibers contain at least 85 wt% acrylonitrile repeating units, while the remaining fraction may consist of one or more comonomers; (**B**) intermolecular dipole–dipole interactions between nitrile groups on neighboring PAN chains, which promote lateral chain packing; and (**C**) hierarchical organization of acrylic fibers, in which relatively rigid, rod-like PAN chains undergo pseudo-hexagonal lateral packing to form paracrystalline domains within less ordered amorphous regions, collectively contributing to the characteristic fibrillar morphology of commercial acrylic fibers.

Acrylic fibers constitute one of the major classes of synthetic textile fibers. They are composed predominantly of polyacrylonitrile (PAN) and, consistent with the definition provided above, contain at least 85 wt% acrylonitrile repeating units, while the remaining fraction may consist of one or more comonomers introduced to modify fiber properties (Figure 4A(1,3)) [49,50]. Commercial acrylic fibers are generally produced as PAN-based copolymers rather than as PAN homopolymers. The incorporation of comonomers improves processability, dyeability, and chain mobility without fundamentally altering the polyacrylonitrile backbone [49,51]. Common examples of neutral comonomers include methyl acrylate (MA), methyl methacrylate (MMA), and vinyl acetate (VA) (Figure 4A(2)) [52], whereas itaconic acid (IA) and acrylamide (AM) are frequently incorporated as functional comonomers to tailor polymer properties [51]. Neutral comonomers facilitate segmental mobility and dye diffusion, whereas ionic comonomers introduce additional dyeing sites for cationic dyes, thereby enhancing the physicochemical properties and dyeability of acrylic fibers [49,52].

Polyacrylonitrile is typically synthesized via free-radical polymerization of acrylonitrile. Although alternative polymerization approaches, such as anionic photopolymerization, have also been investigated, free-radical polymerization or copolymerization remains the predominant route for the production of commercial acrylic fibers [53]. The type and reactivity of incorporated comonomers influence the stability of the copolymerization system and thereby affect the structure and physicochemical properties of the resulting PAN copolymers [51]. Following polymerization, the PAN-based polymer is converted into fibers by either wet spinning or dry spinning, which remain the two principal manufacturing techniques for commercial acrylic fibers [50,54]. In wet spinning, the polymer solution is extruded into a coagulation bath, where the solvent is removed, whereas in dry spinning, the solvent evaporates during fiber formation [50,54]. The selected spinning route significantly influences the subsequent structural organization of the fibers [55].

The molecular structure of polyacrylonitrile is characterized by the presence of one highly polar nitrile (–C≡N) group in each repeating unit of the polymer backbone. These groups generate strong intermolecular dipole–dipole interactions (Figure 4B) that promote close chain packing and represent the principal structural feature governing the organization of acrylic fibers. Unlike polyamides, whose molecular organization is governed primarily by extensive hydrogen bonding, acrylic fibers therefore rely predominantly on dipole–dipole interactions for structural cohesion [54]. Molecular characteristics, including molecular weight and copolymer composition, are closely related to the processing behavior and final performance of acrylic fibers [56].

**Figure 5:**
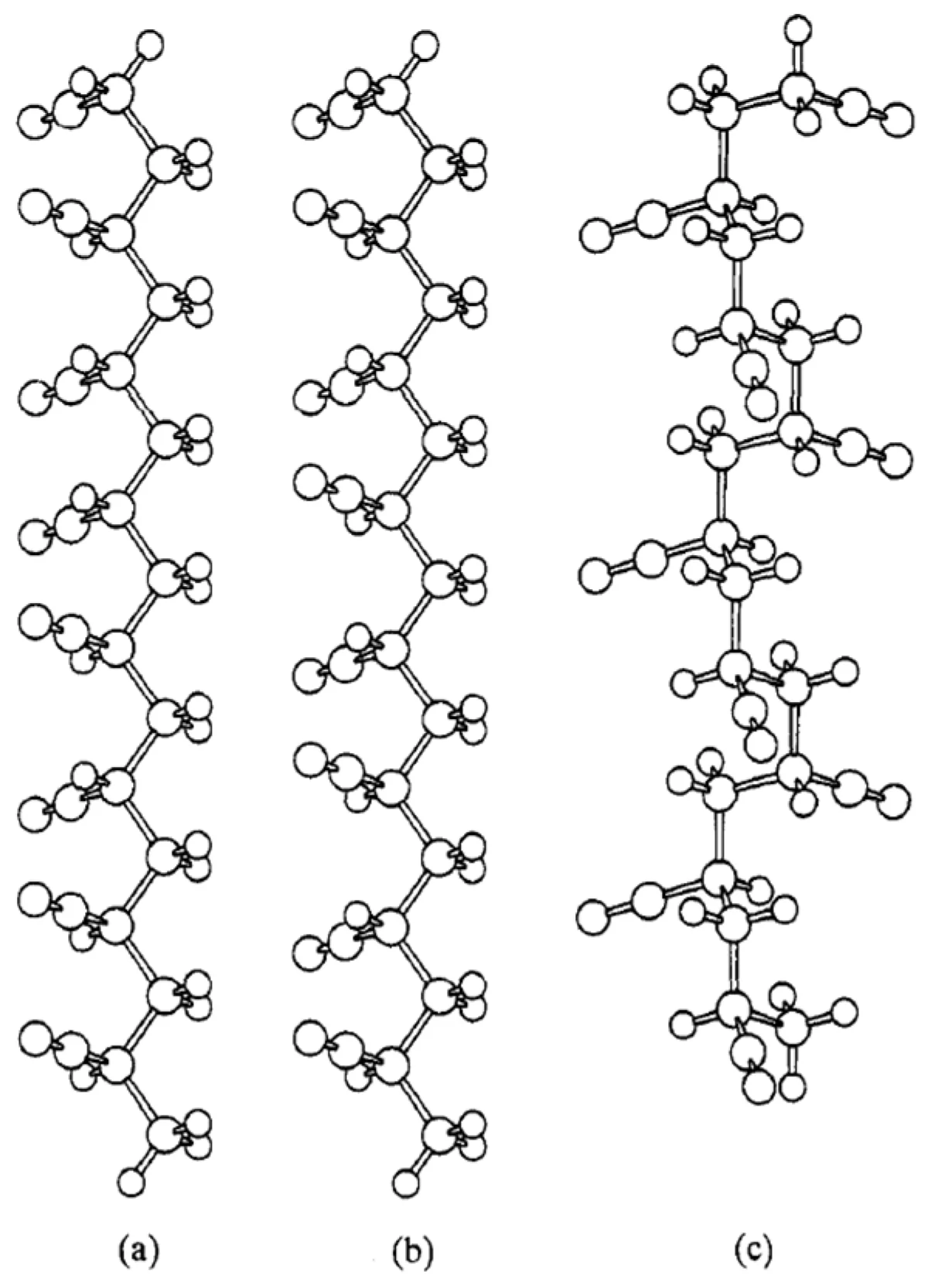
Molecular models of PAN chain conformations: (**a**) atactic planar-zigzag, (**b**) syndiotactic planar-zigzag, and (**c**) isotactic helical conformations. Reprinted with permission from Liu, X.D.; Ruland, W. X-ray studies on the structure of polyacrylonitrile fibers. *Macromolecules* 1993, 26, 3030–3036 [57]. Copyright 1993 American Chemical Society.

The technical PAN homopolymer fibers investigated by Liu and Ruland exhibited predominantly atactic stereochemistry, characterized by a statistically random distribution of nitrile-group orientations along a planar-zigzag polymer backbone [57]. This atactic configuration does not prevent the development of a considerable degree of molecular ordering during spinning and drawing, giving rise to the supramolecular organization characteristic of commercial acrylic fibers [58]. To facilitate structural interpretation, Figure 5 compares the principal chain conformations considered for PAN. The investigated PAN homopolymer fibers are best described by an atactic planar-zigzag backbone (Figure 5a), whereas syndiotactic planar-zigzag (Figure 5b) and isotactic helical conformations (Figure 5c) represent idealized stereochemical models used primarily for comparison in structural analyses. X-ray diffraction studies indicate that the planar-zigzag conformation provides the best agreement with the experimental structure of the investigated PAN homopolymer fibers, whereas the isotactic helical model does not adequately describe their structure [57]. The syndiotactic planar-zigzag model is mainly employed as a reference for comparison during structural interpretation rather than as a representation of commercial fibers [57]. The relatively rigid and elongated geometry of the atactic planar-zigzag chains provides the structural basis for representing PAN molecules as rod-like units in supramolecular models of acrylic fibers. These rod-like chains subsequently undergo lateral packing during fiber formation, giving rise to the higher-order structural organization discussed in the following section [1]. Consequently, the molecular composition of acrylic fibers represents a balance between preserving the favorable mechanical properties associated with the PAN backbone and introducing sufficient functionality to improve processability, thermal behavior, and dyeability [49,50,51].

The molecular structure and copolymer chemistry of acrylic fibers establish the basis for their subsequent supramolecular organization and physicochemical behavior. The influence of molecular ordering and processing on the crystalline structure, morphology, and physicochemical properties of acrylic fibers is discussed below.

#### 2.2.2. Crystallinity and Morphology of Acrylic Fibers

Acrylic fibers develop varying degrees of molecular ordering during processing, giving rise to ordered (paracrystalline) and less ordered (amorphous) structural regions [49,54]. As introduced in the previous section, the relatively rigid and elongated geometry of PAN chains enables their representation as rod-like structural units. Owing to strong but relatively non-directional dipole–dipole interactions between neighboring nitrile groups, these rod-like chains undergo lateral packing, producing a pseudo-hexagonal, pseudocrystalline arrangement that constitutes the basis of the higher-order structural organization of acrylic fibers (Figure 4C) [1,58]. The high polarity of nitrile groups restricts segmental mobility even within the less ordered regions of the polymer, contributing to the high degree of molecular ordering characteristic of polyacrylonitrile [49]. Consequently, this highly ordered supramolecular organization may reduce the accessibility of dye molecules to the fiber interior. For this reason, suitable comonomers are incorporated into commercial acrylic fibers to increase segmental mobility and facilitate dye diffusion [49].

The degree of molecular ordering, molecular orientation, and supramolecular organization is strongly governed by the spinning process and subsequent drawing operations, making the final fiber morphology highly dependent on processing conditions [49,54,59].

Unlike many synthetic fibers, acrylic fibers do not exhibit a well-defined two-phase fine structure. Instead, their microstructure is generally described as fibrillar, comprising elongated fibrils with relatively strong cohesion between adjacent structural units [54]. This fibrillar morphology develops during spinning and drawing and plays an important role in determining the mechanical behavior and failure characteristics of acrylic fibers [54]. Recent microscopic investigations have further demonstrated that post-spinning treatments promote the gradual transformation of a loose microfibrillar network into a highly oriented fibrillar structure, accompanied by a more uniform microstructural organization under optimized processing conditions [60].

During spinning, stretching, and drying, polymer chains become progressively oriented along the fiber axis, resulting in a high degree of molecular ordering [49]. Experimental studies on wet-spun PAN-based precursor fibers have shown that molecular orientation increases progressively throughout successive drawing stages, whereas crystallite growth becomes particularly pronounced during hot drawing [59]. Similar observations have been reported for both wet-spinning and dry-jet wet-spinning, where post-spinning treatments promote progressive improvements in crystal arrangement, fibril orientation, and overall microstructural organization, particularly during the drying densification and steam-stretching stages [60].

The pseudo-hexagonal packing of rod-like PAN chains is highly sensitive to processing conditions and external physicochemical factors, which may alter intermolecular packing and the resulting supramolecular organization of acrylic fibers [1,54]. Under specific processing conditions, water molecules may interact with nitrile groups through hydrogen-bonding interactions, temporarily modifying the intermolecular packing and inducing a reversible transformation from the conventional pseudo-hexagonal arrangement to a hydrated orthorhombic structure [61]. Upon drawing and progressive water removal, the hydrated structure reverts to the conventional pseudo-hexagonal packing, illustrating the sensitivity of PAN supramolecular organization to changes in processing conditions and intermolecular interactions [61]. Wide-angle X-ray diffraction studies have likewise demonstrated that the paracrystalline regions of PAN undergo structural rearrangements under swelling conditions, further confirming the sensitivity of acrylic-fiber morphology to external physicochemical stimuli [62].

Consequently, the morphology of acrylic fibers is governed by the combined effects of molecular ordering, chain orientation, fibrillar organization, and processing history, which collectively determine their final supramolecular structure [49,54].

#### 2.2.3. Physicochemical Properties of Acrylic Fibers

The physicochemical properties of acrylic fibers are governed by the close relationship between molecular structure, condensed structure, intermolecular interactions, and processing history [1,56]. As discussed in the previous sections, the combination of PAN molecular architecture, supramolecular organization, and processing-induced molecular orientation determines the final mechanical, thermal, and chemical performance of acrylic fibers [1,54,56]. Consequently, variations in molecular weight, copolymer composition, molecular ordering, and fiber orientation directly influence the performance of commercial acrylic fibers [54,56].

Among commercially available synthetic fibers, acrylic fibers are characterized by good tensile properties, wool-like elasticity and elastic recovery, chemical resistance, heat resistance, and good solution processability [54,56,63]. These properties arise from the structural features described above and are further modified by the molecular composition of the copolymer [54,56]. The excellent chemical resistance of acrylic fibers has been attributed to their laterally bonded structure, in which strong intermolecular cohesion must be disrupted before substantial chemical attack, melting, or solvation can occur [63]. The incorporation of suitable comonomers improves processability and dyeability and has also been reported to influence the thermal stability of PAN copolymers [50,51]. In the examined PAN copolymer systems, itaconic-acid-containing copolymers have been reported to show improved thermal stability during thermal treatment [51]. Recent studies have further confirmed that improvements in crystallinity, crystal size, orientation index, and fibrillar alignment are accompanied by enhanced tensile strength and modulus of PAN-based copolymer precursor fibers under specific processing conditions [60].

The thermomechanical behavior of acrylic fibers is temperature-dependent. Increasing temperature results in a progressive reduction in tensile modulus and resistance to extension while also affecting the elastic recovery of the fibers owing to increased molecular mobility within the polymer structure [1].

The physicochemical behavior of acrylic fibers is also strongly influenced by their interaction with water. Water acts as an important hydrogen-bonding partner for PAN-based polymers and influences segmental mobility, moisture transport, and dye diffusion within acrylic fibers [49]. Rather than being governed primarily by water retention, the moisture response of acrylic fibers has been attributed to efficient water transport within the fiber structure [49]. Commercial acrylic fibers generally exhibit a moisture regain of about 2%, although hydrophilic comonomers and modifications of the fiber microstructure may increase water uptake for specific applications [63].

Dyeability represents another important physicochemical characteristic of acrylic fibers. PAN homopolymer exhibits limited affinity toward dyes; therefore, commercial acrylic fibers are generally produced as copolymers containing functional comonomers that introduce negatively charged sites into the polymer structure [50]. These anionic groups readily form ionic interactions with cationic dyes, substantially improving dye uptake while maintaining good colorfastness during laundering [50].

## 3. Dyes Applied in Polyamide and Acrylic Dyeing

The selection of dyes for textile fibers is governed primarily by the chemical structure of the polymer substrate and the intermolecular interactions established between dye molecules and the fiber. Owing to their distinct chemical compositions, polyamide and acrylic fibers are generally dyed using different dye classes and fixation mechanisms [50,64]. Understanding these interactions is essential for interpreting the stability of dyed fibers and their response to environmental and chemical degradation, thereby providing the basis for the forensic interpretation of textile fiber evidence. The following sections discuss these mechanisms separately for polyamide and acrylic fibers.

### 3.1. Polyamide Dyeing Methodology

**Figure 6:**
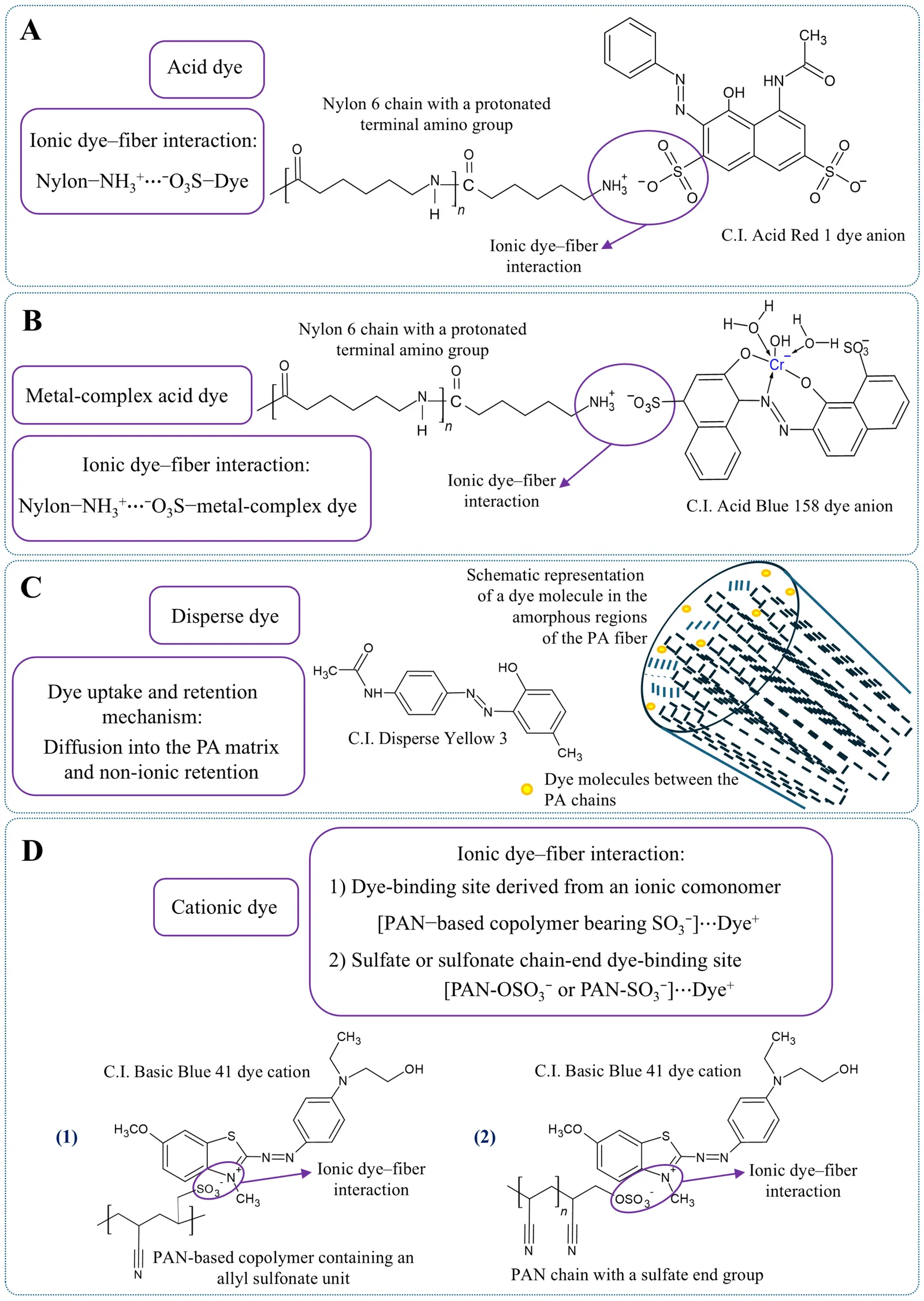
Representative dye–fiber interactions and dye-retention mechanisms in polyamide (**A**–**C**) and acrylic fibers (**D**): (**A**) ionic interaction of the C.I. Acid Red 1 dye anion with a protonated terminal amino group of nylon 6; (**B**) ionic interaction of the metal-complex C.I. Acid Blue 158 dye anion with a protonated terminal amino group of nylon 6; (**C**) diffusion of a disperse dye into accessible amorphous regions of the polyamide matrix and its retention through non-ionic interactions; and (**D**) ionic interaction of the C.I. Basic Blue 41 dye cation with anionic dye-binding sites in acrylic fibers arising from (**1**) an allyl sulfonate comonomer unit and (**2**) a sulfate chain-end group derived from sulfate-radical initiation. One dye-binding site is shown in panels (**A**,**B**); counterions and additional non-ionic interactions are omitted where applicable.

The chemical structure of polyamide fibers determines both the classes of dyes that can be applied and the intermolecular interactions responsible for dye fixation. Among the various dye classes available for textile coloration, acid dyes are the most widely used for polyamide fibers owing to their high affinity for terminal amino groups, which become protonated under weakly acidic conditions and form cationic binding sites for anionic dye molecules (Figure 6A) [50,64,65].

Metal-complex acid dyes (Figure 6B) may also be employed when improved wet and light fastness is required, whereas disperse dyes may be used for selected polyamide dyeing applications [64]. Unlike acid dyes, disperse dyes do not bind to protonated amino end groups but are retained within the polyamide matrix through a non-ionic solution mechanism (Figure 6C) [64].

The dyeing of polyamide fibers with acid dyes is governed primarily by terminal amino groups, which become protonated under acidic conditions and form cationic sites capable of binding anionic dye molecules [50,64,66]. However, dye fixation cannot be explained solely by ionic attraction. Lewis classified dye–fiber interactions into several categories, including electrostatic, van der Waals, induction, charge-transfer, and solvophobic interactions. In the dyeing of polyamide fibers with anionic dyes, electrostatic attraction is supplemented particularly by solvophobic or hydrophobic interactions, whereas induction and charge-transfer contributions are considered comparatively weak [66].

The effectiveness of dye uptake is closely related to the physicochemical characteristics of polyamide fibers. As discussed in the previous section, molecular orientation, crystallinity, and supramolecular organization influence the accessibility of dye molecules to the polymer matrix. Consequently, the structural organization developed during fiber processing plays an important role in determining dye diffusion and the final distribution of color within the fiber.

Accordingly, degradation of a dyed polyamide fiber may involve changes in the polymer matrix, the dye molecules, or the interactions between them. Such changes may weaken dye–fiber interactions and contribute to dye migration, fading, or alterations in color intensity and analytical response. Distinguishing among these contributions is therefore important for the forensic interpretation of exposure-induced changes, as discussed in the following section.

### 3.2. Acrylic Dyeing Methodology

Unlike polyamide fibers, acrylic fibers exhibit a markedly different dyeing behavior resulting from their chemical composition. Commercial acrylic fibers are generally produced from PAN-based copolymers containing both neutral and ionic comonomers that improve processability and dyeability. Neutral comonomers, including methyl acrylate and vinyl acetate, facilitate dye diffusion within the fiber, whereas ionic comonomers introduce negatively charged sites required for the adsorption of cationic dyes [52]. When the number of available dyeing sites is insufficient, sulfonate-containing comonomers may be incorporated to provide additional binding sites for cationic dyes [52]. In addition to ionic comonomers, sulfate or sulfonate end groups introduced during redox initiation and free-radical termination of acrylonitrile copolymerization may also provide anionic dyeing sites for cationic dyes [52,67,68,69] (Figure 6D).

The dyeability of acrylic fibers is influenced by the internal mobility of the polymer segments. Water plasticizes polyacrylonitrile and lowers its glass-transition temperature, thereby increasing segmental mobility and facilitating dye diffusion within the fiber [49]. In addition, the degree of molecular ordering developed during spinning, stretching, and drying influences the accessibility of dye molecules to the fiber interior, making supramolecular organization an important factor governing dye uptake [49].

The interaction between cationic dyes and acrylic fibers is dominated by electrostatic attraction between positively charged dye molecules and negatively charged sites present in the PAN-based copolymer, including ionic comonomer units and sulfate or sulfonate end groups [52,67]. The ionic interactions established between cationic dyes and anionic sites in acrylic fibers are responsible for dye fixation [67]. However, environmental or chemical degradation may gradually modify the polymer structure, the dye molecules, or the dye–fiber interactions responsible for dye fixation, thereby affecting color stability during aging.

## 4. Classification of Degrading Agents

Textile fibers recovered as forensic evidence are rarely encountered in their original, pristine condition because they are routinely exposed to environmental, chemical, mechanical, and thermal stress before collection [50]. Forensic examinations are therefore typically performed on fibers that have already undergone varying degrees of physicochemical alteration. The observed physicochemical characteristics reflect the cumulative effects of manufacturing, service conditions, environmental exposure, and aging throughout the lifetime of the textile [34].

Although textile fibers are generally regarded as class evidence, modern analytical techniques capable of characterizing polymer degradation and dye composition at the molecular level have substantially increased their discriminatory value, allowing differentiation between visually similar fibers that cannot be distinguished by conventional microscopic or spectroscopic methods alone [50,70]. The evidential value of textile fibers further depends on factors such as environmental exposure, transfer and persistence, the interval between the incident and evidence collection, and the analytical methods applied during forensic examination [71]. In addition, the examination of textile damage patterns may provide valuable information regarding the mechanism of damage and assist in reconstructing the sequence of events associated with a criminal incident [72]. Consequently, understanding the mechanisms responsible for fiber and dye degradation is essential for the correct interpretation of forensic evidence, as degradation may alter both the polymer structure and the dyes, potentially affecting both fiber comparison and evidential value.

The following sections examine each category of degrading agents and the corresponding degradation mechanisms in greater detail.

### 4.1. Environmental Factors (UV Radiation, Temperature, Humidity)

Environmental exposure represents one of the most common causes of textile aging throughout the service life of polymeric fibers. Environmental degradation results from the combined action of ultraviolet radiation, temperature, atmospheric oxygen, and humidity (collectively termed weathering), together with surface soiling, while the extent of degradation depends on both the chemical composition and the supramolecular structure of the fiber [34,50]. These factors rarely act independently; instead, they usually operate simultaneously and often accelerate one another. Depending on the degrading agent, physicochemical changes may occur within the polymer matrix, the dye molecules, or both, resulting in modifications of fiber morphology, color, and analytical characteristics [50].

Ultraviolet radiation is regarded as one of the most important degradation factors affecting synthetic textile fibers. Upon UV irradiation, photooxidative reactions induce polymer chain scission, oxidation, discoloration, and the gradual deterioration of mechanical properties [50]. Photodegradation frequently becomes visible as fading or discoloration before severe deterioration of the polymer structure is observed [50]. Experimental studies have further demonstrated that environmental degradation may significantly modify the optical characteristics of dyed fibers, potentially increasing the risk of false exclusions during forensic comparison when the exposure history of the evidence is not considered [73].

Temperature also plays a critical role in the aging behavior of textile fibers. Elevated temperatures accelerate oxidation reactions, increase molecular mobility, and may promote thermal degradation of both polymers and dyes [50]. When combined with solar radiation, heat substantially enhances the rate of weathering processes, resulting in cumulative degradation effects that exceed those produced by individual factors alone [50].

Humidity further contributes to degradation by facilitating moisture-induced physicochemical changes and modifying intermolecular interactions within susceptible polymer fibers [50]. In addition, moisture may promote structural relaxation, thereby influencing polymer mobility and dye diffusion, particularly in acrylic fibers [49]. Fluctuating humidity may also induce repeated swelling and contraction of polymeric fibers, increasing their susceptibility to both chemical degradation and mechanical damage during prolonged exposure [34]. Consequently, the combined action of UV radiation, temperature, humidity, and atmospheric oxygen constitutes the principal environmental aging mechanism affecting dyed polyamide and acrylic fibers encountered in forensic investigations.

### 4.2. Chemical Agents (Acids, Bases, Oxidizing Agents)

Chemical degradation results from the exposure of textile fibers to reactive substances capable of altering their physicochemical characteristics. In forensic casework, characteristic patterns of chemically induced textile damage may provide valuable information regarding the mechanism responsible for the damage and assist in the reconstruction of criminal events [72]. Chemical degradation may occur through several mechanisms, the extent of which depends on the chemical structure of the polymer and the exposure conditions [34].

Acidic and alkaline environments may accelerate hydrolysis or other degradation reactions depending on the chemical structure of the polymer [34]. Under strongly acidic conditions, polyamide fibers may undergo acid-catalyzed hydrolysis involving cleavage of amide bonds, leading to deterioration of their mechanical performance [74]. In addition, prolonged chemical exposure may alter dye stability, thereby affecting the appearance of dyed fibers [50]. Strong alkaline conditions may also alter the stability of certain dye systems and contribute to gradual changes in color, depending on the chemical structure of the dye and the duration of exposure [50].

Oxidizing agents, including chlorine-containing and oxygen-based bleaching agents, are among the most significant causes of color degradation in dyed textiles. These compounds primarily attack dye chromophores, leading to fading, discoloration, or complete color loss. Under severe conditions, oxidation may also induce structural changes within the polymer matrix, thereby modifying both the appearance and analytical characteristics of textile fibers [50]. Since many forensic comparisons rely heavily on color evaluation, chemically induced dye degradation represents an important source of potential misinterpretation if the exposure history is unknown [50].

### 4.3. Biological Degradation (Microorganisms, Enzymes)

Compared with natural fibers, synthetic polyamide and acrylic fibers generally exhibit high resistance to biological degradation because their chemical structures are less susceptible to microbial degradation than those of natural polymers [29,34]. Nevertheless, prolonged exposure to elevated humidity, contamination with organic matter, or blending with more biologically susceptible fibers may facilitate microbial colonization and subsequent biological deterioration of textile surfaces [34].

Biological degradation of synthetic textile fibers is primarily associated with the activity of microorganisms capable of secreting extracellular enzymes that initiate hydrolytic cleavage of susceptible polymer structures [29,34]. Owing to the chemical structure and physicochemical properties of polyamide and acrylic fibers, these processes generally proceed much more slowly than those occurring in natural fibers [29]. However, prolonged microbial activity may contribute to gradual surface deterioration and facilitate subsequent environmental or chemical degradation processes [34].

### 4.4. Mechanical and Thermal Stress

Mechanical and thermal stresses constitute another important group of factors influencing the long-term integrity of textile fibers. Repeated bending, stretching, abrasion, laundering, and routine wear gradually modify the physical structure of fibers, producing defects that may facilitate subsequent chemical or environmental degradation [50]. Smith and Thompson noted that different degradation mechanisms rarely occur independently and frequently act synergistically, making the interpretation of damaged textile fibers particularly challenging in forensic investigations [34]. Besides gradual degradation associated with normal use, textile fibers encountered in forensic investigations may also exhibit characteristic damage resulting from specific mechanical or thermal events. Williams categorized forensic textile damage into five principal groups: blunt-force damage, sharp-force damage, projectile damage, thermal damage, and chemical damage. Recognition of these characteristic damage patterns may assist forensic scientists in reconstructing the mechanism of damage and the sequence of events associated with a criminal incident [72].

Mechanical damage may alter fiber morphology through fibrillation, surface cracking, or progressive fragmentation [50], while repeated deformation may reduce tensile performance and increase the accessibility of internal fiber regions to moisture and chemical agents. Similarly, thermal stress associated with ironing, drying, or accidental overheating may induce structural relaxation, molecular reorganization, or thermal degradation of textile fibers [50]. In forensic investigations, additional thermal damage may result from fire exposure, producing characteristic physicochemical changes that may assist in the interpretation of textile evidence [34,72].

In practice, environmental, chemical, biological, and mechanical degradation processes rarely occur independently; instead, they frequently act simultaneously, with one degradation mechanism facilitating or accelerating another [34]. Consequently, forensic textile fibers typically exhibit the cumulative effects of multiple stressors acquired throughout their service life. The resulting physicochemical changes influence not only the polymer structure but also the stability of dye–fiber interactions. Together, these changes constitute the physicochemical condition of the fiber and should therefore be taken into account during the forensic examination of textile evidence [50,70].

The evaluation of degraded fiber evidence should also consider case-specific factors, including the circumstances of transfer, persistence, the time elapsed between the incident and evidence collection, and the consistency of fiber examination results with other forensic findings. Together with the physicochemical condition of the fiber, these contextual factors influence the assessment of the evidential value of textile traces and contribute to reliable forensic conclusions [71,72].

**Figure 7:**
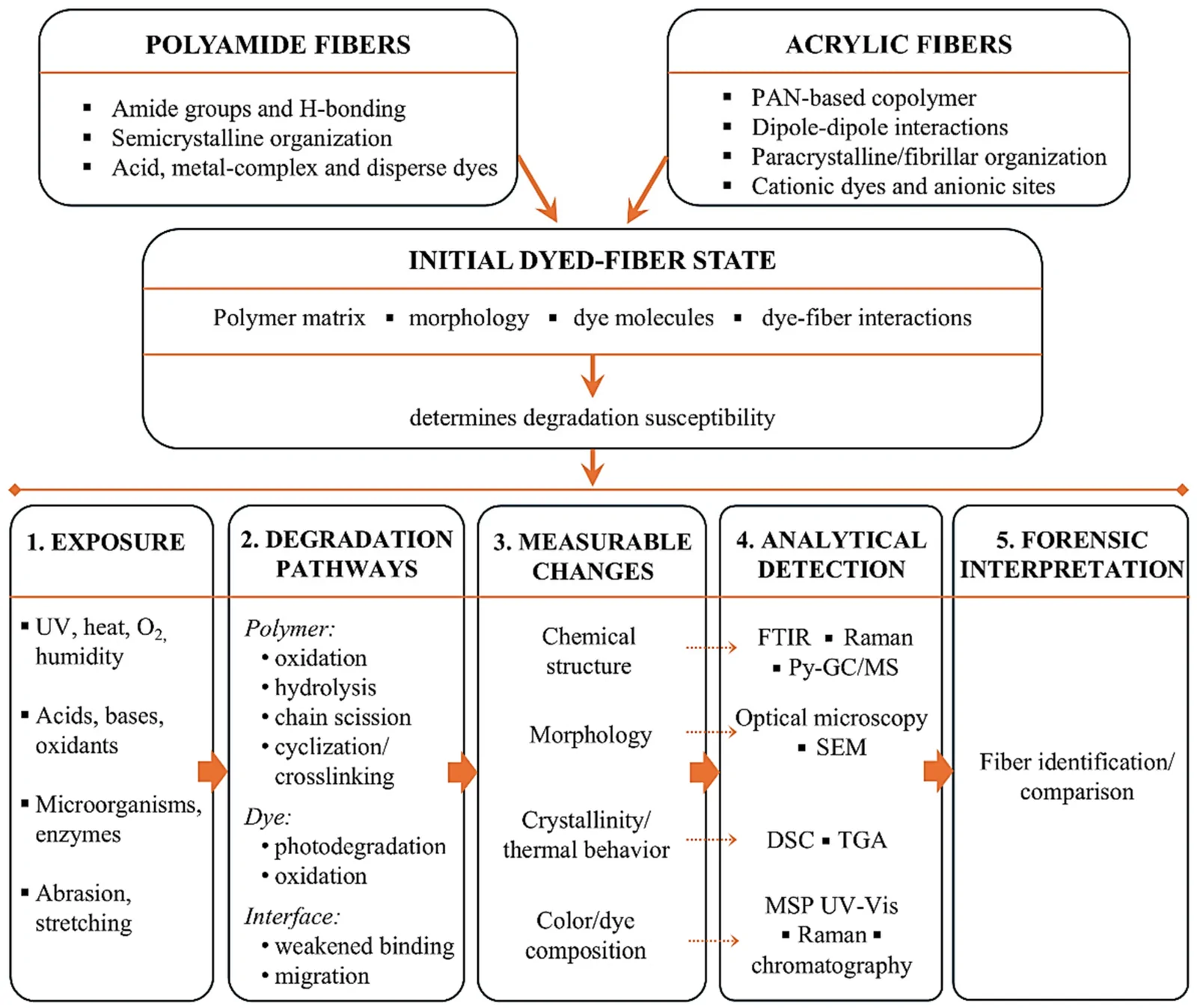
Integrated conceptual framework linking the initial structure of dyed polyamide and acrylic fibers and exposure conditions with degradation pathways, measurable physicochemical changes, analytical detection, and forensic interpretation.

Figure 7 integrates the material-specific characteristics and principal exposure categories discussed above with the degradation pathways, measurable physicochemical changes, analytical approaches, and forensic implications addressed in the following sections.

## 5. Degradation Mechanisms in Polyamide Fibers

### 5.1. Hydrolytic Degradation

**Figure 8:**
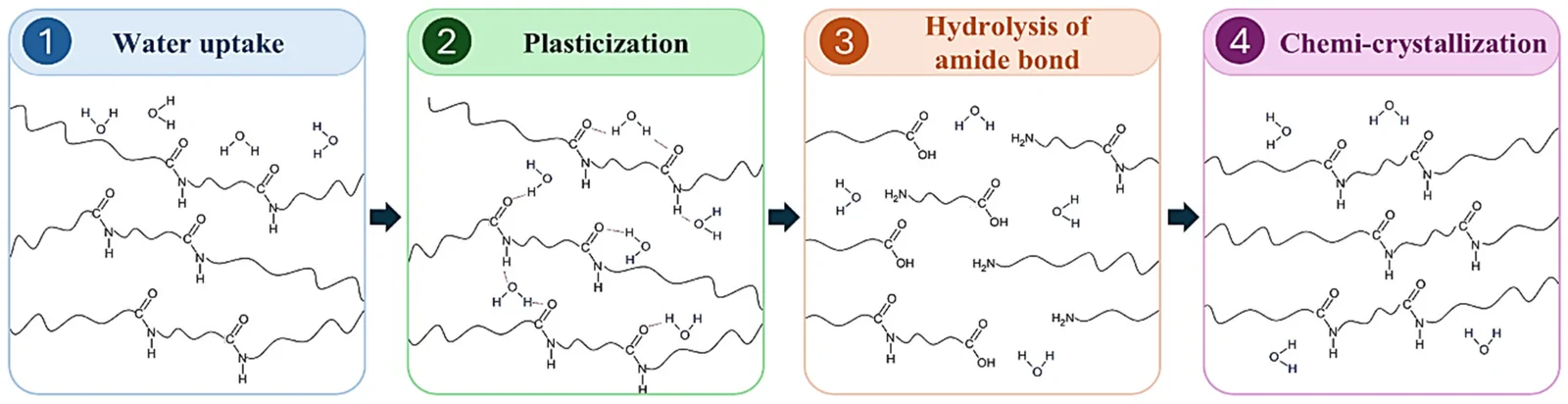
Simplified schematic representation of the main stages of hydrolytic degradation of polyamides, including water uptake, plasticization, amide-bond hydrolysis, and chemicrystallization.

Contact between polyamide fibers and water causes a series of interrelated physical and chemical changes. Their course depends on both environmental conditions and fiber structure. Hydrolytic degradation can be described as a sequence of processes involving water sorption, plasticization, hydrolysis of amide bonds, and reorganization of the polymer structure (Figure 8).

The first stage is the diffusion of water into the fiber. Polar amide groups form hydrogen bonds with water molecules. Water penetrates mainly into the amorphous phase, where the chains are less ordered and more loosely packed than in crystalline regions. The amorphous phase is therefore more accessible to both water sorption and subsequent hydrolysis. After the material becomes saturated, water molecules are incorporated into the hydrogen-bonding network between the polar groups of the polyamide [27]. Polyamides have a relatively high affinity for water. For PA6 and PA66, water contents exceeding 8 wt% have been reported at 100% relative humidity and room temperature. However, values obtained for bulk samples cannot be directly applied to textile fibers. In fibers, sorption also depends on chain orientation and crystallinity [75].

The initial effect of water uptake is plasticization. Water molecules compete with the hydrogen bonds between adjacent polyamide chains and weaken some intermolecular interactions. This increases segmental mobility, causes swelling, and lowers the glass transition temperature. The fiber may therefore become more flexible and easier to deform. These changes are mainly physical and may be partly reversible after drying. Moisture sorption should therefore not be equated with chemical degradation of the polymer chains [27,76,77].

At sufficient water content, hydrolysis of the amide bonds in the polymer backbone may begin. This process is particularly important during prolonged exposure, at elevated temperatures, and under acidic or alkaline conditions. Higher temperature increases the reaction rate, while acids and bases may catalyze the process [27]. A water molecule interacts with the electrophilic carbon atom of the carbonyl group. The formation of an intermediate structure, proton transfer, and cleavage of the C–N bond then lead to chain scission. Two shorter fragments are formed, terminating in an amino group and a carboxyl group, respectively. Brette et al. described this process as water-assisted hydrolysis of amide groups, accompanied by proton transfer and the formation of new end groups [27]. Polyamide hydrolysis is reversible. The newly formed amino and carboxyl groups may react again, restoring an amide bond and releasing water.

At the beginning of exposure, amide-bond cleavage predominates because the material contains many backbone bonds but relatively few end groups. As hydrolysis proceeds, the concentration of amino and carboxyl groups increases, and condensation competes more effectively with chain cleavage. Molecular weight may therefore decrease rapidly at first and then gradually approach an equilibrium value. This behavior has been observed for PA66 aged in water at 60–90 °C [75].

Chain scission usually occurs randomly within the accessible amorphous phase. It is therefore not limited to the gradual removal of units from the ends of macromolecules. Lottier et al. showed that dispersity remained almost constant during hydrolysis of polyamide at 80 °C despite a marked decrease in molecular weight. This was interpreted as evidence of random chain scission. In the same study, water saturation was reached before any substantial decrease in molecular weight occurred. Once the water content had equilibrated, the process rate was therefore no longer controlled solely by diffusion [78].

The small diameter of fibers shortens the water transport path and may promote more uniform hydration across the cross-section than in bulk samples. Studies of PA66 specimens of different thicknesses showed that thinner materials degraded more uniformly throughout their volume. In thicker samples, hydrolysis began near the surface and gradually progressed toward the core. These results cannot be directly equated with the behavior of textile fibers, but they demonstrate the importance of material geometry in the spatial progression of degradation [79]. The hydrolysis rate therefore depends on water availability, exposure time, temperature, and pH, as well as on the amorphous fraction and material geometry.

Shortening of the macromolecules increases the mobility of the resulting segments. Some of them may reorganize, form new ordered regions, or become incorporated into existing crystallites. This increases the crystalline fraction in a process known as chemicrystallization. El-Mazry et al. showed that hydrolytic chain scission disrupts the entanglement network in the amorphous phase and releases shorter segments capable of local ordering [75]. Brette et al. associated chemicrystallization with the greater mobility of shortened chains and the formation of new hydrogen bonds involving end groups and bound water [27].

An increase in crystallinity should not be interpreted as an improvement in material condition. Chemicrystallization occurs alongside chain shortening and a reduction in the number of entanglements and tie molecules connecting crystalline regions. Changes initiated at the molecular level may therefore impair fiber properties [27,75]. Changes in molecular weight, crystallinity, surface morphology, and mechanical properties are discussed in detail in Section 7.

In addition to hydrolysis induced by water and temperature, enzymatic cleavage of amide bonds is also possible. Enzymes capable of hydrolyzing nylon oligomers and polyamide surfaces have been described, along with isolated reports of PA6 fiber degradation by fungi. Examples include a polyamidase from *Beauveria brongniartii* [80] and ligninolytic fungi [81] capable of interacting with PA6 fibers. A polyamide hydrolase from bacteria of the genus *Nocardia* may partially cleave amide bonds and generate amino and carboxyl groups on the material surface [26]. Many of the best-characterized enzymes, including NylA, NylB, and NylC, have mainly been studied for the hydrolysis of 6-aminohexanoate dimers and oligomers. The possibility of effectively degrading commonly used high-molecular-weight polyamides by enzymatic means, particularly in the form of highly ordered fibers, has not yet been confirmed [26].

Typical PA6 and PA66 fibers therefore remain relatively resistant to biodegradation under common environmental conditions [26,34]. Results obtained for low-molecular-weight oligomers may not directly reflect how readily highly oriented textile fibers undergo degradation [26,34]. Synthetic nylon fibers are also generally resistant to mold growth, although organic soiling, blends with more susceptible fibers, and certain finishing treatments may promote microbial colonization [34].

### 5.2. Photo-Oxidation Pathways

Polyamide fibers are often exposed during use to sunlight, atmospheric oxygen, and elevated temperatures. These factors may act simultaneously and cause gradual material aging. When light exposure occurs in the presence of oxygen, photo-oxidation takes place and constitutes an important degradation pathway in aliphatic polyamides [82,83]. Aliphatic polyamides do not contain typical chromophoric groups and therefore absorb solar radiation only weakly. The initiation of photo-oxidation is usually associated with impurities, structural defects, or other components in the material that are capable of absorbing radiation. Once excited, these species may initiate radical reactions in the polyamide chain [84].

**Figure 9:**
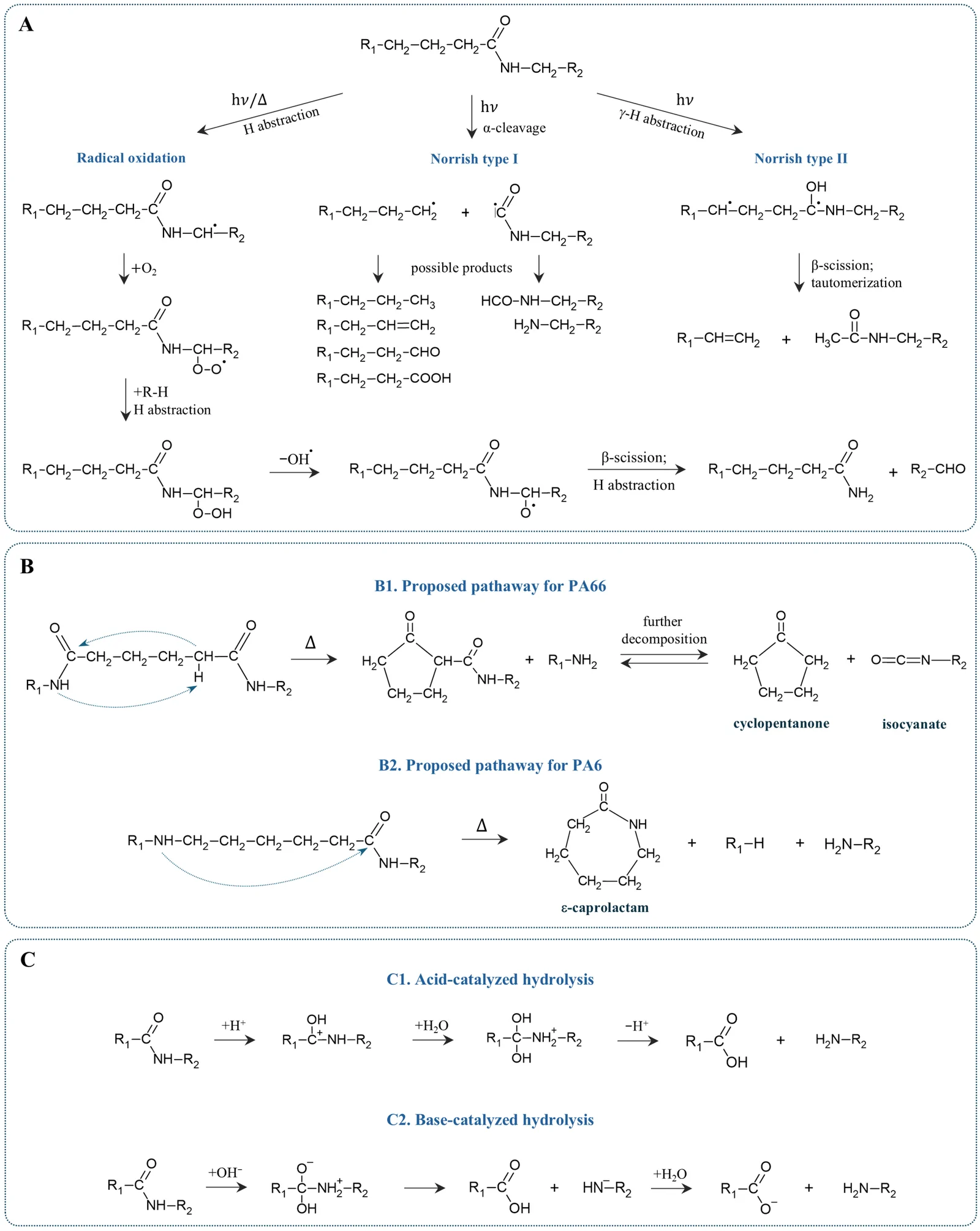
Simplified schemes of the main degradation pathways of polyamides: (**A**) photo-oxidative degradation, including radical oxidation and Norrish type I and II reactions; (**B**) thermal degradation pathways proposed for PA66 (**B1**) and PA6 (**B2**); and (**C**) acid-catalyzed (**C1**) and base-catalyzed (**C2**) hydrolysis of amide bonds. R_1_ and R_2_ denote the continuation of the polymer chain beyond the fragment shown.

The general course of polyamide photo-oxidation involves radical initiation, oxygen addition, hydroperoxide formation, and subsequent hydroperoxide decomposition. Reactions leading directly to chain scission may occur in parallel, including Norrish type I and type II reactions [82,84]. The main pathways involved in photo-oxidation are shown schematically in Figure 9A.

The first stage involves radical formation in the polyamide chain. Hydrogen atoms attached to methylene groups directly adjacent to the amide nitrogen are particularly susceptible to abstraction. Alkyl radicals therefore form preferentially at these positions and initiate further oxidation reactions [84,85]. The polymer radical then reacts with molecular oxygen to form a peroxy radical. This radical may abstract a hydrogen atom from another chain segment, producing a hydroperoxide and a new polymer radical. In this way, the chain reaction propagates [84].

Hydroperoxides subsequently decompose under irradiation or elevated temperatures. This produces more reactive species, particularly alkoxy radicals. They may participate in further hydrogen abstraction, rearrangement, and β-scission reactions, leading to polymer chain cleavage [82,85].

In addition to hydroperoxide formation and decomposition, polyamides may undergo Norrish type I and type II reactions. These photochemical reactions involve excitation of carbonyl groups and lead to polymer chain scission. In a Norrish type I reaction, the bond adjacent to the carbonyl group undergoes homolytic cleavage, producing two radicals. A Norrish type II reaction involves transfer of a hydrogen atom from the γ-position to the oxygen atom of the excited carbonyl group, followed by rearrangement and cleavage of the resulting structure. Both pathways generate different end groups and unsaturated products [82].

Studies of PA6 photo-oxidation products showed that hydroperoxide decomposition and Norrish type I and II reactions may occur simultaneously. Polyamide photo-oxidation therefore does not follow a single pathway but involves several competing reactions that generate radicals and cause chain scission [82].

The resulting radicals may continue to participate in propagation reactions or undergo termination. Termination reactions remove active radical centers, while oxidation may also be accompanied by local cross-linking of the material [27,85]. At the same time, β-scission, hydroperoxide decomposition, and Norrish reactions lead to chain cleavage and a decrease in the molar mass of the polyamide [82,85].

The rate of photo-oxidation and the relative contribution of individual pathways depend on radiation wavelength and exposure time, oxygen availability, temperature, and material properties. Shorter wavelengths may cause direct bond cleavage, whereas at wavelengths above approximately 290 nm, initiation by photosensitizers present in the polymer becomes more important [84]. Elevated temperatures accelerate hydroperoxide decomposition and subsequent radical reactions [85,86]. Oxygen availability also affects the rate and spatial distribution of oxidation, as limited diffusion may lead to non-uniform degradation [78]. Prior oxidation products and additives are also important. Some may initiate photo-oxidation or act as photosensitizers, whereas radical scavengers and hydroperoxide decomposers may inhibit the process [84].

The effects of these transformations on molecular weight, crystallinity, surface morphology, and mechanical properties are discussed in detail in Section 7.

### 5.3. Thermal Degradation Behavior

Polyamide fibers may be exposed to elevated temperatures during both use and processing. Depending on temperature and oxygen availability, this may lead either to slow thermo-oxidative aging or to extensive polymer chain degradation. Under service conditions, when the material is heated in air, thermo-oxidation usually predominates. In an inert atmosphere or under vacuum, purely thermal degradation occurs and proceeds much more slowly at moderate temperatures. Only at higher temperatures does extensive macromolecular breakdown occur, accompanied by the formation of volatile products. This process is referred to as pyrolysis. In studies of PA66 conducted in the absence of oxygen, no significant mass loss was observed up to 240 °C, although limited backbone scission and radical formation were detected [87].

At the initial stage of thermal degradation, a hydrogen atom is abstracted from the methylene group adjacent to the amide nitrogen. The C–H bond at this position has a lower dissociation energy than those in other methylene groups and therefore undergoes homolytic cleavage more readily. This produces a carbon-centered radical at the α-position relative to nitrogen. In the presence of oxygen, it rapidly forms a peroxy radical, which participates in subsequent reactions leading to hydroperoxide formation. Hydroperoxide decomposition generates further radicals and may cause chain scission, carbonyl formation, and the release of low-molecular-weight products [85,87]. The general mechanism of thermo-oxidation is therefore similar to that of photo-oxidation, but the mode of initiation differs. Here, radical formation is induced by heat rather than radiation. The process involves radical initiation, oxygen addition, hydroperoxide formation and decomposition, propagation, and termination reactions (Figure 9A) [84].

When oxygen availability is limited, carbon-centered radicals do not enter the peroxide cycle and may instead undergo direct β-scission. This causes homolytic cleavage of the polymer chain and produces new radicals at the ends of the resulting fragments. Pathways leading to alkyl and acyl radicals have been described for PA66 [87]. In the presence of oxygen, chain scission may also result from hydroperoxide decomposition and subsequent reactions of alkoxy radicals. These pathways may produce aldehydes and secondary amines, while further oxidation leads to more highly oxidized products and CO_2_ release [87,88].

At substantially higher temperatures, both the rate and the nature of degradation change. For PA6, one of the main pathways is depolymerization to caprolactam. Cyclic and linear oligomers, nitrile- or vinyl-terminated compounds, and smaller amounts of CO_2_, CO, H_2_O, NH_3_, and HCN may also form [89]. PA66 cannot regenerate a single cyclic monomer in the same way. Its degradation produces a more complex mixture, including cyclopentanone and its derivatives, ammonia, and condensation products [90]. Simplified schemes of the main high-temperature degradation pathways of PA6 and PA66 are shown in Figure 9B.

Elevated temperature may induce chemical changes while also allowing structural reorganization of the polymer. The observed increase in crystallinity may result both from post-crystallization of chains initially present in the amorphous phase and from chemicrystallization associated with chain shortening and the release of previously entangled segments capable of local ordering. An increase in crystallinity should not, however, be interpreted as an improvement in material condition, because it is accompanied by the loss of entanglements and a decrease in molecular weight. The possibility of further crystallization during differential scanning calorimetry should also be considered. Studies of PA6 showed that a substantial part of the apparent increase in crystallinity may occur during heating in differential scanning calorimetry (DSC), rather than during prior material aging [84].

The rate and spatial distribution of degradation depend on temperature, exposure time, oxygen availability, polyamide structure, and material geometry. In thicker samples, oxygen consumption may exceed its diffusion into the interior, causing oxidation to concentrate near the surface [78]. Because fibers have small diameters, oxygen diffusion limitations may be less important than in bulk specimens. Additives also influence the process. Radical scavengers and hydroperoxide decomposers may inhibit oxidation, although some antioxidants are less effective in polyamides than in polyolefins [84]. Thermal degradation of polyamides therefore includes both slow thermo-oxidative aging under service conditions and extensive breakdown at high temperatures. Both processes alter the chemical structure and gradually impair material properties [84,89].

### 5.4. Chemical Attack and Chain Scission Mechanisms

Polyamide fibers may be exposed to acids, bases, and oxidizing agents during use, washing, cleaning, or industrial processing. Depending on the reagent, chemical attack primarily leads either to hydrolysis of amide bonds or to radical reactions resulting in polymer chain scission.

In acidic or alkaline media, amide-bond hydrolysis proceeds faster than in water at neutral pH. Under acidic conditions, protonation of the carbonyl oxygen increases the electrophilicity of the carbon atom and facilitates attack by water. Under alkaline conditions, the hydroxide ion acts as the nucleophile. Simplified acid- and base-catalyzed hydrolysis pathways are shown in Figure 9C.

In both cases, the formation of an intermediate and cleavage of the C–N bond result in chain scission and the formation of fragments terminated with amino and carboxyl or carboxylate groups. The process occurs mainly in the more accessible amorphous phase and causes a gradual decrease in molecular weight [27]. Polyamides nevertheless show relatively good resistance to short-term exposure to many reagents. The extent of damage therefore depends on concentration, temperature, and exposure time. In studies of PA66 fibers, KOH caused much smaller changes than in PET, and some of the observed effects may also have resulted from ion sorption in the amorphous phase [91].

Oxidizing agents such as hydrogen peroxide, hypochlorites, and ozone may directly or indirectly initiate radical reactions in the polyamide chain. Hydrogen abstraction occurs preferentially at the methylene group adjacent to the nitrogen atom. The resulting radical then undergoes reactions involving oxygen addition, hydroperoxide formation and decomposition, and β-scission. These transformations are shown schematically in Figure 9A [85,88]. In the presence of hydrogen peroxide and transition metal ions, highly reactive radicals, including hydroxyl radicals, may be generated, as in the Fenton reaction [91]. Transition metal ions may also catalyze the decomposition of hydroperoxides into reactive radicals, promoting further oxidation and chain scission, although in studies of PA66, the presence of Fe(III) ions in water did not produce a measurable increase in the degradation rate [92]. Ozone also promotes radical initiation and the formation of oxygen-containing groups in the polyamide structure. PA66 is less resistant to ozone than polyamides containing aromatic rings [93]. Hypochlorites and other bleaching agents may oxidize amide and amino groups and initiate secondary radical reactions. The course of these processes depends on pH, concentration, and temperature [34].

Regardless of the reaction pathway, chemical attack ultimately shortens the macromolecules and increases the number of end groups. These changes may occur without obvious surface damage. The absence of visible dissolution or morphological alteration therefore does not indicate that the chemical integrity of the fiber has been preserved. In practice, polyamide resistance depends not only on the reagent but also on exposure conditions, fiber chemistry, and the accessibility of the amorphous phase.

## 6. Degradation Mechanisms in Acrylic Fibers

Commercial acrylic fibers are generally based on PAN copolymers rather than PAN homopolymer. In the following sections, the term PAN is used when referring specifically to the polymer or to mechanistic studies conducted on PAN model systems, whereas acrylic fibers refer to commercial PAN-based textile fibers.

### 6.1. Thermo-Oxidative Degradation Pathways

Under moderate conditions, structural changes in acrylic fibers usually proceed slowly and do not necessarily cause rapid loss of integrity [94]. More pronounced transformations of PAN occur when additional energy is supplied, for example, through heating or irradiation [95,96].

**Figure 10:**
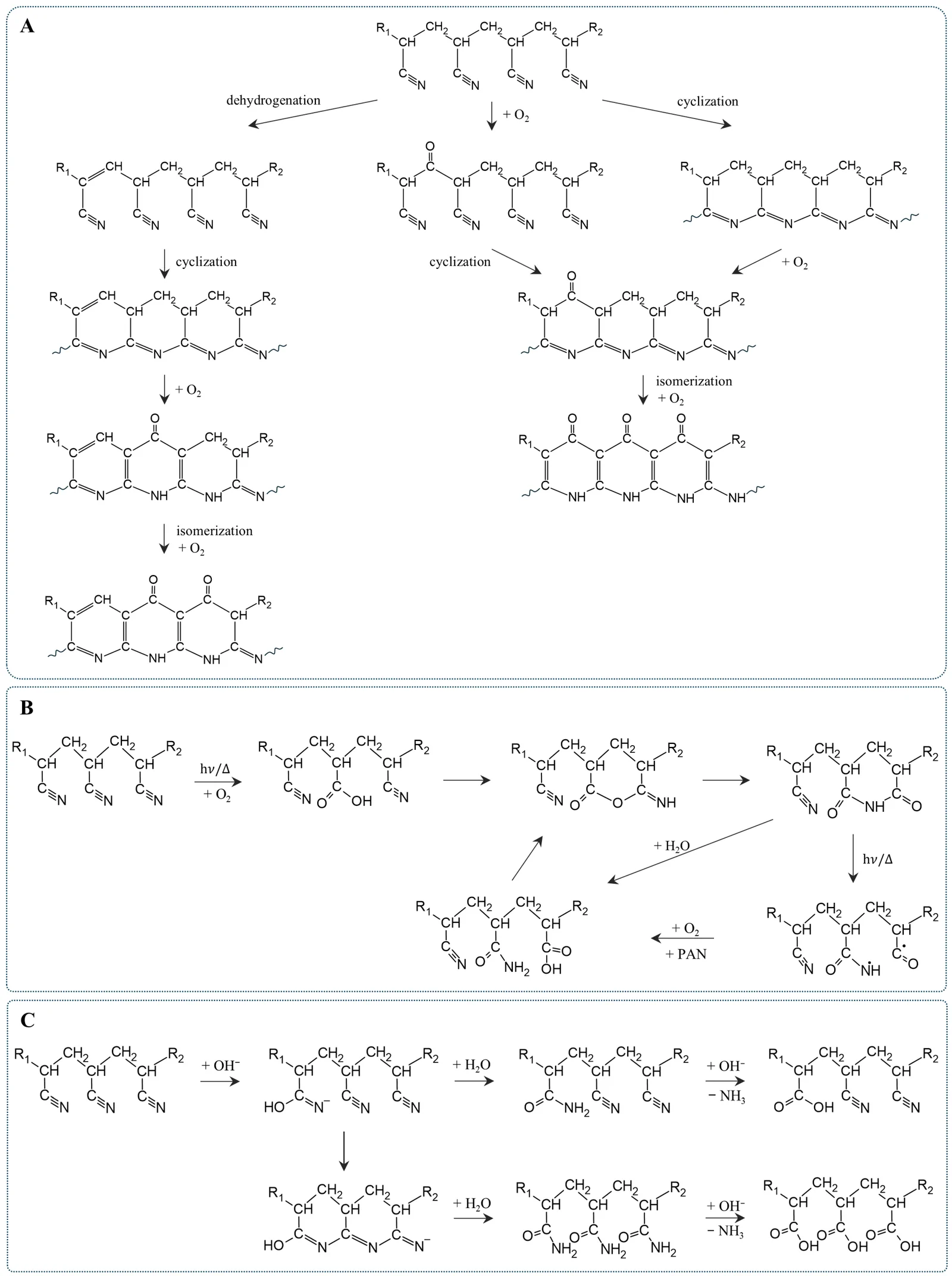
Simplified schemes of the main degradation pathways of polyacrylonitrile (PAN): (**A**) thermo-oxidative degradation, including cyclization, dehydrogenation, oxygen uptake, and isomerization; (**B**) photo-oxidative transformations; and (**C**) alkaline hydrolysis of nitrile groups. R_1_ and R_2_ denote the continuation of the polymer chain beyond the fragment shown, while wavy bonds indicate the extension of cyclic structures beyond the displayed fragment.

Thermo-oxidative degradation of acrylic fibers involves several interconnected reactions, including cyclization of nitrile groups, dehydrogenation, oxygen uptake, and crosslinking. More intense heating also promotes structural fragmentation and the release of low-molecular-weight products. These processes are usually discussed in the context of thermal stabilization of precursors used in carbon fiber production. From a forensic perspective, however, they represent degradation because they permanently alter the chemical structure of the fiber. Their course depends primarily on temperature, heating time, oxygen availability, and polymer composition [97,98,99]. The main pathways of PAN thermo-oxidative degradation, including cyclization, dehydrogenation, oxygen uptake, and their interrelationships, are shown in Figure 10A.

One of the main reactions occurring during PAN heating is cyclization of adjacent nitrile groups. This converts linear chains into conjugated structures containing six-membered nitrogen heterocycles. In the PAN homopolymer, cyclization is initiated mainly through a free-radical mechanism involving initiation, propagation, and termination steps. At 200–230 °C, chain ends, particularly those containing conjugated nitrile groups, play an important role in initiating the reaction. As the temperature increases, cyclization becomes faster, but termination reactions also become more frequent and may produce β-aminonitrile groups [96,97].

In textile acrylic fibers, PAN is usually present as a copolymer. Acidic comonomers such as itaconic acid may lower the onset temperature of cyclization and initiate the process through an ionic pathway. During heating, itaconic acid is partly converted into an anhydride, which facilitates the initiation of nitrile-group reactions [96].

In the presence of oxygen, cyclization occurs alongside dehydrogenation and oxidation. Dehydrogenation removes hydrogen atoms through the elimination of water and forms C=C bonds in the polymer backbone, increasing conjugation within the developing structure. Oxygen may be incorporated into both linear PAN segments and previously formed rings. These reactions produce carbonyl, hydroxyl, and ether groups, as well as oxidized heterocyclic structures [97,99]. Cyclization, dehydrogenation, and oxygen uptake initially proceed in parallel. As the degree of cyclization increases, dehydrogenation and oxidation gradually become more prominent [97].

Cyclization, dehydrogenation, and oxidation produce a crosslinked ladder structure containing C=C and C=N units. The development of extended conjugated systems also accounts for the progressive discoloration of PAN during heating [95,99]. Under thermolytic conditions, polyimine structures and their enamine forms have been observed. In the presence of oxygen, these structures undergo further oxidation, probably forming nitrone- and pyridone-like structures [95].

Oxygen incorporation may be non-uniform across the fiber cross-section. Reactions begin at the surface, while their progression in the interior depends on oxygen diffusion. When surface reactions proceed faster than oxygen transport into the fiber, a skin–core structure may develop. The outer layer and the core then differ in their degree of cyclization and oxidation. High temperature and short exposure favor this heterogeneity, whereas longer heating may partly reduce differences across the fiber cross-section [98].

At higher temperatures, fragmentation and the release of volatile products become increasingly important. Cyclization and nitrile-group transformations may be accompanied by the release of HCN and NH_3_, while decomposition of oxidized fragments produces H_2_O, CO, and CO_2_. Volatile organic products and tar-like fractions have also been reported. Their type and amount depend on the prior degree of cyclization and oxidation. Pre-oxidized fibers release less HCN and NH_3_ because they contain fewer unreacted nitrile groups. At the same time, they may release more CO_2_ because of the decomposition of oxygen-containing groups already present in the structure [100].

Under moderate heating, cyclization, dehydrogenation, oxidation, and crosslinking therefore predominate. At higher temperatures, fragmentation and the release of low-molecular-weight products become increasingly significant.

### 6.2. UV-Induced Changes

Polyacrylonitrile is relatively resistant to sunlight. UV-induced changes may therefore proceed slowly and depend strongly on exposure conditions. Photolysis of PAN in the absence of oxygen produced no pronounced changes, whereas irradiation in the presence of oxygen led to gradual polymer oxidation [95]. Under environmental conditions, the dominant process is therefore not direct photolytic breakdown, but photo-oxidation. Radiation initiates the formation of reactive sites, while oxygen participates in subsequent reaction steps. The rate of the process depends on wavelength, temperature, exposure time, and fiber composition, including the presence of comonomers, dyes, and UV stabilizers [94,95].

UV radiation may generate radicals in PAN chains, promoting cyclization of adjacent nitrile groups, dehydrogenation, and formation of conjugated polyene structures. These reactions may also involve conversion of C≡N groups into C=N groups and formation of amino groups. As degradation proceeds, intermolecular crosslinking may also occur, resulting in the formation of a crosslinked surface layer [101,102]. UV-induced transformations of PAN reported in the literature include both cyclization and crosslinking, as well as reactions leading to polymer chain scission [95,101,102,103].

In the presence of oxygen, radiation-generated radicals may initiate oxidation reactions. Hydroperoxides have been proposed as the first products, although their concentration remains low because they rapidly undergo further transformation. The resulting carboxyl groups may react with adjacent nitrile groups, forming cyclic structures and subsequently imide groups. Imides are photochemically unstable and may decompose into radicals. Further oxidation of these radicals produces amide and carboxyl groups. The newly formed acid groups may then react with additional nitrile groups, leading to further cyclic transformations. Photo-oxidation therefore produces mainly amide, imide, and carboxyl groups in the PAN structure [95]. A proposed scheme for these transformations is shown in Figure 10B. Unlike thermo-oxidation, photo-oxidation did not produce substantial amounts of polyimine structures or NH_3_ [95].

At shorter wavelengths, direct cleavage of bonds in the polymer chain may also occur. Irradiation of PAN at 254 nm produced hydrogen, hydrocarbon fragments, acrylonitrile, and trace amounts of HCN [95]. Under conditions closer to environmental exposure, however, the changes may be much less pronounced [94]. Differences between studies may result from variations in radiation intensity and spectrum, exposure time, fiber composition, and the presence of water and additives [94,95,103]. PAN may therefore remain relatively resistant to sunlight under moderate conditions, while more intense or prolonged exposure may promote cyclization, crosslinking, oxidation, and chain scission [95,101,102,103].

### 6.3. Chemical Degradation Pathways

Polyacrylonitrile is relatively resistant to hydrolysis, which therefore proceeds slowly under mild conditions. Nitrile groups may nevertheless undergo hydrolysis under more severe conditions, including exposure to strong acids or bases, elevated temperatures, and prolonged treatment [104]. Alkaline hydrolysis is the best-characterized pathway. During this process, nitrile groups are gradually converted into amide and subsequently into carboxyl or carboxylate groups [104,105,106].

Alkaline hydrolysis begins with the nucleophilic addition of a hydroxide ion to the carbon atom of the nitrile group. This produces an intermediate containing a C=N bond, which is then converted into an amide group. Further hydroxide attack hydrolyzes the amide and forms a carboxyl group, present mainly as a carboxylate under alkaline conditions [105]. As the reaction proceeds, the proportion of nitrile groups decreases, while the content of amide and carboxylate groups increases. The process does not necessarily follow a single linear pathway through one intermediate. Six-membered cyclic structures may also form during alkaline hydrolysis of PAN through reactions between adjacent functional groups [104]. A simplified scheme of alkaline hydrolysis of PAN nitrile groups is shown in Figure 10C.

PAN may also undergo hydrolysis under acidic conditions, although this process differs from alkaline hydrolysis in both mechanism and product structure. The first step is protonation of the nitrile nitrogen, which makes the –C≡N group more susceptible to subsequent reactions leading to amide formation. In concentrated acids at moderate temperatures, the main products are copolymers containing acrylonitrile and acrylamide units, while the proportion of acrylic acid units usually remains low. The reaction does not proceed randomly along the chain. Neighboring amide groups strongly accelerate hydrolysis of adjacent nitrile groups, leading to multiblock structures. At elevated temperatures, amide groups may also cyclize to form glutarimide structures [107].

In acrylic fibers, hydrolysis depends not only on reagent concentration and temperature, but also on the accessibility of nitrile groups and copolymer composition. Ester-containing comonomers may also undergo hydrolysis and generate additional carboxyl groups. The reaction initially occurs mainly in the more accessible surface regions, whereas increasing the duration or severity of the treatment leads to progressively more extensive changes in the material [104,108].

Prior oxidation, cyclization, and crosslinking of PAN reduce the proportion of unreacted nitrile groups and increase the material’s resistance to strong bases. Unreacted nitrile groups may nevertheless continue to hydrolyze into amide and carboxyl groups [108]. The prior history of the fiber, including exposure to heat or oxygen, may therefore affect its susceptibility to subsequent chemical degradation.

Under environmental conditions, water or mildly acidic or alkaline media alone are unlikely to cause rapid hydrolysis of acrylic fibers. Substantial transformations usually require a suitable pH combined with elevated temperature, prolonged exposure, or a high reagent concentration [104,105,107]. Chemical degradation of PAN-based fibers is therefore more likely during contact with strong cleaning agents, laboratory reagents, or industrial chemicals than during ordinary environmental aging.

### 6.4. Stability Comparison with Polyamides

Sait et al. directly compared the fibers under conditions intended to simulate environmental exposure. After five and ten months of simulated UV exposure in seawater, the mean length of the polyamide fibers decreased by 62.2% and 74.3%, respectively. A similar pattern was observed in freshwater, and the differences between the two media were not statistically significant. In contrast, PAN-based fibers showed no significant change in length or any evidence of fragmentation. The authors therefore concluded that PAN-based fibers may be more environmentally persistent than PA fibers under the same conditions [94].

Similar differences have been observed during exposure to selected chemical reagents. In studies involving several procedures for digesting organic matter, acrylic fibers showed the greatest overall resistance among the materials examined. Their tensile strength and elongation at break remained largely stable, with only minor changes after treatment with KOH and 30% H_2_O_2_. Under the same conditions, hydrogen peroxide caused marked mechanical degradation of PA66 and a loss of fiber integrity [91].

**Figure 11:**
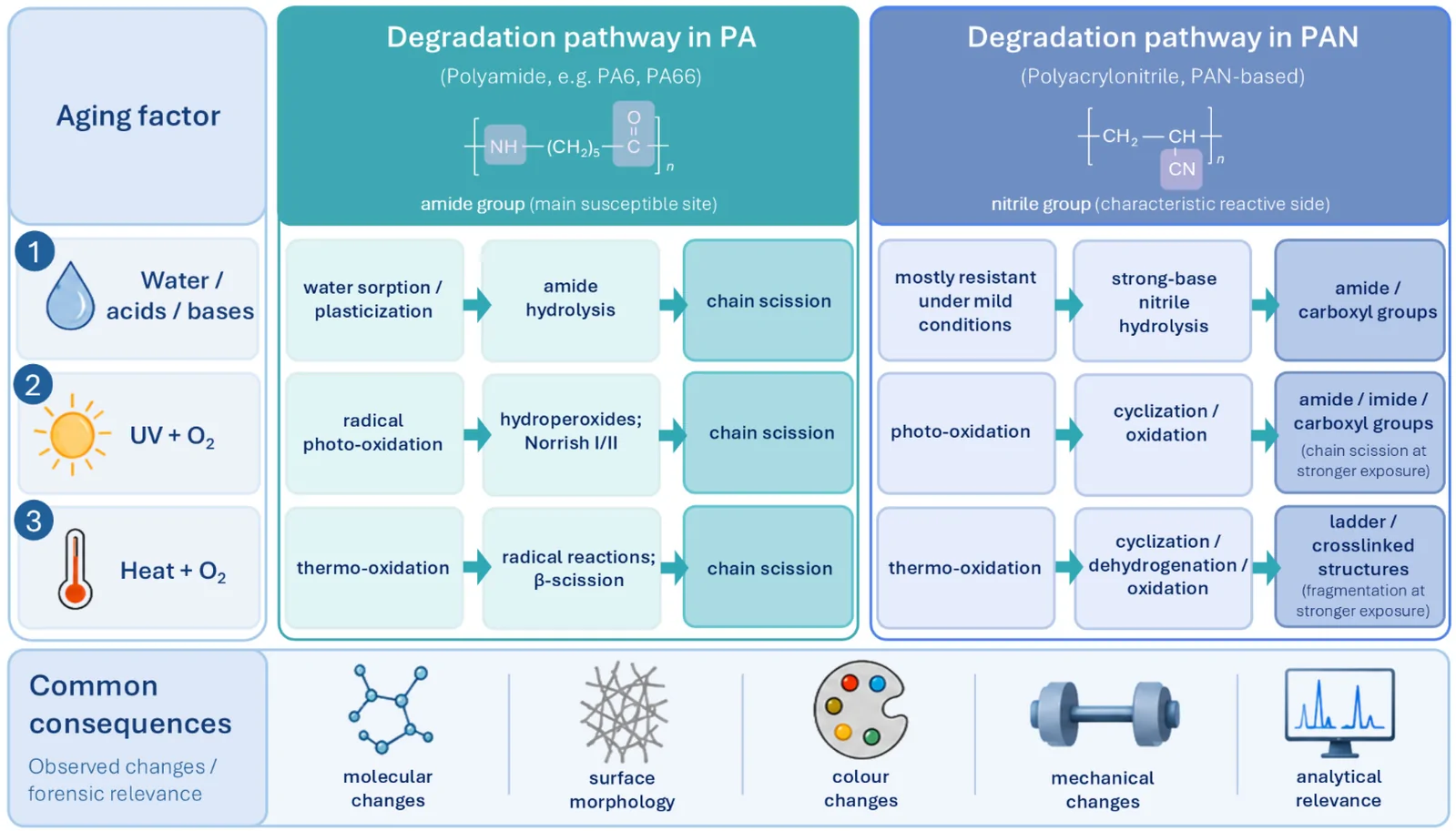
Horizontal comparison of the main degradation pathways of polyamide (PA) and polyacrylonitrile (PAN) fibers under hydrolytic/chemical, photo-oxidative, and thermo-oxidative conditions. The scheme highlights the principal reactive sites, dominant degradation routes, and their common physicochemical and forensic consequences.

The contrasting behavior of PA and PAN reflects fundamental differences in their chemical structure and dominant degradation pathways. A simplified horizontal comparison of the main reactions occurring under hydrolytic/chemical, photo-oxidative, and thermo-oxidative conditions is presented in Figure 11.

The apparent greater resistance of PAN-based fibers under moderate exposure conditions is not universal. Under intense UV irradiation, both nylon and acrylic fibers underwent degradation, and in one experiment, the greatest extent of degradation was observed for acrylic fibers. The formation rate of chromophoric dissolved organic matter was higher for PAN-based fibers than for nylon, although the final amount of total dissolved organic carbon was similar for both materials [103]. These differences show that the relative stability of the two fiber types depends on radiation intensity and spectrum, exposure time, the presence of water, copolymer composition, and the dyes and additives used.

## 7. Physicochemical Changes During Degradation

Degradation processes cause changes at different levels of fiber organization. Polymer chain scission or crosslinking affects molecular weight and structural order, which in turn alters surface morphology, color, and mechanical properties. These features do not change independently. A decrease in molecular weight may increase the mobility of shorter chain fragments and promote crystallization, whereas crosslinking may increase material stiffness despite ongoing chemical degradation. The degree of fiber degradation should therefore be assessed by combining molecular, structural, optical, and mechanical evidence. This section examines changes in molecular weight and crystallinity, surface morphology, color, and mechanical properties during fiber degradation.

### 7.1. Changes in Molecular Weight and Crystallinity

In polyamides, hydrolytic and oxidative degradation primarily cause polymer chain scission and a decrease in average molecular weight. During hydrolysis, amide bonds are cleaved, forming new amino and carboxyl end groups. The process initially occurs mainly in the amorphous phase, which is more accessible to water and has greater segmental mobility. During oxidation, chain scission results from radical reactions and hydroperoxide decomposition, although crosslinking may occur in parallel under certain conditions [27,75,86,92].

A decrease in polyamide molecular weight is often accompanied by an increase in the crystalline fraction. Shorter chain fragments formed by hydrolysis or oxidation are more mobile and can reorganize more readily into ordered structures. This process is known as chemicrystallization. It does not restore the original material structure but represents secondary ordering of partially degraded chains. Crystallinity therefore increases alongside molecular weight loss and a reduction in the number of entanglements and tie molecules connecting adjacent crystalline regions [27,75,78,86]. During hydrothermal aging of PA66, molecular weight loss and increased crystallinity were observed from the early stages of exposure [27,75]. Their subsequent development depended on temperature, exposure time, polyamide type, and the presence of additives [27].

Increased crystallinity may initially raise hardness or local stiffness, but it does not indicate improved durability. The newly formed crystallites consist of shorter chains, while the amorphous phase gradually loses its ability to transfer stress. During thermo-oxidative aging of PA66, molecular weight loss, increased crystallinity, and higher microhardness were directly correlated. Chain scission nevertheless remained the dominant molecular process and ultimately led to embrittlement [86].

For PAN, changes in molecular weight and structural order are more difficult to interpret. Chain scission during photodegradation or severe chemical degradation is expected to reduce molecular weight. At the same time, cyclization and crosslinking may generate an insoluble fraction, making conventional molecular weight determination difficult. During thermo-oxidation, linear PAN chains are converted into cyclic and crosslinked ladder structures, while the original chain organization gradually changes [96,97,99].

Interpretation of structural changes in PAN is further complicated by the possible presence of skin–core heterogeneity, discussed earlier. This means that the degree of ordering, cyclization, and oxidation may vary depending on the position analyzed across the fiber cross-section [98]. Changes in PAN crystallinity therefore cannot always be described as a simple, unidirectional increase or decrease.

### 7.2. Surface Morphology Alterations

Changes in surface morphology are among the most direct effects of fiber aging, but they do not always occur simultaneously with transformations at the molecular level. During the early stages of degradation, the surface may still appear unchanged even though chemical or mechanical changes have already begun. As the process progresses, surface irregularities, depressions, pores, scratches, and longitudinal or transverse cracks may develop. Further weakening of the material may lead to the detachment of fragments and shortening of the fibers.

In polyamides, exposure to UV radiation and oxygen may increase surface roughness and lead to the formation of microcracks, fissures, and irregular material loss. Chemical degradation usually begins in the most accessible surface regions. The resulting defects increase the surface area available to environmental agents and promote crumbling of the material. Surface cracks may also act as stress concentration sites, so even minor morphological changes may increase the susceptibility of the fiber to subsequent mechanical damage [94,103,109].

**Figure 12:**
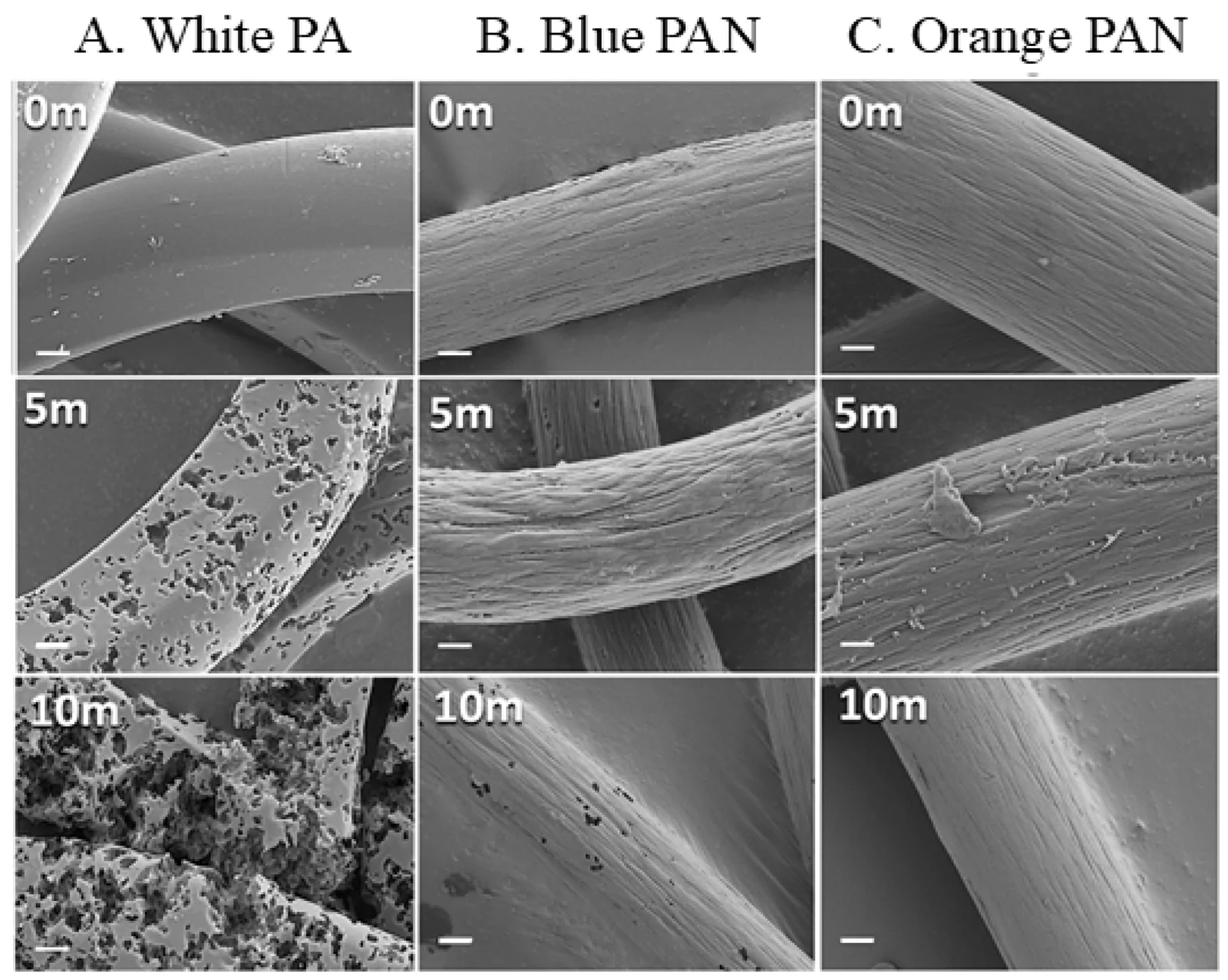
SEM micrographs showing changes in the surface morphology of white polyamide (PA), blue polyacrylonitrile (PAN), and orange PAN fibers before exposure (0 m) and after 5 months (5 m) and 10 months (10 m) of UV irradiation in seawater. Adapted from Sait, S.T.L.; Sørensen, L.; Kubowicz, S.; Vike-Jonas, K.; Gonzalez, S.V.; Asimakopoulos, A.G.; Booth, A.M. Microplastic fibres from synthetic textiles: Environmental degradation and additive chemical content. *Environ. Pollut.* 2021, 268, 115745. [94], published under a Creative Commons Attribution (CC BY) license. The original figure was cropped and the PET panels were removed. Scale bars represents 5 μm.

During long-term exposure in freshwater and seawater, polyamide fibers showed substantial surface damage and fragmentation, whereas changes in PAN were much less pronounced and depended on fiber color [94]. Representative SEM images illustrating these differences are shown in Figure 12. Under more intense UV irradiation, however, both nylon and acrylic fibers underwent clear degradation. In PAN, longitudinal cracks, surface roughening, brittle ends, and detachment of small fragments were observed [103]. This discrepancy indicates that the absence of visible damage under moderate conditions does not imply complete resistance, but may result from insufficient radiation intensity or dose, as well as differences in the composition and properties of the examined material.

Thermo-oxidation of PAN may also produce a non-uniform cross-sectional structure. Differences between the outer layer and the core are associated with oxygen diffusion, the degree of cyclization and crosslinking, and stresses generated during heating. Under more severe conditions, pores, cracks, and other internal defects may develop. These defects reduce fiber strength despite increased stiffness of the surface layer [98].

Morphological changes such as cracks, material loss, and increased roughness are not specific to a single degradation mechanism. They may result from UV exposure, oxidation, hydrolysis, or mechanical stress. Surface morphology should therefore not be interpreted independently of chemical, optical, and mechanical changes in the fiber.

### 7.3. Color Changes and Dye–Fiber Interactions

Changes in fiber color may result from transformations in the polymer itself or from dye degradation. These processes may occur simultaneously and cannot always be distinguished by visual observation alone. Polymer oxidation produces new chromophoric groups, whereas dye degradation may cause fading, a shift in hue, or loss of characteristic spectral features.

Thermal and oxidative degradation of polyamides is often accompanied by yellowing. This is associated with the formation of oxidized and conjugated structures that absorb visible light. Color change may appear before other material properties deteriorate markedly and may therefore serve as an indicator of early aging. Its intensity does not necessarily correlate directly with molecular weight loss or strength loss, because individual degradation products differ in stability and optical properties [27,85,86]. During thermo-oxidation of PA66, yellowing was associated with chemical changes, increased crystallinity, and higher microhardness [86].

During heating, PAN may change from white to yellow, brown, dark brown, or black. This discoloration is mainly associated with the development of conjugated cyclic and ladder structures formed during cyclization and dehydrogenation. The degree of darkening therefore reflects the progress of structural transformation, although it is not an unambiguous measure of degradation. It also depends on copolymer composition, temperature, heating time, and oxygen availability [97,98,99].

Dyes may either accelerate or inhibit fiber photodegradation. A dye molecule that absorbs radiation may enter an excited state and initiate the formation of radicals or reactive oxygen species. This leads to photosensitization of the polymer, commonly referred to as phototendering. Alternatively, the dye may absorb and dissipate radiation energy without initiating chemical reactions, acting as a filter that limits UV exposure of the polymer. The final effect depends on dye structure, concentration, fiber binding, and absorption range [76,110].

The importance of these interactions was demonstrated in studies of dyed nylon 6 yarns. Dye type was the main factor affecting the degradation rate during artificial aging, and the difference in strength loss between the most and least resistant samples was approximately threefold. This effect was attributed mainly to differences in the ability of the dyes to screen UVA radiation [76]. A dye is therefore not merely a passive component responsible for fiber appearance, but may also modify the degradation rate of the polymer itself.

A similar effect was observed for PAN-based fibers of different colors. After the same exposure, blue fibers showed more pronounced surface changes than orange fibers, which was attributed to differences in dye or additive composition [94]. Two fibers made from the same polymer but dyed with different substances may therefore age at different rates. Degradation may also alter the color of a trace to such an extent that comparison based solely on appearance or colorimetric parameters could lead to the incorrect exclusion of a common origin.

### 7.4. Mechanical Property Deterioration

Deterioration of mechanical properties is the macroscopic consequence of changes occurring at the molecular and morphological levels. Depending on the stage of degradation, it may involve decreases in tensile strength, elongation at break, fatigue resistance, and impact strength, as well as changes in elastic modulus and hardness. These parameters may change in different directions. An increase in stiffness or hardness should therefore not be interpreted as improved durability.

In polyamides, initial exposure to water causes plasticization. Water molecules weaken some of the interactions between polymer chains and increase their mobility, resulting in lower elastic modulus and strength and greater deformability. Changes caused solely by plasticization are at least partly reversible after water removal. Prolonged exposure or elevated temperatures, however, promote hydrolysis of amide bonds and a decrease in molecular weight. Although hydrolysis remains in equilibrium with condensation between amino and carboxyl groups, this does not usually fully restore the original chain length, entanglement network, or material morphology. As a result, the polyamide becomes less ductile and more likely to fracture without substantial prior deformation [27,75,111].

The transition to brittle behavior may occur after a relatively small degree of chemical transformation. In PA66, elongation at break decreased sharply once the molecular weight fell below a critical value. Below this threshold, the chains were too short to maintain a continuous entanglement network [75,78]. Chemicrystallization reduces the proportion of the amorphous phase capable of deformation. Increased crystallinity may therefore raise local hardness while limiting deformability, making the fiber more brittle and more susceptible to fracture [86].

Oxidation and photodegradation also impair the mechanical properties of polyamides. Chain scission reduces strength and elongation at break, whereas crosslinking may locally increase stiffness. When degradation is concentrated in the surface layer, cracks and brittle regions formed there may initiate further damage. The fiber consequently becomes more susceptible to breakage and fragmentation during bending, abrasion, or other mechanical loading [78,85]. During exposure to 30% H_2_O_2_, PA66 showed a significant decrease in tensile strength and elongation at break, indicating pronounced mechanical degradation, whereas the properties of acrylic fibers remained much more stable [91].

The mechanical properties of PAN depend on which of the competing processes predominates. Scission of the main polymer chain during intense photodegradation reduces strength and increases susceptibility to fragmentation. Hydrolysis, in contrast, alters the functional-group composition of PAN and, under more severe conditions, may also weaken the material structure. Cyclization and crosslinking during heating may increase stiffness and thermal resistance but simultaneously reduce fiber deformability. Excessive stabilization therefore produces a stiff and brittle material. If these transformations occur non-uniformly across the fiber cross-section, the more highly crosslinked outer layer has a higher modulus than the core. This difference promotes internal stresses and cracking [98].

Under milder exposure conditions, the mechanical properties of acrylic fibers may remain relatively stable even when minor surface or chemical changes occur. In studies involving KOH, sodium hypochlorite, H_2_O_2_, and Fenton’s reagent, the tensile strength and elongation at break of PAN-based fibers changed only slightly compared with those observed for other fibers [91]. Under more severe hydrolytic conditions, substantial changes in chemical composition and surface morphology may occur, whereas intense irradiation may lead to cracking, loss of structural continuity, and fiber fragmentation [103,105].

Progressive mechanical degradation therefore leads to reduced strength, lower deformability, and greater susceptibility to fiber breakage and fragmentation. The extent of these changes depends on the dominant degradation mechanism, the degree of chain shortening, changes in crystallinity, and any crosslinking that occurs.

## 8. Forensic Analytical Techniques for the Characterization of Textile Fibers

Based on the available literature, the algorithm for the identification and comparative analysis of fibers for forensic purposes comprises several stages focused on the examination of fiber morphology, fiber-forming polymers, as well as dyes and additives present in the fibers. To achieve this, a combination of complementary analytical techniques is employed, including (1) microscopic examination using optical microscopy and scanning electron microscopy (SEM) to assess morphological and surface characteristics; (2) spectroscopic methods, such as Fourier transform infrared spectroscopy (FTIR), Raman spectroscopy, and UV–Vis microspectrophotometry (MSP UV–Vis), for the identification of polymer composition and color characteristics; (3) thermal analysis techniques, including differential scanning calorimetry (DSC) and thermogravimetric analysis (TGA), to evaluate thermal properties and material composition; and (4) chromatographic and mass spectrometric techniques, including high-performance liquid chromatography (HPLC) with various detectors for the characterization of dyes, additives, and other chemical constituents, as well as pyrolysis–gas chromatography/mass spectrometry (Py-GC/MS) for the identification of fiber-forming polymers. The individual techniques provide complementary information and differ substantially in terms of analytical targets, sample requirements, degree of sample consumption, and discriminatory power. Their selection should therefore be based on the characteristics of the evidential material and the specific analytical question [23,112,113,114,115,116,117,118,119].

### 8.1. Microscopy (Optical Microscopy, SEM)

Optical microscopy constitutes a fundamental tool in forensic fiber examination, providing valuable information on the morphological and physicochemical characteristics of fibers. These methods enable the preliminary identification, classification, and comparison of questioned and reference samples. Owing to their non-destructive nature, optical microscopy methods are typically applied in the early stages of the analytical workflow [20,23,113,120,121].

Several microscopic techniques are commonly used in forensic fiber examinations. *Stereoscopic microscopy* (reflected-light microscopy) allows the observation of textiles/fibers at relatively low magnifications and provides information on their color, surface appearance, texture, general morphology, and structural characteristics. Materials made from polyamide and polyacrylonitrile fibers belong to the group of synthetic materials. PA and PAN fibers exhibit characteristic morphological features that facilitate their preliminary differentiation. Under a stereomicroscope, polyamide textiles typically exhibit a regular woven or knitted structure composed of smooth, uniform fibers with a consistent diameter. The surface of the material is homogeneous and slightly glossy. Materials made from polyacrylonitrile are characterized by a bulkier, fluffier structure. Under a stereomicroscope, thin, smooth fibers of similar diameter are visible, often showing slight crimping, which gives the material greater bulk. The surface of the material is typically uniform and matte. This technique is particularly useful during the initial screening and selection of fibers recovered from crime scenes [1,113,122,123].

*Biological microscopy (transmitted-light microscopy)* is used to examine the internal structure and transparency of fibers. It enables the assessment of characteristics such as diameter, longitudinal features, surface irregularities, cross-sectional shape, and structural damage. These parameters can assist in distinguishing between natural and synthetic fibers and in identifying manufacturing characteristics. A single polyamide fiber, when observed under a microscope, is characterized by a smooth and uniform surface. In the case of profiled fibers, longitudinal grooves running along the fiber axis are visible. The cross-section of conventional polyamide fibers is typically oval or nearly circular, whereas profiled fibers may exhibit a triangular cross-section with rounded corners. A single polyacrylonitrile fiber, when observed microscopically, is also characterized by a smooth and uniform surface. Profiled fibers may display longitudinal grooves extending along the fiber axis. The cross-sectional shape depends on the spinning technology and manufacturing process. It is most commonly kidney-shaped or dog-bone-shaped, although other cross-sectional geometries may also occur [113,120,121,123,124,125,126].

*Polarized light microscopy (PLM)* is one of the most important techniques for forensic fiber identification. By exploiting the optical anisotropy of fibers, PLM provides information on refractive index, birefringence, extinction behavior, and optical sign. These properties are highly characteristic of specific fiber types and can therefore be used to differentiate between fibers with similar visual appearances but different chemical compositions. Polyamide fibers exhibit relatively high birefringence when observed under a polarizing-light microscope. Between crossed polarizers, these fibers display strong optical anisotropy and intense interference colors. Due to their high birefringence, polyamide fibers typically show vivid interference colors, including bright shades of red, blue, green, and yellow. Polyacrylonitrile fibers observed under a polarized light microscope exhibit very low birefringence. Under crossed polarizers, the fibers do not exhibit intense interference colors. Instead, they display weak, dark gray, steel gray, or milky white interference colors [1,20,113,127,128,129,130].

*Fluorescence microscopy*, which utilizes ultraviolet (UV) radiation, can reveal differences in the fluorescence behavior of fibers and dyes. Certain natural and synthetic fibers exhibit characteristic fluorescence patterns, whereas textile dyes may fluoresce differently depending on their chemical structure. Consequently, fluorescence microscopy serves as a useful complementary method for distinguishing between fibers that appear similar. Under the microscope, polyamide and polyacrylonitrile fibers differ significantly in terms of fluorescence morphology, edge shape, and background intensity. These differences stem directly from the manufacturing processes of both materials and their unique geometries. Fluorescence characteristics may also be influenced by the composition of the fiber, dyes, finishing agents, and other material constituents [113,124,131,132].

In addition to optical microscopy, *scanning electron microscopy (SEM)* is frequently employed for detailed morphological characterization. SEM provides high-resolution images of fiber surfaces, allowing the examination of fine structural details that cannot be observed using conventional optical microscopy. The technique is particularly valuable for studying surface damage, wear patterns, fractures, manufacturing defects, and other structural features. Furthermore, when combined with *energy-dispersive X-ray spectroscopy (EDS)*, SEM enables the determination of elemental composition, facilitating the identification of inorganic pigments, fillers, and contaminants associated with textile fibers [113,133,134,135,136,137]. Despite its advantages, SEM has certain limitations. The images obtained are typically grayscale, providing no direct information about fiber color or dye composition. Consequently, SEM is not suitable for the examination of textile dyes, which require complementary analytical techniques such as UV–Vis microspectrophotometry (UV–Vis MSP), Raman spectroscopy, or chromatographic methods.

### 8.2. Spectroscopic Methods

Spectroscopic techniques constitute a fundamental component of forensic fiber examination. These methods are generally non-destructive, require minimal sample preparation, and allow the analysis of single fibers recovered as trace evidence, which is particularly important in forensic science. The combined application of FTIR, Raman spectroscopy, and UV–Vis MSP provides comprehensive information about dyes, fiber-forming polymers, and fiber-dye mixtures. The results depend on the spectroscopic technique used (Raman spectroscopy, UV–Vis MSP, or FTIR) and on the analytical conditions [23,113,138,139,140,141].

*Fourier transform infrared spectroscopy (FTIR)* is one of the most widely applied techniques for fiber identification. The method is based on the absorption of infrared radiation by molecular bonds, producing characteristic spectra that serve as molecular fingerprints of fiber-forming polymers. FTIR enables the differentiation of natural fibers, such as cotton, wool, and silk, from synthetic materials including polyester, polyamide, acrylic, and polypropylene. In forensic investigations, FTIR is routinely used to identify polymer composition and to compare questioned fibers with reference samples [23,113,138,139,142].

*Infrared microspectroscopy* is widely regarded as a standard technique for determining the polymer composition of textile fibers. However, its application to the direct identification of fiber dyes is generally limited. This limitation is mainly due to the relatively low sensitivity of infrared absorption to minor components present at concentrations below approximately 5% of the sample mass. Since dyes in textile fibers are typically present at very low concentrations, their detection by conventional infrared spectroscopy is often not feasible. As a result, dye-related spectral contributions are usually weak or obscured by the dominant signal of the fiber polymer, which restricts reliable dye identification in most cases [138,139,142].

Analysis using *Fourier transform infrared spectroscopy (FTIR) and infrared microspectroscopy* allows the differentiation of polyamide fibers from polyacrylonitrile fibers based on the presence of characteristic functional groups in their chemical structure. FTIR can be complemented by other analytical techniques when additional information on fiber characteristics is required [127,139,143,144].

*Raman spectroscopy* provides complementary molecular information based on the inelastic scattering of monochromatic laser light. The technique is particularly useful for the analysis of colored fibers, as it allows the simultaneous characterization of both polymer structure and dye composition. Raman spectroscopy offers high spatial resolution and can be applied directly to individual fibers with little or no sample preparation. Moreover, it is especially valuable for the examination of pigments and dyes that may be difficult to characterize using infrared spectroscopy. Consequently, Raman spectroscopy is a valuable complementary technique for distinguishing between synthetic textile fibers such as polyamide and polyacrylonitrile while simultaneously providing information on their coloration. This method is particularly advantageous for the analysis of individual dyed fibers when it is necessary to determine the polymer composition and identify dye-related components at the same time [23,113,138,140,141,142,145,146,147,148].

*UV–Vis microspectrophotometry (UV–Vis MSP)* combines optical microscopy with spectroscopic analysis in the ultraviolet and visible light ranges, enabling the objective and highly discriminative characterization of the color properties of individual textile fibers. This technique records the absorption spectrum of a single fiber across the ultraviolet and visible light ranges, providing information on the type, chemical structure, and relative concentration of the dyes and pigments responsible for the fiber’s coloration. The technique is particularly useful for examining dyed synthetic fibers, including polyamide and polyacrylonitrile fibers. Although UV–Vis MSP provides valuable information regarding the chromophoric components of dyed fibers, it does not allow direct identification of the polymer composition of the fiber matrix. Therefore, reliably distinguishing between polyamide and polyacrylonitrile fibers requires the use of complementary analytical techniques, such as FTIR or Raman spectroscopy. Combining UV–Vis MSP with vibrational spectroscopy techniques enables a comprehensive characterization of synthetic textile fibers, encompassing both polymer identification and color comparison. Consequently, this integrated analytical approach is considered one of the most important methods for the forensic comparison of dyed textile fibers [23,65,113,130,149,150,151,152,153].

*Surface-enhanced Raman spectroscopy (SERS)* and *surface-enhanced resonance Raman scattering (SERRS)* are techniques that increase the sensitivity of Raman spectroscopy. However, despite the growing number of studies demonstrating the applicability of SERS and SERRS to the analysis of dyes and textile materials, reports describing their routine use in forensic fiber examinations remain limited [140,141,154,155,156,157,158,159,160,161].

### 8.3. Thermal Analysis (DSC, TGA)

Thermal analysis techniques provide valuable information about the physicochemical properties of textile fibers. Among the most commonly applied techniques are differential scanning calorimetry (DSC) and thermogravimetric analysis (TGA), both of which enable the characterization of fiber-forming polymers based on their thermal behavior. In forensic fiber examinations, thermal analysis is primarily used as a supplementary technique when microscopic and spectroscopic methods do not provide sufficient discrimination. Differences in melting behavior, crystallinity, and thermal stability may reveal variations in polymer composition, manufacturing processes, textile treatments, and material characteristics. Consequently, DSC and TGA contribute to the comprehensive characterization of textile fibers and enhance the evidential value of comparative fiber analyses [23,113,136,162,163,164,165,166].

*Differential scanning calorimetry (DSC)* measures the heat flow associated with thermal transitions occurring in a material during controlled heating or cooling. The technique allows the determination of characteristic parameters such as melting temperature (Tm), glass transition temperature (Tg), crystallization behavior, and the degree of crystallinity. Since different fiber-forming polymers exhibit distinct thermal transitions, DSC can be used to differentiate between fiber types and to assess the effects of manufacturing processes and material characteristics. In forensic applications, DSC serves as a supplementary identification tool, particularly when fibers display similar morphological and spectroscopic characteristics [23,162,163,164,166].

*Thermogravimetric analysis (TGA)* measures changes in sample mass as a function of temperature or time under a controlled atmosphere. The technique provides information on thermal stability, decomposition patterns, moisture content, and the presence of additives, fillers, or surface finishes. The thermal degradation profiles obtained by TGA are characteristic of specific polymer classes and may therefore support the identification and discrimination of textile fibers. Furthermore, TGA can reveal differences between fibers that are compositionally similar but differ in manufacturing treatments or additive content [23,162,163,164,165].

Polyamide and polyacrylonitrile fibers exhibit distinct thermal characteristics resulting from their different chemical structures. The characteristic thermal responses of PA and PAN fibers provide additional information useful for their identification and comparison. Consequently, DSC and TGA serve as valuable complementary techniques for the analysis of synthetic textile fibers, particularly in forensic investigations, where microscopic and spectroscopic techniques alone may not provide sufficient discriminatory power. The combined application of DSC and TGA enables comprehensive thermal characterization of textile fibers and provides additional discriminatory information beyond that obtained through microscopic and spectroscopic examinations [23,162,163,164,165,166].

### 8.4. Chromatographic and Mass Spectrometric Techniques

Chromatographic techniques play a complementary role in the forensic examination of synthetic textile fibers. Unlike spectroscopic methods, which are routinely used for polymer identification, chromatographic methods are primarily applied to the analysis of fiber-forming polymers, dyes, pigments, fiber additives, finishing agents, and other chemical constituents.

**Figure 13:**
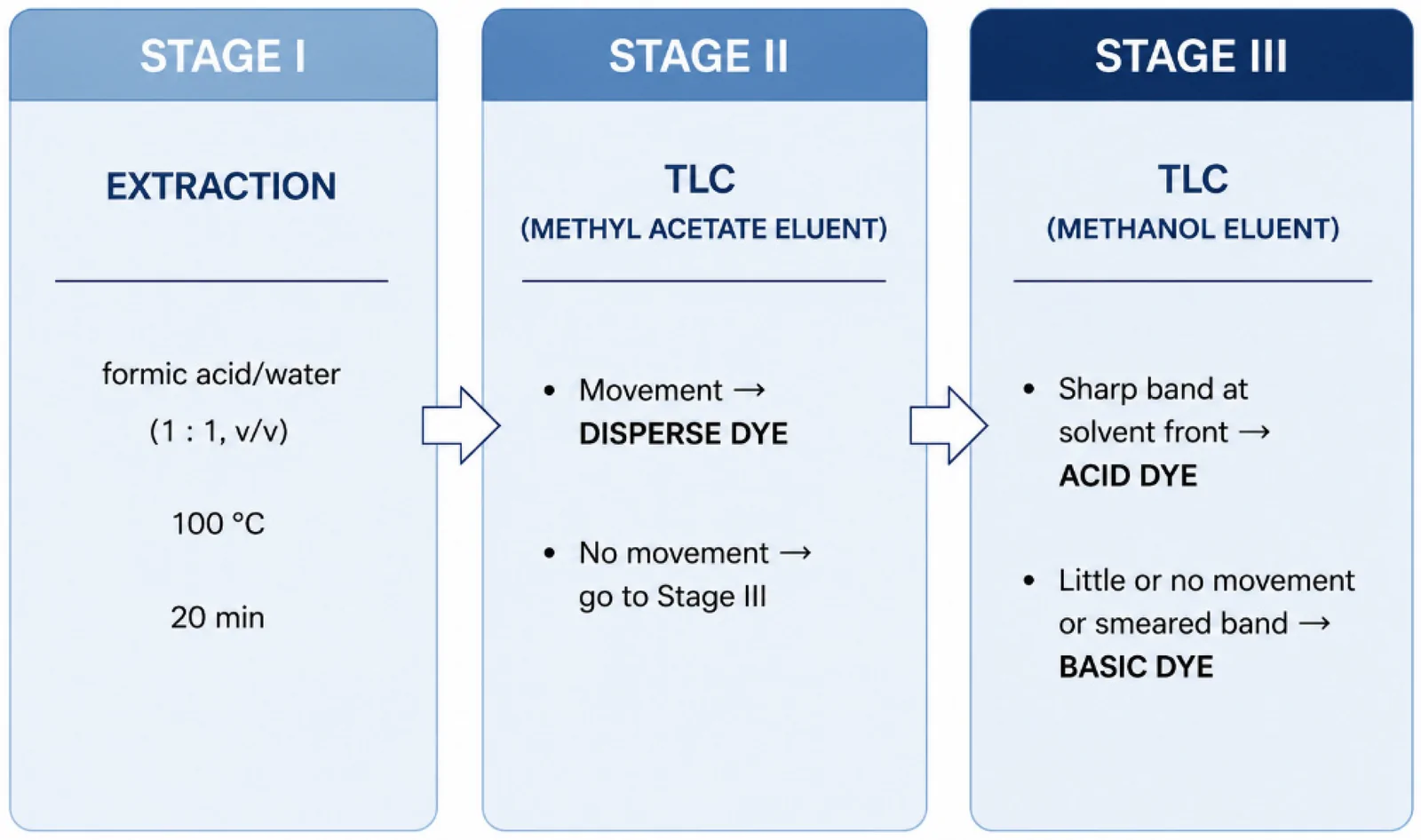
Scheme for the identification of dyes extracted from polyamide fiber based on [167].

**Figure 14:**
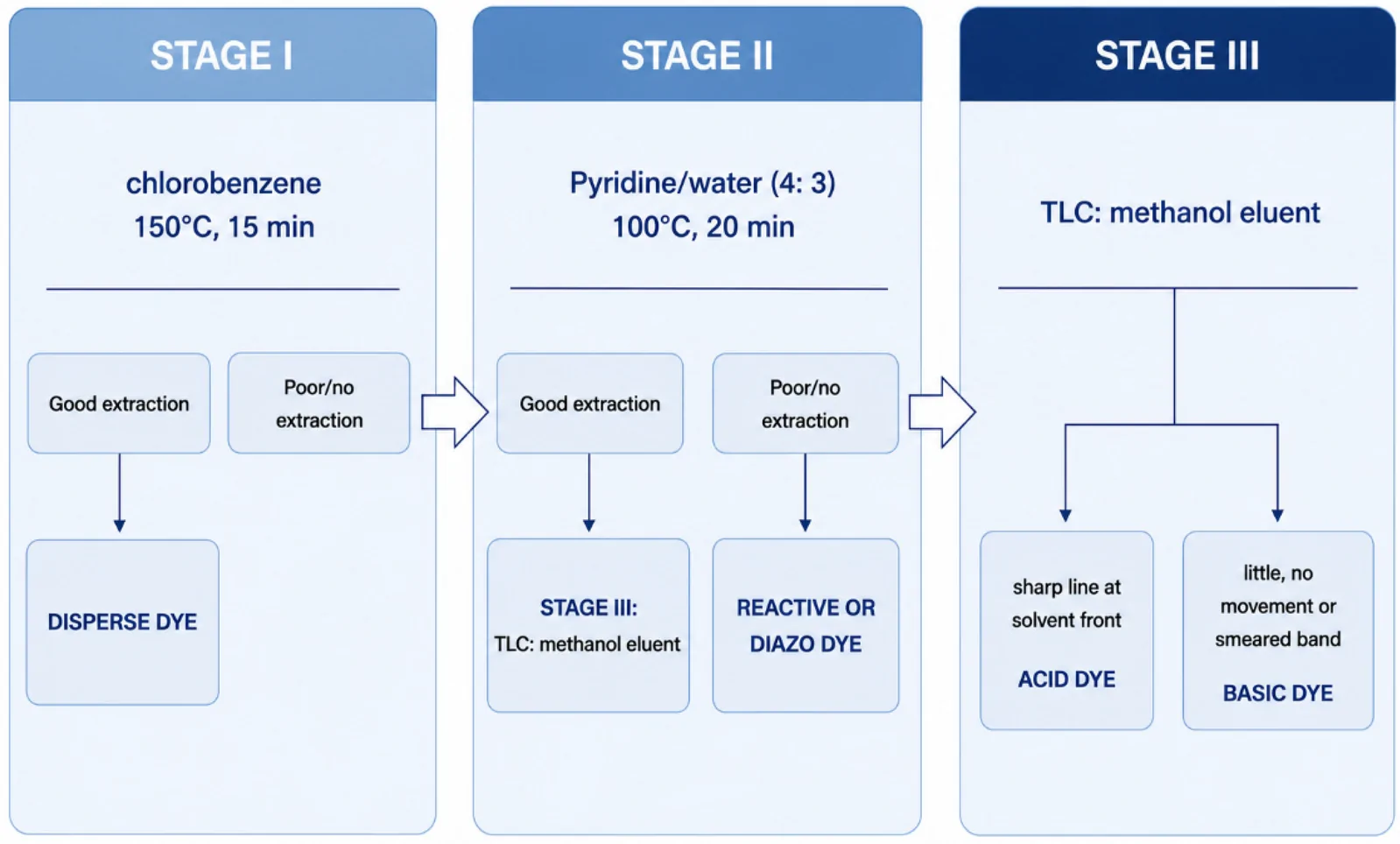
Scheme for the identification of dyes extracted from acrylic fibers based on [167].

Historically, *thin-layer chromatography (TLC)* was one of the first chromatographic techniques introduced into forensic fiber examinations for the analysis of textile dyes. Following solvent extraction, dye components were separated according to their physicochemical properties, enabling comparative analysis of dye compositions in questioned and reference fibers [12,23,167,168,169,170]. The separated dye components produce characteristic chromatographic patterns that can be compared between questioned and reference fibers. Owing to its simplicity, rapid analysis, and low cost, thin-layer chromatography (TLC) has been widely used as a screening and comparative method for dye analysis. The first step in dye identification is the extraction of dyes from the fiber. A variety of extraction procedures have been described in the literature, including different extraction schemes designed to facilitate the classification and comparison of textile dyes, depending on the chemical class of the colorants and their affinity for the fiber-forming polymer [12,23,150,167,171]. Extraction procedures for polyamide (Figure 13) and polyacrylonitrile (Figure 14) fibers are presented below.

However, its relatively low sensitivity and limited resolving power restrict its ability to distinguish complex dye mixtures and to detect the minute quantities of dye present in single fibers [12,23,167,169].

As a result, thin-layer chromatography (TLC) has largely been replaced by high-performance liquid chromatography (HPLC), although it remains a useful complementary technique in some forensic laboratories.

High-performance liquid chromatography (HPLC) is currently the most widely used chromatographic technique for the forensic analysis of textile dyes. It enables the separation and comparison of dyes extracted from textile fibers, yielding characteristic chromatographic profiles that facilitate the differentiation of fibers with similar visual appearances [12,23,150,167]. To enhance sensitivity and selectivity and improve the effectiveness of dye identification, HPLC is often coupled with various detectors, including UV–Vis, diode-array detection (DAD), mass spectrometry (MS), tandem mass spectrometry (MS/MS), and high-resolution mass spectrometry (HRMS) [116,118,172,173].

Chromatographic methods are also widely used to analyze dyes extracted from synthetic fibers. Although the available literature contains relatively few studies specifically addressing polyamide and polyacrylonitrile fibers, analytical strategies successfully applied to polyester and cotton fibers can also be adapted for the examination of these materials [12,116,118,167,172,173].

Polyamide and polyacrylonitrile fibers can be characterized using chromatographic approaches, particularly when combined with mass spectrometric detection. Due to the high molecular weight and limited volatility of intact polymer chains, direct chromatographic analysis of textile fibers is generally not possible without prior treatment. Therefore, methods such as solvent extraction, hydrolysis, derivatization, or pyrolysis are commonly employed to convert polymeric materials into detectable compounds [23,133,134].

Pyrolysis–gas chromatography/mass spectrometry (Py-GC/MS) is one of the most informative techniques for the characterization of synthetic fiber-forming polymers. During pyrolysis, the polymer undergoes controlled thermal decomposition, generating volatile products that are subsequently separated by gas chromatography and identified by mass spectrometry. The resulting pyrogram provides a characteristic chemical fingerprint of the polymer. Differences in polymer or copolymer composition produce distinct pyrolysis products and pyrograms, enabling the identification and differentiation of polyamide, polyacrylonitrile, and other visually similar synthetic fibers recovered as trace evidence [23,133,134].

Chromatographic techniques are inherently destructive, as they generally require either the extraction of dyes from textile fibers or the thermal decomposition of the fiber-forming polymer. Despite this limitation, they provide highly detailed chemical information and significantly enhance the discriminatory power of forensic fiber analysis. Dye identification and comparison can be based on chromatographic retention times and, when chromatographic separation is coupled with mass spectrometry, on molecular masses and characteristic mass-to-charge (*m*/*z*) ratios of ions. In combination with microscopy, FTIR, Raman spectroscopy, and thermal analysis, chromatographic and mass spectrometric methods therefore contribute to a comprehensive assessment of synthetic textile fibers, providing complementary information on morphology, polymer composition, thermal properties, dyes, additives, and other chemical constituents [12,23,116,117,118,133,167,174].

In summary, the first stage of forensic fiber analysis comprises microscopic examinations. Following this step, appropriate methods—such as spectroscopic and/or chromatographic techniques—must be selected for further analysis of the material. The choice of methodology generally depends on several factors. Consideration must be given to the circumstances of the criminal incident, the form and quantity of the evidence, the type of textile product, the type of dyes, and other relevant factors.

A comparative assessment of the analytical techniques used in forensic fiber examination, including their sample requirements, information obtained, advantages, limitations, and forensic role, is presented in Table 4.

No single analytical technique can be considered universally optimal for forensic fiber identification. Optical microscopy remains the preferred initial screening approach, whereas FTIR is particularly suitable for the identification of the fiber-forming polymer. UV–Vis MSP is highly valuable for objective color comparison, while Raman spectroscopy provides complementary information on both the polymer and the colorant. SEM is particularly useful for assessing surface morphology, whereas DSC and TGA provide supplementary information on thermal properties and structural changes. Consequently, a sequential multi-technique approach, prioritizing non-destructive methods before destructive analyses, provides the most reliable strategy for the forensic examination of textile fibers.

## 9. Analytical Approaches Used for Analysis of Degraded Fibers

Scientific research on degraded textile fibers has advanced considerably in recent decades. Modern analytical techniques enable the investigation of degradation processes at the microscopic, molecular, and elemental levels, providing detailed insight into the mechanisms responsible for changes in fiber morphology, structure, and chemical composition. Understanding these degradation pathways is essential not only for identifying the original textile material but also for reconstructing the conditions under which degradation occurred and, where possible, identifying the degrading agent responsible.

In contrast to Section 8, which focuses on the analytical capabilities, advantages, limitations, and forensic roles of individual techniques, this section focuses on their application to specific degradation processes and on the interpretation of the resulting morphological, physicochemical, and chemical changes. The following subsections discuss this.

### 9.1. Analysis of Environmentally Degraded Fibers

Rubežienė et al. (2012) reviewed the effect of light exposure on the durability of textile materials and discussed previous studies on the photodegradation of synthetic fibers [175]. The authors emphasized that polyamide fibers are particularly susceptible to ultraviolet radiation despite the application of photostabilizers during fiber production. According to the cited literature, titanium dioxide, commonly used as a delustrant, accelerates photolytic degradation by promoting polymer chain scission, resulting in yellowing, reduced tensile strength, and a decrease in the degree of polymerization [136,144,175,176]. However, both cited publications lack detailed data regarding the research material, analytical methods, and experimental conditions.

Thomas and Hridayanathan (2006) investigated the effects of natural solar radiation on four types of polyamide monofilaments and four types of polyamide multifilament twines [176]. Test specimens were mounted without tension on aluminum nails spaced 1 cm apart on a rectangular wooden frame designed to ensure adequate ventilation and prevent excessive heat accumulation. The frame was positioned in a north–south orientation at a 45° angle on a rooftop, allowing continuous direct exposure to sunlight throughout the day. The experiment was conducted on Willingdon Island, Cochin (India), under marine atmospheric conditions. Solar radiation was monitored monthly at midday using a solar collector. Samples were collected after 8, 15, 30, 45, 60, 90, 120, 150, and 180 days of exposure to determine changes in tensile strength and elongation at break. During the exposure period, the average monthly temperature ranged from 30.6 to 32.7 °C, while monthly precipitation ranged from 0 to 503 mm. Exposed and unexposed (control) specimens were tested using a universal testing machine. Five replicates of each sample were analyzed at every sampling interval, and the tensile strength and elongation at break were determined [176]. The study demonstrated that UV-induced photooxidation significantly reduced the mechanical properties of polyamide fibers, with the extent of degradation depending on both the duration of exposure and fiber morphology. Multifilament and finer polyamide fibers were found to be more susceptible to photodegradation than monofilament and coarser fibers because of their higher specific surface area and the greater penetration of ultraviolet radiation [175,176]. Moreover, prolonged UV exposure may lead to a substantial reduction in tensile strength, in some cases approaching complete strength loss after extended irradiation [176,177]. These findings highlight the importance of considering photo-induced ageing when interpreting weathered polyamide fibers in forensic examinations [23,178].

Mujumdar et al. (2019) investigated the effects of outdoor weathering on dyed textile fabrics, including acrylic 864, nylon 361, and cotton 400, dyed with Basic Green 4, Acid Yellow 17, and Direct Blue 1 [179]. The textile samples were exposed to natural environmental conditions in two contrasting climatic regions of the United States: a desert environment in Arizona and a humid environment in Florida. The exposure periods were 0, 3, 6, 9, and 12 months. After each three-month interval, ten fiber samples were collected from each fabric specimen for analysis. The samples were examined using fluorescence microscopy, and fluorescence data were acquired as two-dimensional excitation and fluorescence spectra (2D spectra) and three-dimensional (3D) excitation–emission matrices (EEMs). The experimental results demonstrated a significant decrease in fluorescence intensity following outdoor weathering. A multivariate chemometric approach based on discriminant unfolded partial least squares (DU-PLS) was subsequently applied to analyze the fluorescence datasets and evaluate the discrimination of weathered textile fibers [179].

Comparison of excitation–emission matrix (EEM) fluorescence maps with two-dimensional fluorescence emission spectra demonstrated that dye photodegradation occurred rapidly during the first three months of outdoor weathering, followed by a slower rate of degradation over the remaining exposure period. The degradation mechanism was strongly influenced by the environmental conditions. Different polymer types exhibited markedly different susceptibilities to outdoor weathering. Among the investigated materials, nylon fibers showed pronounced sensitivity to both hot–dry (Arizona) and warm–humid (Florida) climatic conditions, whereas acrylic fibers displayed considerably greater environmental stability and were less susceptible to photodegradation [179].

The DU-PLS model successfully differentiated unexposed nylon fibers from those subjected to outdoor weathering and accurately classified fibers according to both the climatic environment and the duration of exposure. The algorithm was capable of distinguishing nylon fibers exposed to Arizona and Florida conditions, as well as discriminating between different weathering periods within each environment. These findings demonstrate the potential of combining fluorescence spectroscopy with chemometric analysis to reconstruct the environmental exposure history of polyamide fibers in forensic investigations [179].

In the chapter “Identification and Analysis of Textile Damage” of the book *Textile Fiber Identification*, Schindler summarizes the available literature published between 1959 and 2002 concerning the degradation of synthetic fibers, including polyamide and polyacrylonitrile [136]. The author emphasizes that the susceptibility of these fibers to environmental degradation is governed primarily by their polymer structure, the presence of stabilizing additives, and the conditions of exposure. Prolonged exposure to ultraviolet (UV) radiation initiates photooxidative degradation, leading to polymer chain scission, discoloration, embrittlement, and the gradual deterioration of the mechanical properties of synthetic fibers [136,175]. These physicochemical changes may significantly affect both the morphology and the evidential value of textile microtraces recovered during forensic investigations [23,178]. The extent of photodegradation is influenced by several interacting factors, including exposure time, fiber diameter, polymer crystallinity, and the presence of delustrants or inorganic fillers. In particular, titanium dioxide (TiO_2_), widely used as a delustrant in synthetic fibers, may accelerate photooxidative degradation by increasing internal light scattering and promoting the formation of reactive oxygen species within the polymer matrix [136,175]. As degradation progresses, changes in surface morphology, optical properties, tensile strength, and elasticity become increasingly pronounced, potentially complicating the forensic comparison and identification of degraded textile fibers [23,124,178].

Adetimehin et al. (2025) studied the effects of animal (pig) decomposition on clothing materials under outdoor environmental conditions [35]. The authors conducted a long-term (over three years) macroscopic and microscopic qualitative assessment of textile degradation, including garments made of polyacrylonitrile (acrylic) fibers, placed in contact with decomposing pig carcasses. The carcasses were left to decompose naturally over several seasons in the dense Cape Flats Dune Strandveld vegetation, representing a forensically relevant environment in the Cape Town region. The results demonstrated that acrylic fibers remained largely intact at both the microscopic and macroscopic levels after nearly three years of exposure, although considerable distortion of the fabric weave was observed. These findings indicate that garments made of polyacrylonitrile fibers undergo characteristic structural changes during prolonged contact with decomposing bodies while retaining the integrity of individual fibers. Such degradation patterns may provide useful forensic information and may contribute to estimating the minimum postmortem interval (PMI) in cases involving clothed human remains [35].

### 9.2. Analysis of High-Temperature-Degraded Fibers

Research conducted by Wąs (1997) and subsequently by Wąs and Włochowicz (1997) focused on the characterization of textile fibers exposed to elevated temperatures and on evaluating the feasibility of identifying the residues produced during their thermal degradation and combustion [127,180]. Approximately 30 types of textile fibers, including polyamide and polyacrylonitrile, were examined. The analyzed fibers contained neither dyes nor finishing agents nor other chemical additives introduced during the manufacturing process, with titanium dioxide being the only additive used as a delustrant. Thermal degradation was simulated by melting the fibers in a heating cell (up to 300 °C), burning them in a gas flame, and completely incinerating them in a muffle furnace (up to 800 °C). The resulting materials were analyzed using Fourier transform infrared (FTIR) microspectroscopy, scanning electron microscopy coupled with energy-dispersive X-ray spectroscopy (SEM-EDS), petrographic microscopy, and X-ray powder diffraction (XRPD). The results demonstrated that the combined application of these analytical techniques enables reliable identification and differentiation of thermally degraded and incinerated textile fibers in forensic examinations [127,180].

In subsequent studies, Wąs-Gubała (2009) and Wąs-Gubała and Krauß (2004a, 2004b, 2006) investigated morphological changes in textile products subjected to different thermal factors, including the shock wave accompanying a vapor cloud explosion (VCE), direct flame exposure, and contact with a heated flat metal surface [124,181,182,183]. These thermal conditions simulate damage that may occur during explosions, fires, arson incidents, and road traffic accidents [124,181,182,183]. Changes in textile products and individual fibers were evaluated using optical and scanning electron microscopy. Polyacrylonitrile textiles exposed to a VCE exhibited damage primarily to the outer yarn layers, whereas exposure to direct flame or a heated metal plate resulted in extensive destruction of the fabric structure. At the fiber level, VCE exposure caused thermal modification mainly at the fiber ends. Contact with a heated surface produced extensive melting and flattening of fiber ends, while flame exposure resulted in characteristic swelling and flattening of the fibers. Polyamide fibers exhibited more pronounced melting behavior. Following VCE exposure, individual fiber ends were no longer distinguishable, and numerous voids were observed within the molten polymer. Contact with the heated plate caused rapid melting of the fibers, whereas flame exposure produced characteristic spherical formations both along the fiber length and at the fiber ends (Table 5 and Table 6). The authors concluded that each thermal factor generated distinct degradation patterns characteristic of both the heat source and the fiber type, providing valuable criteria for forensic interpretation [124,181,182,183].

**Figure 15:**
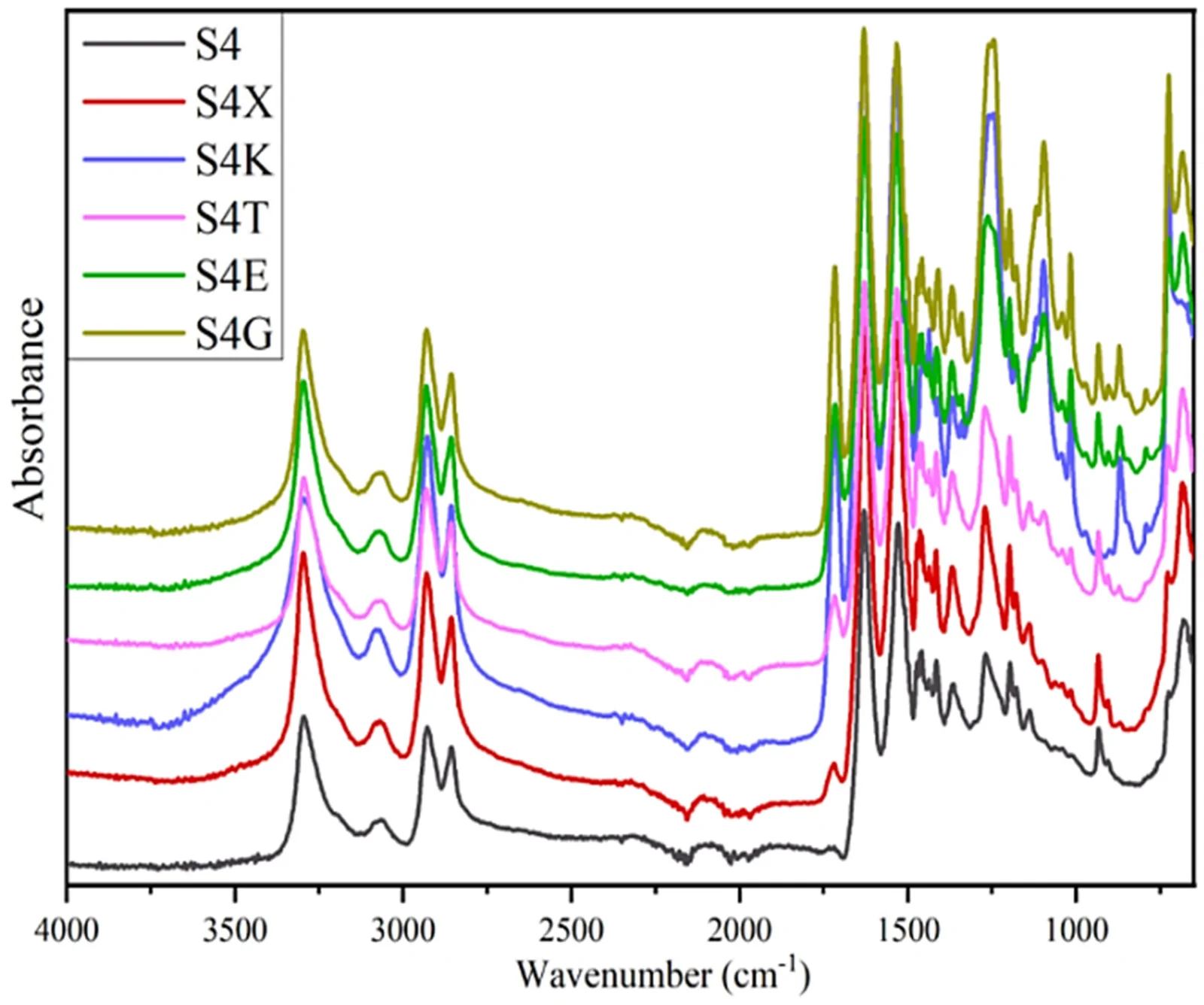
FTIR spectrum of polyamide samples: reference material (S4), burned without accelerant (S4X) and burned with: kerosene (S4K), thinner (S4T), ethyl alcohol (S4E) gasoline (S4G). Reprinted from Kızıl, S.; Yosmaoğlu, B.I.; Atasoy, S. Forensic and analytical profiling of ignitable liquid residues on diverse fabric types. Gümüşhane Üniversitesi Fen Bilimleri Dergisi 2025, 15, 1179–1193 [184], with permission from the publisher Gümüşhane University Journal of Science and Technology.

**Figure 16:**
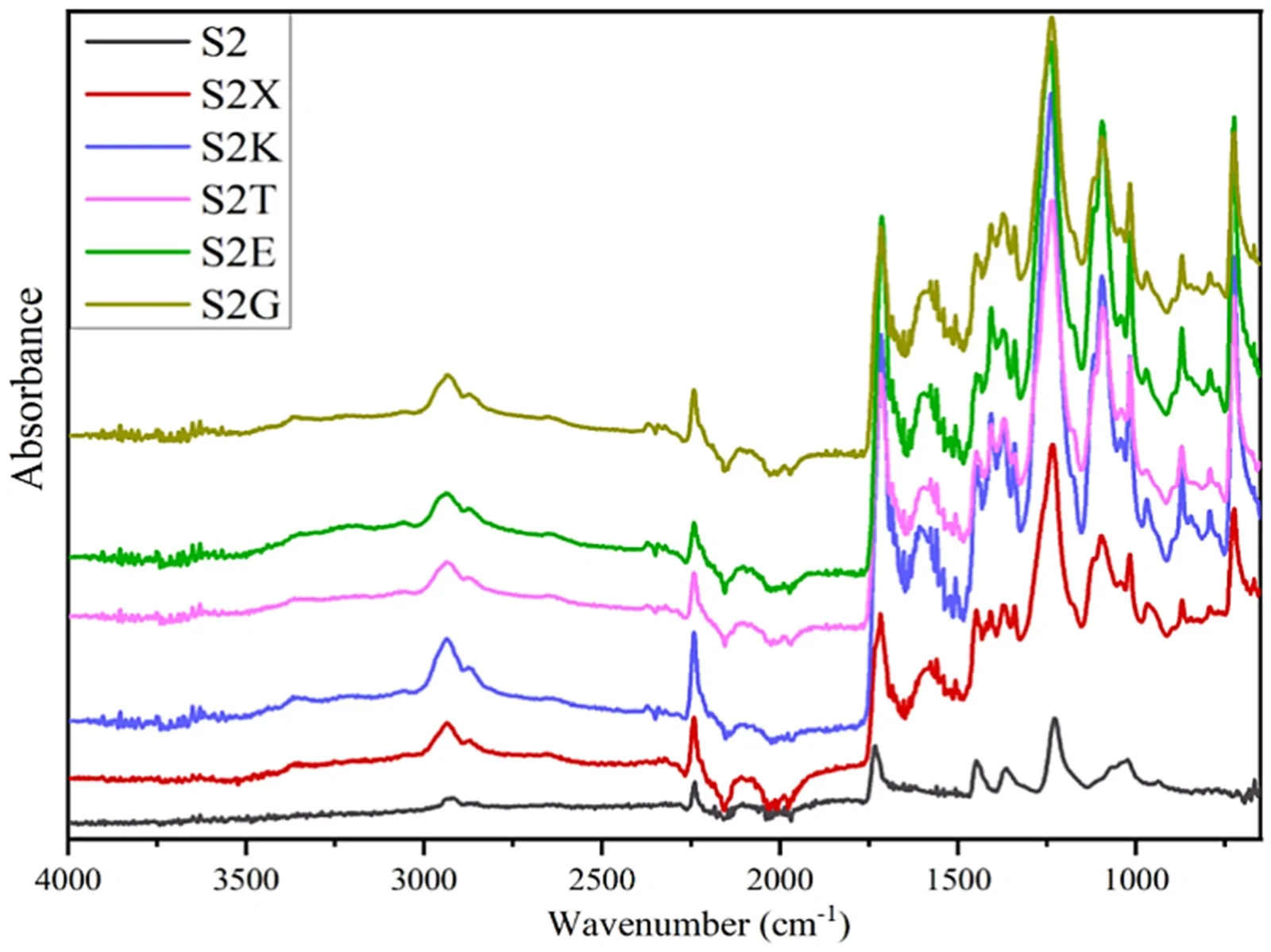
FTIR spectrum of polyacrylic samples: reference material (S2), burned without accelerant (S2X) and burned with: kerosene (S2K), thinner (S2T), ethyl alcohol (S2E) gasoline (S2G). Reprinted from Kızıl, S.; Yosmaoğlu, B.I.; Atasoy, S. Forensic and analytical profiling of ignitable liquid residues on diverse fabric types. Gümüşhane Üniversitesi Fen Bilimleri Dergisi 2025, 15, 1179–1193 [184], with permission from the publisher Gümüşhane University Journal of Science and Technology.

Kizil et al. (2025) evaluated the applicability of Fourier transform infrared spectroscopy (FTIR) combined with principal component analysis (PCA) for identifying ignitable liquids based on the chemical changes observed in burned textile fabrics, including polyacrylonitrile and polyimide materials [184]. Fabric samples were analyzed in their unburned state, after combustion without an accelerant, and following combustion in the presence of four ignitable liquids: gasoline, synthetic thinner, ethanol, and kerosene. During the experiment, each fabric sample was immersed in 50 mL of an ignitable liquid (IL) for 10 s. After immersion, the sample was removed, allowed to drain briefly, placed in a glass Petri dish, and ignited using a lighter. This procedure was performed separately for each combination of fabric type and ignitable liquid. In addition, six fabric types were burned using only a lighter, without the application of an ignitable liquid, and served as control samples. All burned samples were subjected to analytical examination one day after combustion. FTIR analysis revealed characteristic spectral changes associated with each ignitable liquid, indicating the presence of distinct chemical residues on the textile substrates (Figure 15 and Figure 16). Principal component analysis successfully differentiated burned from unburned samples; however, it did not clearly discriminate among the individual ignitable liquids. The authors concluded that FTIR combined with PCA represents a rapid, non-destructive, and complementary analytical approach that can support conventional gas chromatography–mass spectrometry (GC-MS) in forensic fire investigations by facilitating the preliminary detection of ignitable liquid residues [184].

### 9.3. Analysis of Use-Degraded Fibres

Wąs-Gubała (2009) [124] and Wąs-Gubała and Grzesiak (2010) [185] investigated the effects of household laundering on the color stability of polyacrylonitrile fibers. Gray, blue, and red polyacrylonitrile yarn skeins were selected for the study, while an undyed and unfinished polyacrylonitrile fabric served as the reference material. The samples were repeatedly washed over a 14-day period using four powdered detergents and one liquid detergent prepared with either tap or distilled water. Color changes in the textile products and individual fibers were evaluated by visual examination, fluorescence microscopy, UV–Vis microspectrophotometry, and visible-light spectroscopy [124,185].

The study demonstrated that color changes were influenced primarily by the fiber type, dye composition, detergent formulation, and duration of laundering, whereas the quality of the water used to prepare the washing solutions had no significant effect (Table 7 and Table 8). Although only slight color alterations were observed in the polyacrylonitrile fibers and these changes were generally imperceptible to the naked eye, fluorescence microscopy and microspectrophotometry successfully detected subtle differences. The authors concluded that routine laundering may produce measurable alterations in textile fibers that can serve as valuable forensic characteristics during comparative fiber examinations [124,185].

Heider et al. (2016) proposed a non-destructive method for identifying detergents deposited on textile fibers based on the fluorescence analysis of fluorescent whitening agents (FWAs) adsorbed onto fiber surfaces during laundering [186]. The study was conducted using both dyed and undyed nylon (Nylon 361) and acrylic (Acrylic 864) fibers. Material samples were washed using seven commercially available detergents (All, Cheer, Oxiclean, Purex, liquid Tide, powdered Tide, and Wisk). Subsequently, individual fibers were analyzed using fluorescence microscopy coupled with spectrofluorometry. Fluorescence emission spectra were recorded in the 390–660 nm wavelength range, using an excitation wavelength of 350 nm. The spectral data were processed using principal component analysis (PCA), which enabled the classification of fluorescence spectra into distinct clusters corresponding to different detergents. Fibers washed five times were used to establish the training dataset, whereas fibers washed five or six times served as validation samples to evaluate the robustness of the classification model. The authors concluded that the recorded fluorescence spectra were influenced not only by the fluorescent whitening agents but also by interactions between the fiber substrate, textile dyes, and other detergent components. Consequently, detergent-derived fluorescence provides valuable complementary information for forensic fiber comparison. The proposed non-destructive approach has considerable potential for enhancing the evidential value of trace fiber examinations by providing additional information regarding the laundering history and possible origin of textile evidence [186].

### 9.4. Analysis of Chemically Degraded Fibers

Śmigiel-Kamińska (2014) investigated the effects of selected corrosive substances on the morphology of textile products and individual fibers with the aim of assessing their forensic value following chemical degradation [126]. The study included 10 × 10 cm textile specimens of polyamide and polyacrylonitrile knitted fabrics exposed under controlled laboratory conditions to 36% hydrochloric acid, 98% sulfuric acid, 65% nitric acid, sodium hydroxide, and sodium hypochlorite. After the application of 5 mL of each chemical reagent, the samples were left to react for 24 h, and each experiment was performed in triplicate. Morphological alterations were evaluated using optical microscopy (stereoscopic and transmitted-light microscopy) and scanning electron microscopy (SEM) [126]. The results demonstrated that the degree of degradation depended strongly on the type of corrosive agent and the chemical composition of the fibers. Sulfuric acid produced the most severe damage, dissolving both polyamide and polyacrylonitrile textile products. Polyamide fabrics exhibited dissolution of the fibers, leaving fragile, plastic-like edges, whereas polyacrylonitrile textiles were transformed into a yellow, pliable mass containing swollen fiber aggregates that could no longer be separated for microscopic examination. Nitric acid had a less destructive effect on synthetic fibers. Although polyacrylonitrile textile products underwent discoloration and partial transformation into an orange, plastic-like material, the morphology of individual fibers remained largely preserved. Polyamide textiles also exhibited color changes after nitric acid treatment without significant alterations in fiber morphology. In contrast, hydrochloric acid, sodium hydroxide, and sodium hypochlorite produced little or no morphological damage to either polyamide or polyacrylonitrile fibers under the experimental conditions, although crystallized residues of alkaline agents were observed on the fiber surfaces following solvent evaporation [126]. From a forensic perspective, the study demonstrated that severe chemical degradation of synthetic textiles does not necessarily preclude fiber identification. Even when textile products underwent substantial structural deterioration, characteristic morphological features of individual fibers frequently remained sufficiently preserved to permit forensic examination. The findings emphasize the importance of combining stereomicroscopy, transmitted-light microscopy, and scanning electron microscopy when evaluating chemically damaged textile evidence and support a more reliable interpretation of fibers recovered from crime scenes involving corrosive substances [126].

### 9.5. Analysis of Mechanically Damaged Textiles

Mechanical damage is one of the most frequently encountered forms of textile degradation in forensic investigations [72,136]. It may result from cutting, stabbing, slashing, tearing, abrasion, blunt-force impact, projectile penetration, or repeated wear during normal use. Because different damage mechanisms produce characteristic morphological features, the examination of textile damage can provide valuable information regarding the type of force applied, the nature of the damaging object, and the circumstances surrounding an incident [72,136,178].

The morphology of mechanically damaged textiles is influenced by numerous factors, including the geometry and sharpness of the implement, the direction and magnitude of the applied force, the construction of the fabric, and the tension of the garment at the moment of damage [72,136]. Experimental studies have demonstrated that single-edged, double-edged, and serrated blades produce distinctive damage patterns, whereas stab and slash mechanisms generate different fracture morphologies [187,188,189]. Furthermore, the dimensions and appearance of stab damage are affected by whether the clothing is loose or under tension during penetration, emphasizing the importance of considering the circumstances under which the damage occurred [187].

Projectile-induced damage represents another important category of mechanical degradation [72,136]. The morphology of textile defects depends on projectile type, velocity, impact angle, and the structural characteristics of the fabric. Studies investigating air weapon pellets, arrows, and firearm projectiles have demonstrated that different projectile geometries produce characteristic damage patterns and that the presence, composition, and fit of clothing significantly influence both the morphology of textile damage and projectile penetration [137,190,191]. In synthetic textiles, localized melting and fusion of fibers may additionally occur because of the heat generated during firearm discharge [137].

The forensic interpretation of mechanical damage may be further complicated by post-event environmental processes [72,136]. Experimental studies have shown that insect activity associated with decomposition may increase fraying of stab-cut edges, whereas burial conditions influence the rate of textile degradation depending on soil type and the presence of decomposition fluids [192,193,194,195]. In addition, normal wear and tear may produce damage resembling that associated with criminal activity, highlighting the importance of distinguishing everyday deterioration from event-related damage [196,197,198].

Current forensic practice relies primarily on macroscopic examination, optical microscopy, and scanning electron microscopy to characterize mechanical damage [23,72,121]. However, despite considerable advances in experimental studies, textile damage interpretation remains challenging because of the absence of standardized interpretative frameworks and comprehensive reference databases [72,199,200]. Consequently, recent reviews have emphasized the need for systematic analytical protocols, reconstruction experiments, and probabilistic approaches to improve the reliability and objectivity of forensic textile damage interpretation [23,31,72,103,174,200,201,202,203,204,205,206,207,208,209,210,211,212,213].

It is important to recognize that most forensic studies investigating mechanical textile damage have not specifically examined polyamide or polyacrylonitrile fibers but have instead focused on other textile materials, including cotton, wool, polyester, and blended fabrics [72,136]. Although these investigations provide valuable insights into general damage mechanisms and forensic interpretation, direct extrapolation of their findings to polyamide (PA) and polyacrylonitrile (PAN) fibers should be undertaken with caution because each polymer exhibits distinct mechanical properties and fracture behavior [1,136,175]. Nevertheless, in the absence of comprehensive polymer-specific datasets, these studies constitute an important reference for understanding textile damage mechanisms and for developing analytical approaches applicable to synthetic fibers encountered in forensic casework [23,72].

## 10. Case Studies and Practical Applications

### 10.1. Forensic Case Examples

Although the degradation of polyamide and polyacrylonitrile fibers is a phenomenon frequently encountered in forensic investigations, including cases involving fires, explosions, environmental factors, or chemical exposure, publications describing specific criminal cases are rare. The limited availability of such studies stems largely from the confidential nature of forensic laboratory work and legal restrictions regarding the publication of evidentiary materials [174].

In a book published in 2009, Wąs-Gubała presented a case study involving the forensic analysis of a thermally altered knitted fabric made of polyamide fibers. The case concerned the killing of a woman by arson. An analysis of damage to trousers made of PA 6,6 polyamide fibers revealed that the observed melting could have resulted from contact with a surface heated to a temperature of at least 250 °C [124].

Furthermore, the available literature is dominated by several main categories of research on degraded polyamide and polyacrylonitrile fibers. The first category comprises experimental studies of significant forensic importance, aimed at replicating degradation phenomena observed in actual cases. Although conducted under controlled laboratory conditions, these studies were designed to establish interpretative criteria applicable to forensic practice. Another area consists of publications focusing on method validation and interpretative issues rather than on descriptions of specific criminal cases. These studies evaluate the effectiveness of microscopic, spectroscopic, thermal, and chromatographic techniques in identifying degraded polyamide and polyacrylonitrile fibers, as well as in assessing the reliability, reproducibility, and limitations of conclusions drawn by forensic experts. Such validation studies provide the scientific basis for the interpretation of material degradation results by forensic laboratories during legal proceedings [35,126,127,175,176,177,179,180,181,182,183,184,185,186,214].

In summary, the forensic interpretation of polyamide fiber degradation phenomena is based primarily on experimentally proven analytical methodologies, laboratory experience, and internationally accepted interpretative frameworks, rather than on extensive collections of published court case reports.

### 10.2. Evidential Value and Limitations in Court

The evaluation of degradation pathways in polyamide and polyacrylonitrile fibers provides information that extends beyond conventional comparative fiber examination. These synthetic fibers are among the most frequently encountered textile materials in forensic casework due to their widespread use in clothing, carpets, upholstery, ropes, and technical textiles. Exposure to thermal, chemical, photo-oxidative, and biological degrading factors alters their molecular structure, morphology, optical properties, and mechanical performance. Such physicochemical changes may impart distinctive characteristics to otherwise common textile fibers and therefore provide valuable information for reconstructing criminal events, assessing the environmental conditions to which the evidence has been exposed, and evaluating the credibility of witness statements [23,124,215].

Polyamide fibers are particularly susceptible to hydrolytic and thermo-oxidative degradation because of the presence of amide bonds within the polymer backbone. Elevated temperatures, ultraviolet radiation, oxidizing agents, and acidic or alkaline environments may cause chain scission, oxidation, discoloration, and deterioration of mechanical properties. These processes are accompanied by measurable changes in infrared and Raman spectra, thermal behavior, crystallinity, and surface morphology [1,124,136,175]. In contrast, polyacrylonitrile fibers generally exhibit greater resistance to hydrolysis but undergo degradation through oxidation, cyclization, dehydrogenation, and surface erosion when exposed to prolonged ultraviolet irradiation, elevated temperatures, or aggressive chemical environments. Such degradation may alter fluorescence characteristics, surface topography, and thermal stability, thereby providing additional discriminating features during forensic examination [1,136,175,176].

The evidential value of PA and PAN fibers increases considerably when degradation characteristics are interpreted together with conventional comparative fiber analyses. Modern analytical techniques, including Fourier transform infrared spectroscopy (FTIR), Raman spectroscopy, scanning electron microscopy (SEM), differential scanning calorimetry (DSC), thermogravimetric analysis (TGA), fluorescence spectroscopy, microspectrophotometry (MSP), and chromatographic methods, provide complementary information regarding both the intrinsic properties of the fibers and degradation-induced physicochemical changes [12,23]. Integrating multiple analytical techniques significantly improves the reliability of forensic interpretation while reducing the likelihood of erroneous conclusions [23,215].

Several studies have demonstrated that degradation products themselves may constitute additional discriminating characteristics. In polyamide fibers, oxidation products, changes in hydrogen bonding, crystallinity, and surface morphology may indicate exposure to heat, ultraviolet radiation, or aggressive chemicals. In polyacrylonitrile fibers, changes associated with oxidation, nitrile-group transformations, discoloration, fluorescence intensity, and thermal stability may reflect different environmental histories. Consequently, fibers that are chemically identical but have experienced different degradation pathways may be distinguished on the basis of their physicochemical characteristics [124,126,127,175,176].

Similarly, detergent residues, fluorescent whitening agents, cosmetic contaminants, soil particles, and combustion products may provide valuable contextual information. Heider et al. (2016) demonstrated that fluorescence spectroscopy combined with chemometric analysis could differentiate detergent residues adsorbed on nylon and acrylic fibers, thereby providing supplementary associative evidence [186]. Likewise, Mujumdar et al. (2015) showed that detergent-derived fluorescence significantly enhances the discrimination of textile fibers, particularly after repeated laundering [131].

For degradation evidence to be admissible in court, analytical methods must satisfy internationally accepted scientific standards. International forensic guidelines emphasize that laboratory methods should be validated with respect to accuracy, precision, repeatability, reproducibility, sensitivity, specificity, and measurement uncertainty [23]. These requirements are particularly important when degradation-induced changes are interpreted as forensic evidence because similar physicochemical alterations may result from multiple degradation mechanisms.

Despite substantial scientific advances, degradation analysis of polyamide and polyacrylonitrile fibers has several important limitations. First, degradation processes are rarely specific. Oxidation observed in polyamide fibers may result from ultraviolet radiation, elevated temperatures, or oxidizing chemicals, while hydrolysis may occur under acidic, alkaline, or humid conditions. Likewise, oxidation and cyclization reactions observed in acrylic fibers may originate from different thermal or photo-oxidative processes. Therefore, assigning a unique degradation mechanism solely on the basis of one analytical observation may lead to incorrect conclusions [23,136,215].

Second, degradation rates depend on polymer composition, degree of crystallinity, molecular orientation, stabilizing additives, dyes, manufacturing technology, humidity, oxygen concentration, temperature, irradiation intensity, and exposure duration. Even polyamide fibers of the same nominal type (e.g., PA6 or PA66) may exhibit different degradation behavior owing to differences in molecular weight or additive composition. Similar variability has also been reported for acrylic fibers containing different comonomers, pigments, and stabilizers [1,136,175].

Another important limitation concerns the complexity of real forensic environments. Laboratory simulations generally investigate isolated degradation factors under controlled conditions, whereas fibers recovered from crime scenes are commonly exposed simultaneously to sunlight, moisture, fluctuating temperatures, atmospheric pollutants, microorganisms, repeated laundering, and mechanical abrasion. The synergistic interactions among these factors remain insufficiently characterized, making direct extrapolation from laboratory experiments to forensic casework difficult [23,215].

Routine textile use further complicates interpretation. Washing, bleaching, ironing, dry cleaning, cosmetic products, body fluids, and environmental contamination may modify the physicochemical properties of both polyamide and acrylic fibers independently of degradation associated with criminal events. Consequently, distinguishing normal aging from crime-related degradation requires careful interpretation supported by multiple complementary analytical techniques [23,131,186].

Fiber evidence should therefore be presented using balanced scientific language that accurately reflects its evidential strength. Observed degradation patterns should be interpreted as being consistent with particular exposure conditions rather than as definitive proof of a specific environmental history. Evaluative reporting and likelihood ratio approaches provide the most appropriate framework for expressing the strength of degradation evidence while maintaining scientific objectivity and transparency [23,200,216].

## 11. Conclusions

The physicochemical properties of polyamide and acrylic fibers are determined by the interplay between molecular composition, condensed structure, intermolecular interactions, molecular ordering, fiber orientation, and processing history. These structural features collectively govern the mechanical, thermal, chemical, moisture-related, and dyeing behavior of fibers, illustrating the close relationship between fiber chemistry, supramolecular organization, and material performance. Table 9 provides a comparative overview of the molecular structure, supramolecular organization, and characteristic physicochemical properties of polyamide and acrylic fibers.

Acrylic and polyamide fibers differ in their susceptibility to degradation because of differences in the chemical structure of their polymer backbones. Polyamides contain amide bonds that interact with water and may undergo hydrolysis. Moisture sorption also promotes plasticization and, together with UV radiation, elevated temperatures, or mechanical stress, may accelerate fiber weakening and fragmentation. In PAN, the main chain consists of carbon–carbon bonds, while the nitrile groups occur as side groups. Under moderate environmental conditions, this structure is relatively resistant to hydrolysis and UV radiation. More severe exposure may nevertheless induce photo-oxidation, cyclization, crosslinking, or chain scission. The main differences in the susceptibility of polyamide and acrylic fibers to selected degradation factors are summarized in Table 10.

PAN may be more resistant than PA under many moderate exposure conditions, but this relationship is not universal. The relative stability of the two fiber types depends on the nature and intensity of exposure, water availability, temperature, and the composition of the material, including comonomers, dyes, and stabilizing additives (Table 10).

### Implications for Forensic Science

Textile fibers are ubiquitous in the human environment and are frequently encountered in forensic investigations. Owing to their widespread use in clothing, household furnishings, technical textiles, and personal protective equipment, both textiles and individual fibers constitute valuable forms of trace evidence. Their evidential significance increases when they exhibit damage resulting from wear or exposure to destructive factors, such as heat, fire, explosions, chemicals, ultraviolet radiation, or environmental weathering.

From a forensic perspective, degraded fibers represent more than merely altered materials. Their morphological, physicochemical, and chemical characteristics may preserve valuable information about the nature of the degrading agent, the intensity and duration of exposure, and the sequence of events associated with an incident. Consequently, the interpretation of degraded fibers can provide valuable insights into the reconstruction of criminal events and other incidents involving thermal or environmental damage. Analytical techniques for the examination of degraded textile fibers are presented in Table 11.

Forensic analysis of degraded polyamide and polyacrylonitrile fibers has made significant progress in recent years through the development and combined application of a range of analytical methods and techniques [12,112]. However, a number of scientific and practical challenges still limit the routine implementation of analytical workflows for degraded fibers for forensic purposes [23,112]. Addressing these limitations will be crucial for improving the evidentiary value, reliability, and forensic acceptance of degradation findings [23,203].

## 12. Current Challenges and Future Perspectives

### 12.1. Current Challenges

Current challenges in the forensic examination of degraded synthetic fibers include the lack of standardized analytical protocols, the limited availability of reference materials representing environmentally degraded fibers, insufficient validation of emerging analytical techniques, and the absence of comprehensive databases describing degradation pathways under different exposure conditions [23,112]. Addressing these challenges is essential for improving the reliability, repeatability, and evidentiary value of degradation-based forensic interpretations.

One of the main challenges is the inherently complex nature of degradation processes. Textile fibers, including polyamide (PA) and polyacrylonitrile (PAN), rarely degrade by a single mechanism. Instead, environmental exposure typically involves the simultaneous effects of ultraviolet radiation, atmospheric oxygen, moisture, temperature fluctuations, air pollutants, microorganisms, and mechanical abrasion. These factors interact synergistically, causing overlapping physicochemical changes [136,175]. Consequently, similar analytical signatures may originate from different degradation pathways or environmental conditions, reducing the precision of forensic interpretation [23].

The degradation of PA and PAN fibers is further influenced by numerous intrinsic material characteristics, including the polymer’s chemical composition, dye composition, pigments, matting agents, flame retardants, UV stabilizers, and manufacturing technology [1,136,175]. Even fibers produced from the same polymer type can exhibit significantly different degradation kinetics due to proprietary formulations, manufacturing parameters, and finishing methods [1,136]. This intrinsic variability complicates direct comparison of control samples with reference samples and underscores the need for comprehensive reference collections of commercially available textile materials [23,112].

Another significant limitation is the lack of comprehensive forensic databases describing the degradation profiles of synthetic textile fibers. Unlike DNA or fingerprint databases, there are currently no standardized repositories systematically documenting the physicochemical evolution of synthetic fibers such as PA and PAN. Creating such databases would significantly improve the objective interpretation of degradation evidence and facilitate interlaboratory consistency [23,112,217].

The next challenge is the considerable variability in experimental methodologies described in the literature. Published studies vary substantially in terms of the parameters used (radiation source, exposure time, temperature, relative humidity, oxidizing agents, atmospheric aging protocols, and sample preparation procedures) [175,176,179]. This makes direct comparison of studies difficult and limits the reproducibility of experimental results. Developing internationally harmonized aging and degradation testing protocols would therefore increase data comparability, improve method validation, and promote standardization of forensic degradation testing [23].

The limited amount of material available for forensic analysis is another significant limitation. Trace evidence laboratories often receive only a few microscopic fibers recovered from clothing, vehicles, weapons, or crime scenes. This limited sample size restricts the number of analytical techniques that can be applied and necessitates careful prioritization of non-destructive or minimally destructive methods. However, the integration of complementary microscopic, spectroscopic, thermal, chromatographic, and mass spectrometric techniques enables comprehensive characterization of both polymer composition and degradation-induced physicochemical changes [23,112,133,218].

Although degraded PA and PAN fibers are encountered in investigations involving fires, explosions, environmental exposure, and chemical incidents, relatively few forensic analysis reports have been published [217]. Consequently, current forensic knowledge is primarily based on experimentally simulated degradation studies and laboratory validation experiments rather than extensively documented forensic case studies [217,219].

### 12.2. Future Perspectives

Future research on textile fiber degradation should move beyond single-factor laboratory experiments and toward multifactor exposure models that more accurately simulate real-world forensic conditions. Simultaneous exposure to multiple degrading agents would better replicate the complex degradation processes encountered in forensic practice and improve the applicability of experimental results to real-world investigations [23,175,176,179,201,202,203,204,205,210].

The implementation of advanced analytical techniques, including time-of-flight secondary ion mass spectrometry (ToF-SIMS), nano-Fourier transform infrared spectroscopy (nano-FTIR), atomic force microscopy-based infrared spectroscopy (AFM-IR), confocal Raman microscopy, and hyperspectral imaging, is expected to significantly improve the characterization of degradation processes [112,144,159,218]. These high-resolution techniques enable the detection of subtle chemical and morphological changes in the early stages of degradation, before extensive structural damage becomes apparent, which would increase the sensitivity of forensic textile identification and comparative analyses [159,206,207,208,209,211,212,213].

The creation of international reference databases containing standardized analytical profiles of degraded textile fibers should also become a major research priority. Such databases should cover a variety of textile fibers produced by different manufacturers, encompassing a wide range of dye formulations, additives, production technologies, and controlled degradation scenarios. Comprehensive reference databases would facilitate objective comparisons between questioned and reference samples, improve interlaboratory consistency, and strengthen the scientific basis for forensic interpretation [23,217,219].

Further harmonization of analytical protocols, validation procedures, and reporting practices will be essential for the routine forensic examination of degraded textile evidence. Future interpretation of analytical results should increasingly rely on evaluative reporting frameworks based on likelihood ratios and Bayesian reasoning, enabling experts to quantify the strength of scientific evidence while accounting for uncertainty, alternative hypotheses, and the limitations of available data [23].

Artificial intelligence (AI) and machine learning (ML) are expected to play an increasingly important role in the forensic analysis of degraded textile fibers. Advanced computational algorithms can integrate complex datasets generated by spectroscopic, microscopic, thermal, chromatographic, and imaging techniques, enabling more accurate discrimination of degradation pathways and exposure conditions. Machine learning models can also support automatic fiber classification, pattern recognition, prediction of degradation mechanisms, and probabilistic interpretation of analytical results. With the emergence of large, standardized forensic datasets, AI-assisted approaches are expected to improve the objectivity, repeatability, and efficiency of forensic examinations while reducing observer-dependent variability [23,200,211,213,216].

In summary, given the influence of degradation processes on polyamide and acrylic fibers, further comprehensive research is required to clarify how these processes affect the interpretation of degraded fiber evidence. Such knowledge is essential for enhancing the reconstruction of criminal events and other incidents involving thermal, chemical, biological, use-related, or environmental degradation.

## Figures and Tables

**Table 1:** Market applications of polyamide fiber types. Data from [1,2,3,4,5,6,7,8].

Polyamide Fiber Type	Typical Commercial Designation	Main Applications
PA6	Nylon 6	Apparel, hosiery, carpets, industrial yarns
PA66	Nylon 6,6; Nylon 66	Technical textiles, airbags, tire cord, industrial fabrics
PA11	Nylon 11	High-performance textiles, specialty applications
PA12	Nylon 12	Technical fibers, filtration, medical applications
Bio-based polyamides (PA56, PA510, PA610, PA1010, etc.)	Specialty grades	Emerging sustainable textile applications

**Table 2:** Global polyamide (nylon) fiber production. Data from [8,9].

Year	Global Production (Million Tonnes)	Share of Global Fiber Production
2022	6.8	5.9%
2023	6.9	5.6%
2024	7.0	5.3%

**Table 3:** Classification of acrylic and modacrylic textile fibers according to ISO/BISFA and U.S. terminology and their typical applications. Data from [1,2,3,4,5,6,7,10].

Fiber Type	Acrylonitrile Content	Typical Applications
Acrylic	≥85 wt% acrylonitrile repeating units	Knitwear, blankets, upholstery, apparel
Modacrylic	≥50 but <85 wt%—ISO/BISFA; ≥35 but <85 wt%—U.S. definition	Flame-resistant protective clothing, wigs, faux fur

**Table 4:** Comparative assessment of analytical techniques used in the forensic examination of degraded textile fibers.

Analytical Technique/Method	Sample Requirements	Information Obtained	Advantages in Forensic Examination	Limitations in Forensic Examination	Role in Forensic Examination
**Optical microscopy/PLM**	Single fiber	Fiber morphology, diameter, cross-sectional features, optical properties, birefringence	− Rapid, non-destructive method − Minimal sample preparation − Individual fibers can be examined − Direct comparison with reference material	− Limited ability to differentiate fibers with similar morphology − Degradation may alter optical properties	Primary screening and comparison method
**Fluorescence microscopy**	Single fiber	Fluorescence properties of the fiber and/or dye	− Rapid, non-destructive method − Useful for differentiating visually similar fibers	− Fluorescence properties may be altered by environmental factors, detergents, and chemicals	Complementary screening and discrimination method
**SEM/** **SEM–EDS**	Single fiber, yarn, or textile fragment	High-resolution surface morphology (e.g., cracks, deposits); elemental composition when EDS is used	− Minimally destructive, depending on sample preparation − Very high spatial resolution − Particularly useful for assessing surface damage − Identification of inorganic components	− Images are generally grayscale − No direct information on color or the composition of organic dyes − May require specific sample preparation	Preferred method for detailed characterization of surface damage
**FTIR/** **µ-FTIR**	Single fiber, yarn, or textile fragment	Identification of the fiber-forming polymer	− Rapid; non-destructive or minimally destructive depending on the measurement mode − Identification of the fiber-forming polymer	− Low sensitivity to dyes and components present at low concentrations − Degradation-related spectral changes may be absent or subtle − Insufficient as a stand-alone method for comprehensive characterization of degraded fibers	Primary method for identification of the fiber-forming polymer
**Raman spectroscopy**	Single fiber	Polymer structure, dyes and pigments, and degradation-related chemical changes	− Usually non-destructive − Minimal sample preparation − Can provide information on both the polymer and the colorant − Complementary to FTIR	− Fluorescence may mask the Raman signal − Laser irradiation may alter sensitive or severely degraded samples − Complex spectra may be difficult to interpret	Complementary characterization of polymer and dye; particularly useful for colored fibers
**SERS/** **SERRS**	Single fiber or dye extract	Information on dyes and pigments present at very low concentrations	− Substantially enhanced Raman sensitivity − Enables the analysis of weak dye signals	− More complex sample preparation − Variability associated with the SERS substrate − Possible modification of the sample surface − Dye extraction may be required in some procedures − Limited routine forensic implementation	Advanced complementary technique
**UV–Vis MSP**	Single fiber	Spectral characterization of color and chromophoric components	− Non-destructive − Useful for objective color comparison − Can reveal differences not visible to the naked eye	− Does not directly identify the fiber-forming polymer − Spectra may change as a result of dye fading and degradation	Preferred method for objective color comparison
**DSC**	Small but measurable amount of sample	Melting temperature, glass transition temperature, crystallization behavior, and degree of crystallinity	− Provides information on thermal properties and structural organization − Useful when microscopy and spectroscopy do not provide sufficient discrimination	− Destructive − Requires more material than microscopic and spectroscopic methods − Results may depend on the prior thermal history of the sample	Complementary thermal characterization
**TGA**	Small bulk sample or several fibers	Thermal stability, decomposition profile, moisture content, additives, and fillers	− Provides characteristic decomposition profiles − Useful for assessing thermal stability	− Destructive − Requires more material − Limited applicability to routine examination of individual fibers − Decomposition profiles may be modified by additives, dyes, and finishing agents	Complementary assessment of thermal stability
**TLC**	Single fiber or several fibers	Comparison of extracted dye composition	− Simple and relatively inexpensive − Enables comparison of components in dye mixtures	− Destructive − Requires dye extraction − Lower structural specificity than modern chromatographic techniques coupled with MS	Screening method for dye analysis
**HPLC–UV/DAD**	Single fiber or several fibers	Separation, identification, and comparison of extracted dyes and their components	− Greater discriminatory power than visual or purely spectroscopic comparison − Identification and analysis of dyes − Useful for complex dye mixtures	− Destructive − Requires extraction − Limited applicability when only a very small amount of evidential material is available	Advanced confirmatory method for dye analysis
**LC–MS/MS/** **LC–HRMS**	Single fiber or several fibers	Molecular identification of dyes, additives, and selected degradation products	− High discriminatory power − Identification and analysis of dyes − Useful for complex dye mixtures	− Destructive − Requires extraction of dyes/components − Greater instrumental and interpretative complexity − Limited reference data for degraded fibers	High-specificity confirmatory analysis
**Py–GC/MS**	Single fiber or several fibers	Polymer and copolymer composition, selected additives, and characteristic pyrolysis products	− High chemical specificity − Identification and differentiation of polymers based on characteristic pyrolysis products − May differentiate morphologically similar fibers and fibers within the same polymer class	− Completely destructive for the analyzed portion of the sample − Results depend on pyrolysis conditions and instrumental parameters − Requires specialized instrumentation and data interpretation	Confirmatory and discriminative method for polymer composition analysis, particularly valuable when non-destructive methods do not provide sufficient discrimination

**Table 5:** Polyacrylonitrile fibers subjected to various thermal factors. Adapted from Wąs-Gubała, J., *Wpływ wybranych czynników na destrukcję włókien i wyrobów włókienniczych w aspekcie ich znaczenia w procesie sądowym*; Wydawnictwo Instytutu Ekspertyz Sądowych: Kraków, Poland, 2009 [124], with permission from the publisher Institute of Forensic Research.

Experiment	Scanning Electron Microscope Images	
VCE	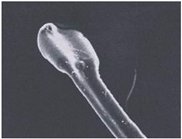	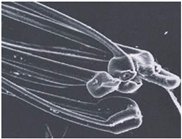
a heated flat metal surface	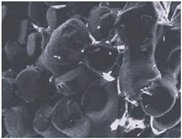	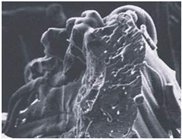
flame exposure	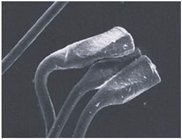	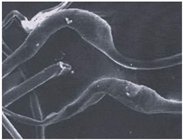

**Table 6:** Polyamide fibers subjected to various thermal factors. Adapted from Wąs-Gubała, J., *Wpływ wybranych czynników na destrukcję włókien i wyrobów włókienniczych w aspekcie ich znaczenia w procesie sądowym*; Wydawnictwo Instytutu Ekspertyz Sądowych: Kraków, Poland, 2009 [124], with permission from the publisher Institute of Forensic Research.

Experiment	Scanning Electron Microscope Images	
VCE	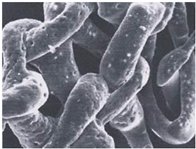	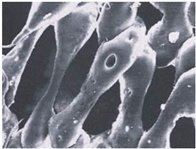
a heated flat metal surface	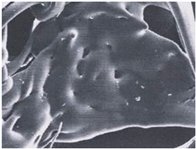	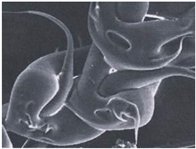
flame exposure	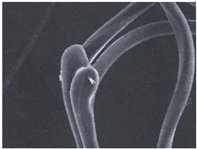	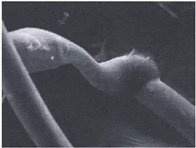

**Table 7:** Microscopic images of reference undyed and unfinished polyacrylonitrile fibers and fibers treated with a detergent solution prepared in distilled water, acquired using UV filter. Adapted from Wąs-Gubała, J., *Wpływ wybranych czynników na destrukcję włókien i wyrobów włókienniczych w aspekcie ich znaczenia w procesie sądowym*; Wydawnictwo Instytutu Ekspertyz Sądowych: Kraków, Poland, 2009 [124], with permission from the publisher Institute of Forensic Research.

Reference Material	Day 3	Day 7
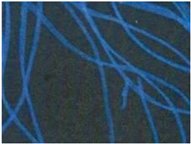	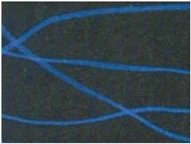	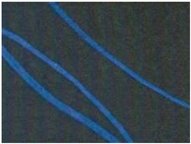
	**Day 10**	**Day 14**
	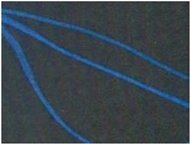	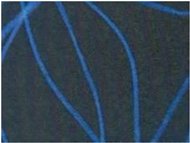

**Table 8:** Comparison of the color of untreated blue polyacrylonitrile fibers and fibers treated with a detergent solution prepared in distilled water, observed by fluorescence microscopy using a UV filter. Adapted from Wąs-Gubała, J., *Wpływ wybranych czynników na destrukcję włókien i wyrobów włókienniczych w aspekcie ich znaczenia w procesie sądowym*; Wydawnictwo Instytutu Ekspertyz Sądowych: Kraków, Poland, 2009 [124], with permission from the publisher Institute of Forensic Research.

Reference Material	Day 1	Day 14
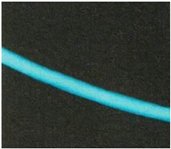	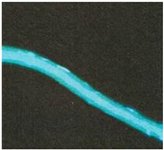	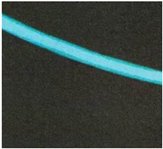

**Table 9:** Comparative overview of the molecular structure, supramolecular organization, and characteristic physicochemical properties of polyamide and acrylic fibers.

Feature	Polyamide Fibers (PA6/PA66)	Acrylic Fibers (PAN-Based)
**Molecular structure**	Repeating amide (–CONH–) groups; extensive hydrogen bonding; α-, β-, and γ-polymorphism	PAN backbone containing nitrile (–C≡N) groups; strong dipole–dipole interactions; predominantly atactic planar zigzag chains
**Supramolecular organization**	Hydrogen-bonded sheets; lamellar stacks; semicrystalline morphology	Rod-like chains; pseudo-hexagonal packing; paracrystalline domains; fibrillar morphology
**Characteristic physicochemical properties**	High tensile strength; toughness; moderate moisture regain; chemical resistance; good dyeability	Wool-like elasticity and good elastic recovery; low moisture regain; chemical resistance; good dyeability after copolymer modification

**Table 10:** Comparison of the main degradation pathways of polyamide and acrylic fibers under selected exposure conditions.

Factor	Polyamide Fibers	Acrylic Fibers
Water and moisture	Water sorption, plasticization, and possible hydrolysis of amide bonds	Low susceptibility of the main chain to hydrolysis; transformations mainly involve nitrile groups under more severe conditions
Acidic and alkaline hydrolysis	Possible cleavage of backbone amide bonds and shortening of macromolecules	Conversion of –C≡N groups into amide and carboxyl groups; the carbon backbone is not directly hydrolyzed
Moderate UV exposure	Photo-oxidation and fragmentation, enhanced by moisture	Often only minor changes in fiber integrity; slow photo-oxidation may occur
Intense UV exposure	Oxidation, chain scission, and loss of mechanical properties	Photo-oxidation, cyclization, crosslinking, or chain scission
Elevated temperature	Thermo-oxidative chain scission and a decrease in molecular weight	Cyclization, dehydrogenation, oxidation, and crosslinking; fragmentation may occur under severe conditions
Strong oxidizing agents	Possible substantial loss of mechanical properties and fiber integrity	Greater resistance in many systems, although oxidation of functional groups may occur

**Table 11:** Analytical techniques for the examination of degraded textile fibers.

Morphology	Fiber-Forming Polymer	Dyes, Pigments, Additives, Finishing and Degraded Agents
*Optical microscopy techniques:* ▪ biological microscopy ▪ polarized light microscopy ▪ fluorescence microscopy *Scanning electron microscopy*	*Spectroscopic techniques:* ▪ Fourier-transform infrared spectroscopy (FTIR) ▪ Raman spectroscopy *Thermal analysis techniques:* ▪ differential scanning calorimetry (DSC) ▪ thermogravimetric analysis (TGA)	*Spectroscopic techniques:* ▪ UV–Vis microspectrophotometry (MSP UV–Vis) ▪ Raman spectroscopy *Chromatography techniques:* ▪ thin-layer chromatography (TLC) ▪ high-performance liquid chromatography (HPLC), often coupled with UV–visible detection, diode array detection (DAD), or mass spectrometry (LC–MS/MS, HRMS) ▪ gas chromatography coupled with pyrolysis and mass spectrometry Py-GC/MS

## Data Availability

No new data were created or analyzed in this study. Data sharing is not applicable.

## Abbreviations

The following abbreviations are used in this manuscript:

AI	Artificial intelligence
DAD	Diode-Array Detection
DSC	Differential Scanning Calorimetry
EDS	Energy-Dispersive X-ray Spectroscopy
EEM	Excitation–Emission Matrix Fluorescence
FTIR	Fourier-Transform Infrared Spectroscopy
GC–Py/MS	Pyrolysis Gas Chromatography–Mass Spectrometry
HPLC	High-Performance Liquid Chromatography
ML	Machine Learning
MSP	Microspectrophotometry
MSP UV–Vis	UV–Vis Microspectrophotometry
PA	Polyamide
PA6	Nylon 6
PA66	Nylon 66/Nylon 6,6
PAN	Polyacrylonitrile
PCA	Principal Component Analysis
PLM	Polarized Light Microscopy
SEM	Scanning Electron Microscopy
SEM-EDS	Energy-Dispersive X-ray Spectroscopy
SERRS	Surface-Enhanced Resonance Raman Scattering
SERS	Surface-Enhanced Raman Spectroscopy
TGA	Thermogravimetric Analysis
TLC	Thin-Layer Chromatography
UV	Ultraviolet
VCE	Vapour Cloud Explosion
XRPD	X-ray Powder Diffraction
